# A non-carboxylating pentose bisphosphate pathway in halophilic archaea

**DOI:** 10.1038/s42003-022-04247-2

**Published:** 2022-11-24

**Authors:** Takaaki Sato, Sanae (Hodo) Utashima, Yuta Yoshii, Kosuke Hirata, Shuichiro Kanda, Yushi Onoda, Jian-qiang Jin, Suyi Xiao, Ryoko Minami, Hikaru Fukushima, Ayako Noguchi, Yoshiyuki Manabe, Koichi Fukase, Haruyuki Atomi

**Affiliations:** 1 Department of Synthetic Chemistry and Biological Chemistry, Graduate School of Engineering, Kyoto University, Kyoto, Japan; 2 Integrated Research Center for Carbon Negative Science, Kyoto University, Kyoto, Japan; 3 Department of Chemistry, Graduate School of Science, Osaka University, Osaka, Japan; 4 Forefront Research Center, Osaka University, Osaka, Japan

**Keywords:** Archaeal physiology, Kinases

## Abstract

Bacteria and Eucarya utilize the non-oxidative pentose phosphate pathway to direct the ribose moieties of nucleosides to central carbon metabolism. Many archaea do not possess this pathway, and instead, Thermococcales utilize a pentose bisphosphate pathway involving ribose-1,5-bisphosphate (R15P) isomerase and ribulose-1,5-bisphosphate (RuBP) carboxylase/oxygenase (Rubisco). Intriguingly, multiple genomes from halophilic archaea seem only to harbor R15P isomerase, and do not harbor Rubisco. In this study, we identify a previously unrecognized nucleoside degradation pathway in halophilic archaea, composed of guanosine phosphorylase, ATP-dependent ribose-1-phosphate kinase, R15P isomerase, RuBP phosphatase, ribulose-1-phosphate aldolase, and glycolaldehyde reductase. The pathway converts the ribose moiety of guanosine to dihydroxyacetone phosphate and ethylene glycol. Although the metabolic route from guanosine to RuBP via R15P is similar to that of the pentose bisphosphate pathway in Thermococcales, the downstream route does not utilize Rubisco and is unique to halophilic archaea.

## Abstract (web summary)

A nucleoside degradation pathway in halophilic archaea is identified, which converts the ribose moiety of guanosine to dihydroxyacetone phosphate and ethylene glycol.

## Introduction

It is well established that Archaea display unique metabolic enzymes and pathways not found in Bacteria and Eucarya^1–3^. A representative example is pentose metabolism. Bacteria and eukaryotes utilize the pentose phosphate pathway^4^ to synthesize or degrade the pentose moieties of nucleic acids. The oxidative pentose phosphate pathway (OPP pathway) synthesizes the nucleic acid precursors ribulose 5-phosphate (Ru5P) and ribose 5-phosphate (R5P) from glucose 6-phosphate as well as providing reducing equivalents in the form of NADPH. The non-oxidative pentose phosphate pathway (NOPP pathway) carries out the interconversion between pentoses (Ru5P and R5P) and fructose 6-phosphate and glyceraldehyde 3-phosphate and can thus convert the ribose moieties of nucleosides to intermediates of central carbon metabolism (Supplementary Fig. 1a). However, many archaea do not possess the OPP and NOPP pathways. They instead utilize the ribulose monophosphate (RuMP) pathway to produce the pentoses necessary for nucleic acid biosynthesis^5–7^, converting fructose 6-phosphate to Ru5P and formaldehyde. As exceptions, most halophilic archaea possess the OPP pathway^7–10^, while a number of archaeal species including members of Thermoplasmatota, Thaumarchaeota, *Halorhabdus*, *Methanococcus*, and *Methanocaldococcus* are predicted to harbor gene homologs constituting the NOPP pathway. It should be noted, however, that although *Methanocaldococcus jannaschii* harbors both the NOPP pathway and the RuMP pathway^11^, R5P seems to be generated by the RuMP pathway^12^.

**Fig. 1:**
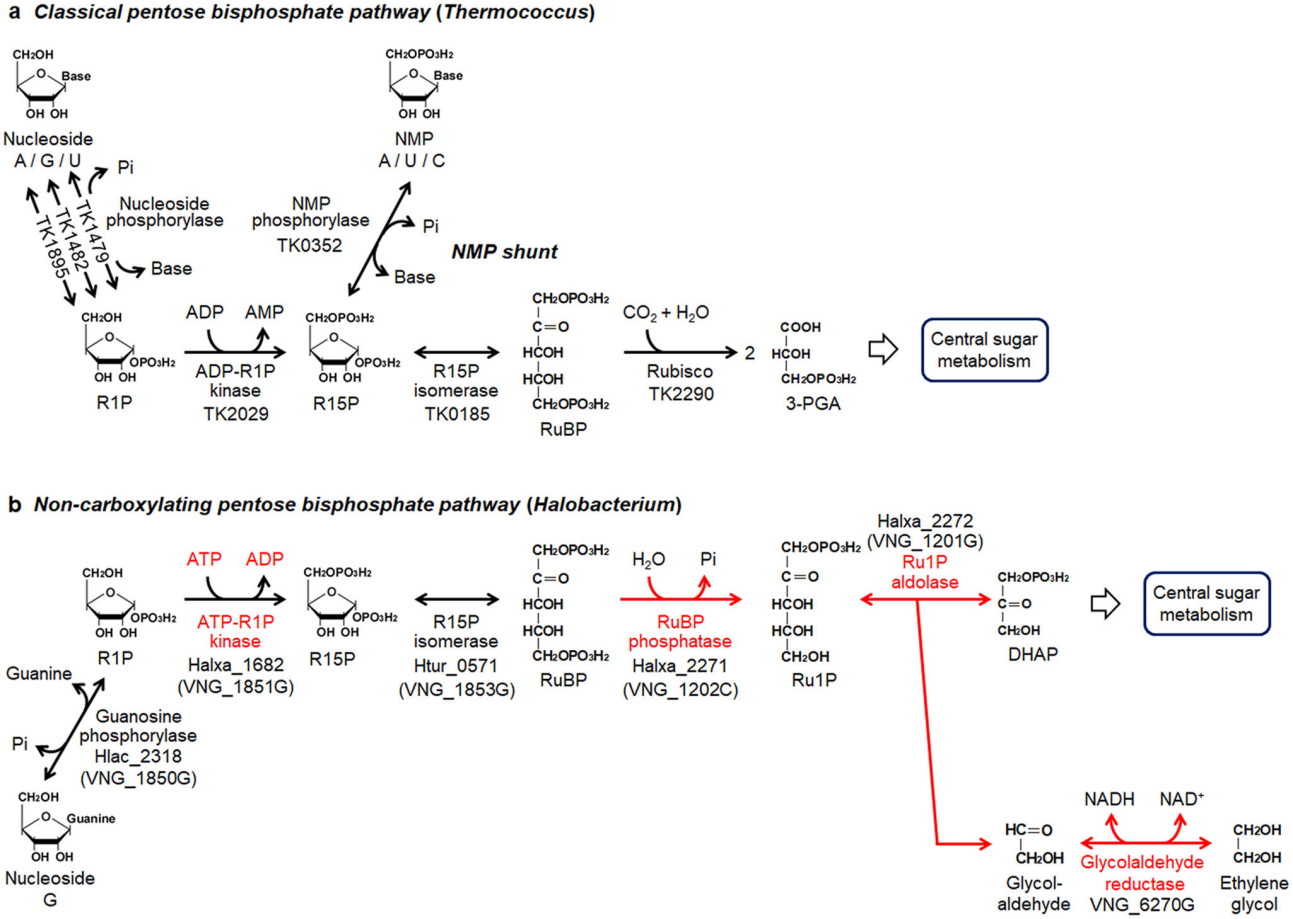
Schematic drawing of archaeal nucleoside metabolic pathways in *Thermococcus* and in halophilic archaea including *Halobacterium*. **a** The classical pentose bisphosphate pathway identified in *Thermococcus* is predicted to be involved in the nucleoside degradation and/or the conversion of nucleosides to NMPs. The NMP shunt, composed of NMP phosphorylase, R15P isomerase, and Rubisco, degrades NMPs to 3-PGA. **b** The non-carboxylating pentose bisphosphate pathway proposed in this study is shown. Red arrows indicate reactions specific to the pathway identified in this study and absent in the classical pentose bisphosphate pathway. The locus tags encoding the enzymes whose recombinant proteins were examined in this study are shown. Locus tags with parenthesis are genes predicted to encode enzymes whose activities were detected in the cell-free extract from *H. salinarum*. Regarding VNG_6270G, both native and recombinant enzymes were investigated. NMP nucleoside-5’-monophosphate, R1P ribose-1-phosphate, R15P ribose-1,5-bisphosphate, RuBP ribulose-1,5-bisphosphate, 3-PGA 3-phosphoglycerate, Ru1P ribulose-1-phosphate, DHAP dihydroxyacetone phosphate.

As for nucleoside degradation, the hyperthermophilic archaeon *Thermococcus kodakarensis* utilizes a pentose bisphosphate pathway^13–15^ (Fig. 1a and Supplementary Fig. 1b). The metabolites of the pathway greatly differ to those of the pentose phosphate pathway and a number of unique enzymes are involved. As in bacteria and eukaryotes, nucleosides are converted to ribose-1-phosphates (R1P) and nucleobases by three nucleoside phosphorylases that recognize uridine, guanosine, and adenosine (TK1479, TK1482, and TK1895, respectively). While R1P is converted to R5P in the pentose phosphate pathway, R1P is phosphorylated by an ADP-dependent ribose-1-phosphate kinase (ADP-R1PK; TK2029) in *T. kodakarensis*, generating ribose-1,5-bisphosphate (R15P). R15P isomerase (TK0185) then converts R15P to ribulose-1,5-bisphosphate (RuBP), which is subsequently converted to 3-phosphoglycerate (3-PGA) by the carboxylase activity of ribulose-1,5-bisphosphate carboxylase/oxygenase (Rubisco; TK2290)^16–19^. *T. kodakarensis* also harbors a unique nucleoside-5’-monophosphate phosphorylase (NMP phosphorylase, previously designated AMP phosphorylase; TK0352) that catalyzes the phosphorolysis of AMP, CMP, and UMP^14,15^. This allows the direct conversion of NMPs to R15P, as well as the conversion of nucleosides to NMPs via R1P and R15P (Fig. 1a and Supplementary Fig. 1b). R15P thus acts as a node that connects nucleoside and nucleotide metabolism with central carbon metabolism^13,20^.

When the distribution of the pentose bisphosphate pathway is examined, homologs of NMP phosphorylase, R15P isomerase, and Rubisco are found in a wide range of archaea; most members of Archaeoglobales, Lokiarchaeota, Methanomicrobiales, Methanosarcinales, and Thermococcales as well as some members in Desulfurococcales, Halobacteriales, Methanococcales, and most *Thermofilum* species in Thermoproteales. In addition, it was recently reported that metagenomic analysis suggested the presence of these genes in a number of bacterial species^21^. This raises the possibilities that this portion of the pentose bisphosphate pathway, which we refer to here as the “NMP shunt”, functions in many archaeal and some bacterial species. On the other hand, the distribution of ADP-R1PK homologs seems to be limited to members of the Thermococcales, which include the genera *Palaeococcus*, *Pyrococcus*, and *Thermococcus*. However, it is well known that the ADP-dependent kinases in Thermococcales have counterparts in other archaea that are dependent on ATP as the phosphate donor. These include the glucokinases^22–24^ and phosphofructokinases^25,26^ in glycolysis, serine kinase in amino acid metabolism^27,28^, and even R1P kinase itself^13,29^. These ATP- and ADP-dependent kinases that recognize the same phosphate acceptor usually do not display similarity to each other. Therefore, it is difficult to accurately conclude the distribution of R1P kinase activity based solely on genome sequences. There is a large number of proteins in archaea that are annotated as sugar kinases whose phosphate acceptors have not been identified, and these might include unidentified R1P kinases.

Intriguingly, we found that a number of halophilic archaea harbor R15P isomerase homologs, but do not have homologs for NMP phosphorylase and Rubisco. In addition, clear-cut homologs of R1P kinase are not present in any of the halophile genomes. The genome sequences thus suggest that in these halophiles, there are no enzymes that would supply the substrate or utilize the product of R15P isomerase. In this study, we searched for enzymes linked to the apparently standalone R15P isomerase, and have identified a previously unknown metabolic pathway that involves the pentose bisphosphates R15P and RuBP in halophilic archaea.

## Results

### Examination of R15P isomerase homologs from halophilic archaea

This study was initiated prior to the discovery of the entire pentose bisphosphate pathway, when only a route from NMPs to 3-PGA consisting of NMP phosphorylase, R15P isomerase, and Rubisco was known^14,15^ (Fig. 1a). A number of archaeal species harbored homologs of all three enzymes, but intriguingly, some halophilic archaea harbored only R15P isomerase homologs^15^. These include *Halobacterium salinarum* that possesses the VNG_1853G protein, which is 48.1% identical with R15P isomerase from *T. kodakarensis* (TK0185), but does not harbor homologs of NMP phosphorylase nor Rubisco. Among 63 halophilic archaea shown in Table 1, such cases are observed in 19 species (Table 1, red or orange in VNG_1853G column). On the other hand, 14 halophilic archaea harbor a complete set of homologs (blue in TK0352/VNG_1853G/TK2290 columns), while 11 species possess only R15P isomerase and Rubisco homologs (green in VNG_1853G/TK2290 columns).

**Table 1:**
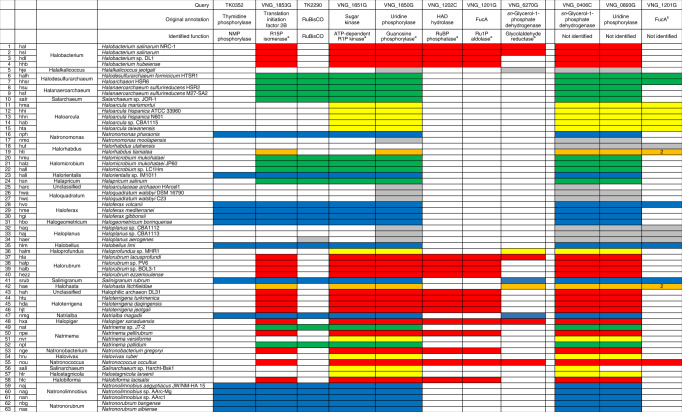
Distribution of nucleoside/NMP metabolic genes in halophilic archaea.

Colored boxes indicate the presence of genes encoding each enzyme on the genome. Black numbers indicate that two homologs are present.

^a^Enzyme activities demonstrated in this study.

^b^These FucA homologs are phylogenetically distinguished from the FucA homologs in the seventh column, which are specific to halophilic archaea harboring standalone R15P isomerase and HAD hydrolase (Supplementary Fig. 10). The FucA homologs in the eleventh column are referred to as FucA* in the text.

**Fig. 2:**
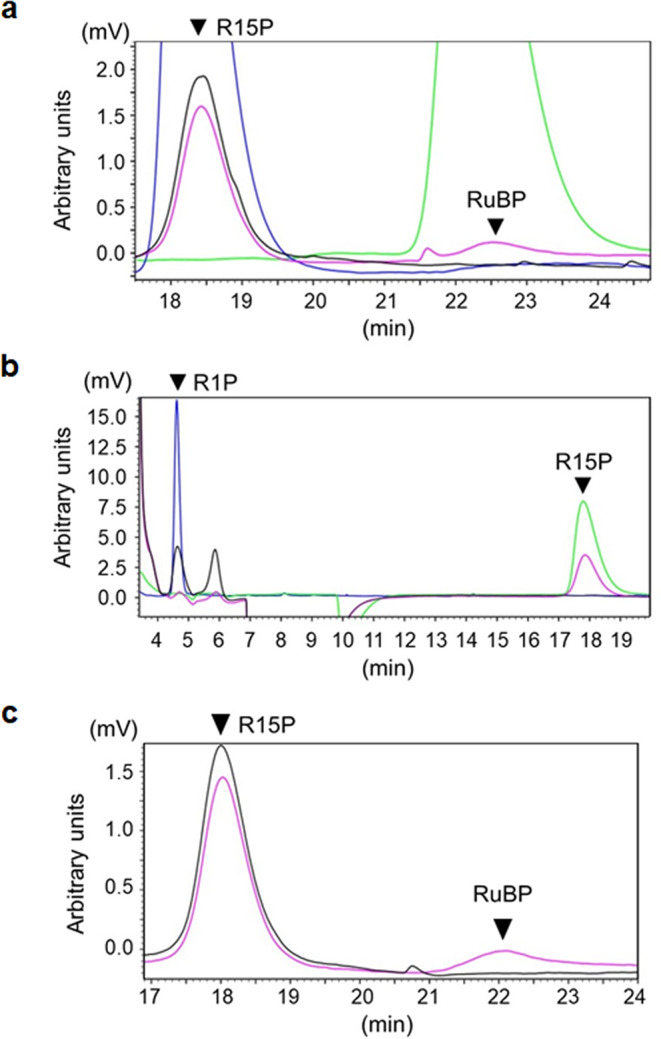
Enzyme activity analyses of *Ht*-R15P isomerase and *Hx*-RbsK proteins with HPLC. **a** In the presence or absence of *Ht*-R15P isomerase enzyme, the generation of RuBP from R15P was investigated. Black, pink, blue, and green lines indicate the reaction product without the enzyme, that with the enzyme, 20 mM R15P standard compound, and 10 mM RuBP standard compound, respectively. **b** In the presence or absence of the *Hx*-RbsK protein, the generation of R15P from R1P was examined. Black, pink, blue, and green lines indicate the reaction product without the enzyme, that with the enzyme, 10 mM R1P standard compound, and 20 mM R15P standard compound, respectively. **c** Conversion of R1P with the *Hx*-RbsK and *Ht*-R15P isomerase proteins was examined. After the kinase reaction by *Hx*-RbsK, the isomerase reaction by *Ht*-R15P isomerase was carried out. The reaction product was analyzed by HPLC. Pink and black lines indicate reaction products with and without *Ht*-R15P isomerase in the second reaction, respectively. Compounds separated with a column were monitored with a differential refractive index detector.

We examined whether the R15P isomerase homologs actually catalyze the ribose-1,5-bisphosphate isomerase reaction. The corresponding genes from *H. salinarum* (VNG_1853G) and *Haloterrigena turkmenica* (Htur_0571) were overexpressed in *Escherichia coli*. Only the Htur_0571 protein from *H. turkmenica* could be obtained in a soluble form and was purified to apparent homogeneity (Supplementary Fig. 2a). RuBP was generated from R15P in the presence of purified Htur_0571 protein (Fig. 2a), indicating that the protein displayed R15P isomerase activity and was designated *Ht*-R15P isomerase. The result suggested the presence of unknown pathway(s) involving R15P and RuBP in halophilic archaea.

### Search for genes encoding enzymes linked to R15P isomerase

**Fig. 3:**
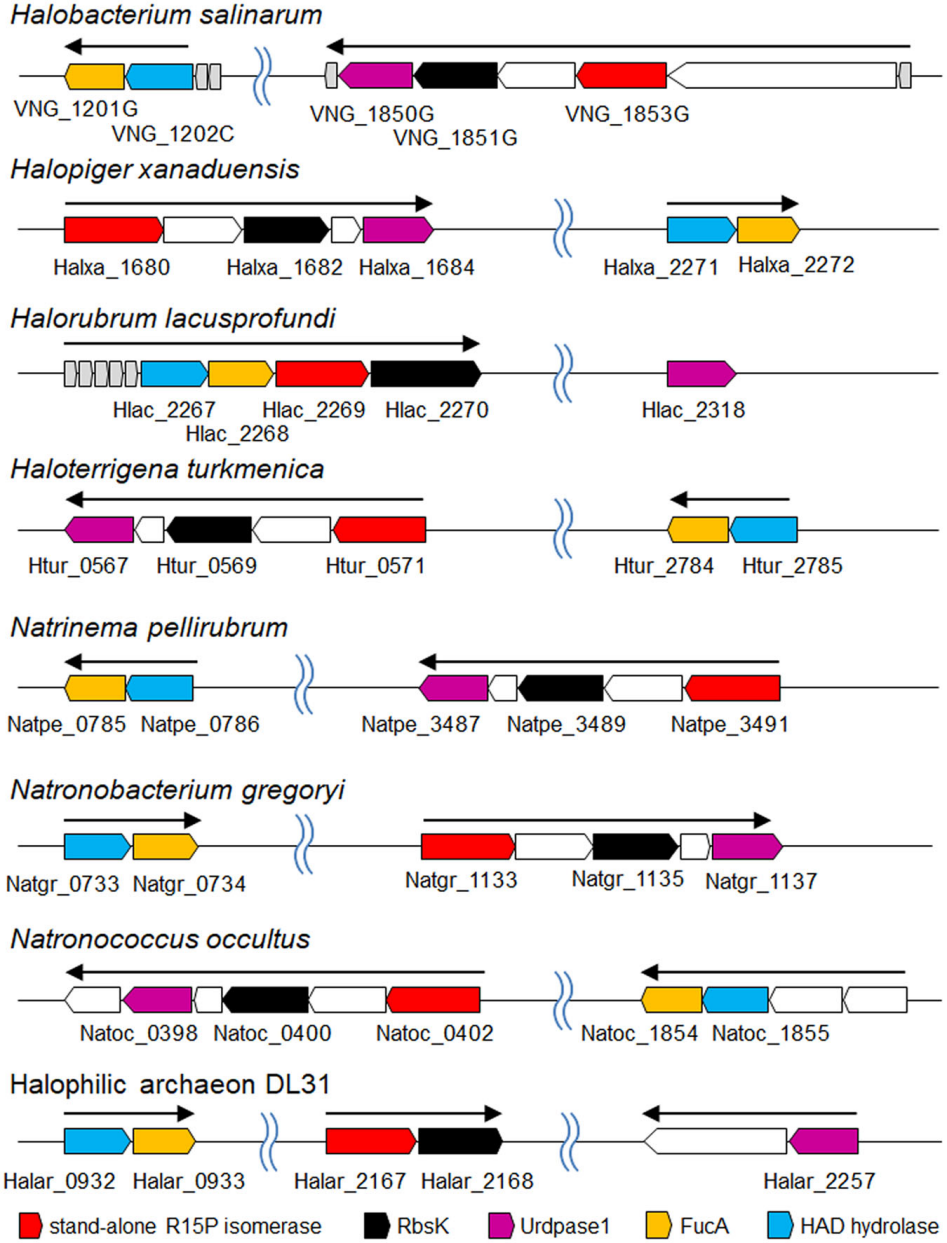
Schematic diagram of gene arrangement on the eight haloarchaeal genomes. Color codes of relevant genes are indicated in the figure. White and gray-arrowed boxes are genes most likely forming operons with the focused five genes. In the gray-arrowed boxes, the gene length is not reflected to the width of each arrowed box. Black arrows indicate predicted operons.

In order to identify enzymes involved in the metabolism of R15P or RuBP, candidate genes were searched using the genome sequences. Halophilic archaea with the standalone R15P isomerase homolog include *H. salinarum*^30^, *Halopiger xanaduensis*^31^, *Halorubrum lacusprofundi*^32^, *H. turkmenica*^33^, halophilic archaeon DL31, *Natrinema pellirubrum*, *Natronobacterium gregoryi*, and *Natronococcus occultus*. We looked for genes forming an operon with the R15P isomerase homolog in these 8 species and found that genes annotated as “sugar kinase, ribokinase”/“PfkB domain protein” (*rbsK*) formed operons with the R15P isomerase homologs in all eight cases, while “uridine phosphorylase” (*urdpase*) genes formed operons in six cases (Fig. 3). Among the 19 genomes that harbor an R15P isomerase homolog but not a Rubisco and NMP phosphorylase homolog, all, with the exception of *Halorhabdus tiamatea*, possess both *rbsK* and *urdpase* homologs (Table 1, VNG_1851G/VNG_1850G columns). This suggested that the two gene products are metabolically linked with R15P isomerase.

### Identification of an ATP-dependent ribose-1-phosphate kinase

Recombinant RbsK proteins from *H. salinarum*, *H. turkmenica*, and *H. xanaduensi*s (*Hs*-RbsK, *Ht*-RbsK, and *Hx*-RbsK encoded by VNG_1851G, Htur_0569, and Halxa_1682, respectively) were produced in *E. coli*. *Hs*-RbsK formed inclusion bodies and expression levels of the *Ht*-RbsK gene were low. Sufficient amounts of soluble *Hx*-RbsK protein were obtained, and partially purified (Supplementary Fig. 3a). Kinase activities of the partially purified *Hx*-RbsK protein were tested toward various phosphate acceptor substrates (Supplementary Table 1) using ATP or a mixture of nucleoside triphosphates (NTPs) as a phosphate donor (Supplementary Fig. 4). We examined 85 substrates including nucleosides, aldoses, amino sugars, alcohols, ketoses, nucleotides, sugar phosphates, disaccharides, sugar alcohols, and deoxy sugars. When normalized by reaction time and amount of enzyme, production of nucleoside-5’-diphosphate (NDP) was highest with ribose-1-phosphate (R1P) (468 nmol ADP min^−1^ mg^−1^). We refrain from designating the values of these measurements as reaction rates, as the product formation rate is not necessarily constant throughout the reaction period. The next highest value was observed with xylulose (62 nmol NDP min^−1^  mg^−1^), followed by 2’-deoxyguanosine (57 nmol NDP min^−1^ mg^−1^) and d-ribulose (49 nmol NDP min^−1^ mg^−1^). The *Hx*-RbsK recombinant protein was purified for further biochemical analyses (Supplementary Fig. 2b). Using R1P as the phosphate acceptor, the enzyme preferred ATP among NTPs (Supplementary Fig. 5a). HPLC analysis of the *Hx*-RbsK reaction product indicated that R15P was generated from R1P (Fig. 2b). In addition, the *Hx*-RbsK product, R15P, was isomerized to RuBP by *Ht*-R15P isomerase (Fig. 2c). *Hx*-RbsK required salt for its kinase activity, and 2.0 M was the optimal KCl concentration (Supplementary Fig. 5b). In the presence of 4 mM ATP and 20 mM R1P, we observed a specific activity of 30.1 μmol min^−1^ mg^−1^. The analyses on the Halxa_1682 protein indicated that the protein *Hx*-RbsK is an ATP-dependent ribose-1-phosphate kinase (ATP-R1PK) generating the substrate for R15P isomerase.

### Identification of a guanosine phosphorylase

Almost all genomes of halophilic archaea harbor two proteins annotated as uridine phosphorylase (Table 1, VNG_1850G/VNG_0893G columns). VNG_1850G homologs form an operon with the R15P isomerase and ATP-R1PK genes in some species, while the positions of VNG_0893G homologs are not related with the operon. The former is designated here as Urdpase1 and the latter Urdpase2. They can be distinguished phylogenetically (Supplementary Fig. 6), suggesting that their functions and/or enzymatic properties are distinct. In this study, the Urdpase1 protein was investigated. Although we assumed that Urdpase1 catalyzes a nucleoside phosphorylase reaction and generates R1P, the substrate for ATP-R1PK, it was unclear which nucleosides are recognized by the enzyme.

**Fig. 4:**
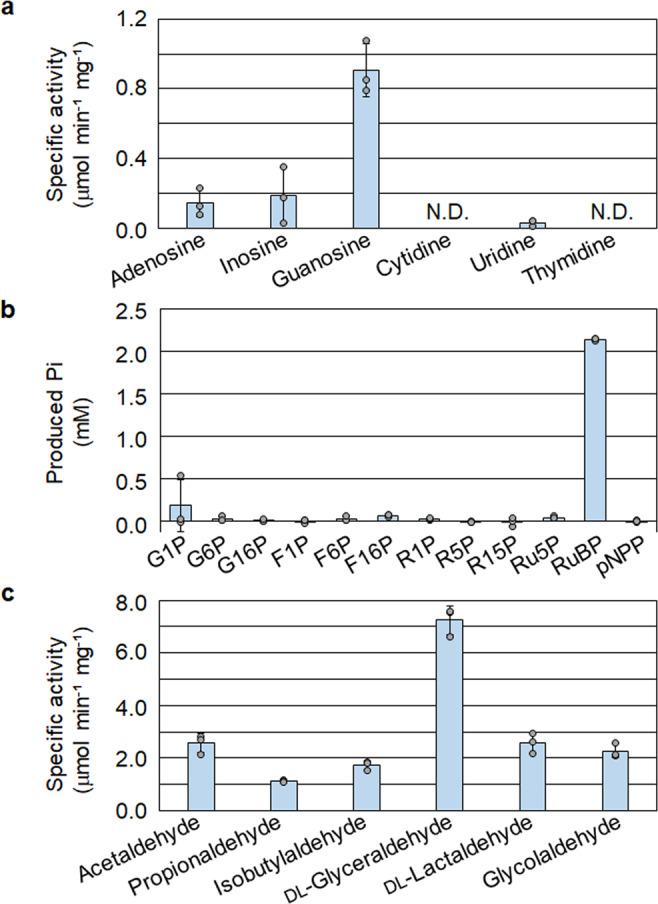
Enzyme activity analyses of *Hl*-Urdpase1, *Hx*-HAD hydrolase, and *Hx*-FucA proteins. **a** Nucleoside phosphorylase activity of *Hl*-Urdpase1 was analyzed toward six nucleosides by quantifying the released nucleobases with HPLC. **b** Phosphatase activity of *Hx*-HAD hydrolase was investigated toward eleven sugar phosphates and pNPP by quantifying released phosphate with malachite green. G1P glucose-1-phosphate, G6P glucose-6-phosphate, G16P glucose-1,6-bisphosphate, F1P fructose-1-phosphate, F6P fructose-6-phosphate, F16P fructose-1,6-bisphosphate, R5P ribose-5-phosphate, Ru5P ribulose-5-phosphate, pNPP *p*-nitrophenylphosphate. **c** Aldolase activity of the *Hx*-FucA condensing DHAP and six aldehydes was examined by quantifying the residual DHAP after reactions with a coupling enzyme. The activities were calculated from *n* = 3 independent experiments. Error bars indicate standard deviations.

Four Urdpase1 recombinant proteins from *H. salinarum*, *H. xanaduensis*, *H. lacusprofundi*, and *H. turkmenica* (*Hs*-, *Hx*-, *Hl*-, and *Ht*-Urdpase1 encoded by VNG_1850G, Halxa_1684, Hlac_2318, and Htur_0567, respectively) were prepared using *E. coli*. *Hl*-Urdpase1 and *Ht*-Urdpase1 were obtained as soluble proteins, while the other two formed inclusion bodies. *Hl*-Urdpase1 recombinant protein was purified to apparent homogeneity (Supplementary Fig. 2c). Nucleoside phosphorylase activity of purified *Hl*-Urdpase1 was examined toward six nucleosides, adenosine, inosine, guanosine, cytidine, uridine, and thymidine (Fig. 4a and Supplementary Fig. 7). *Hl*-Urdpase1 exhibited highest activity toward guanosine, and could recognize adenosine and inosine to a lower extent. Surprisingly, activity was lower at higher KCl concentrations (Supplementary Fig. 8a). The enzyme however retained considerable guanosine phosphorylase activity even at 2–4 M KCl. The optimal reaction temperature and pH of the enzyme were 30 °C and 7.5, respectively (Supplementary Fig. 8b, c). Kinetic analyses toward guanosine and phosphate revealed that the kinetic parameters *V*_max_ and *K*_m_ were 1.5 ± 0.1 μmol min^−1^ mg^−1^ and 56 ± 10 μM toward guanosine (Supplementary Fig. 9a) and 2.1 ± 0.1 μmol min^−1^ mg^−1^ and 4.8 ± 0.8 mM toward phosphate (Supplementary Fig. 9b), respectively. The results suggest that Hlac_2318 encodes a guanosine phosphorylase. The identification of guanosine phosphorylase, ATP-R1PK, and R15P isomerase implied the presence of a metabolic pathway converting guanosine, phosphate, and ATP to RuBP, guanine, and ADP via R1P and R15P (Fig. 1b). Although the phosphate donor of R1P kinase is ATP, the metabolic route from guanosine to RuBP corresponds to that in the pentose bisphosphate pathway identified in *Thermococcus*^13^.

### Search for enzymes involved in RuBP metabolism

Other than R15P isomerase, Rubisco is the sole enzyme known to utilize RuBP as a substrate. The presence of a metabolic route generating RuBP in halophilic archaea without a Rubisco suggested the presence of unidentified enzymes involved in the conversion of RuBP. We took a comparative genomics approach, along with phylogenetic analysis, to identify enzymes that might be involved in this metabolism. We found that a gene annotated as fuculose-1-phosphate aldolase (*fucA*) is specifically present in 17 of the 19 halophilic archaea that harbor a standalone R15P isomerase. FucA homologs are widely distributed among the halophilic archaea and a relationship with the occurrence of R15P isomerase is not readily apparent. However, a phylogenetic analysis (Supplementary Fig. 10) reveals a group of FucA homologs that displays co-occurrence with standalone R15P isomerases (Table 1, VNG_1201G [seventh column], designated FucA) and can be distinguished from the others (Table 1, VNG_1201G [eleventh column], designated FucA*). There were only two organisms (*H. tiamatea* and *Halohasta litchfieldiae*) that harbored a standalone R15P isomerase but not FucA. On the other hand, we found that another gene, annotated as haloacid dehalogenase (HAD)-superfamily hydrolase (HAD hydrolase, *hadhlase*), is also specifically present in the 17 halophiles (Table 1, VNG_1202C column). In addition, these two genes form an operon (Fig. 3) in all 17 halophilic archaea. Furthermore, in *H. lacusprofundi*, *Halorubrum* sp. PV6, and *Halorubrum ezzemoulense*, the four genes encoding ATP-R1PK, R15P isomerase, HAD hydrolase, and FucA form an operon. The two genes *fucA* and *hadhlase* display a stark co-occurrence with the three genes encoding guanosine phosphorylase, ATP-R1PK, and R15P isomerase, strongly raising the possibilities that these five proteins are metabolically related.

### Identification of an RuBP phosphatase

We examined the possibility that the proteins annotated as HAD hydrolase and FucA are involved in RuBP metabolism. The HAD superfamily hydrolase from *Arabidopsis thaliana* is known to catalyze the phosphatase reaction of ribose 5-phosphate (R5P) to ribose (Supplementary Fig. 11a)^34^. Based on the structural similarity between R5P and RuBP (Supplementary Fig. 11a, b), we hypothesized that the enzyme catalyzes an RuBP phosphatase reaction. The gene encoding HAD hydrolase from *H. xanaduensis* (Halxa_2271) was overexpressed in *E. coli* and the recombinant protein (*Hx*-HAD hydrolase) was purified to apparent homogeneity (Supplementary Fig. 2d). Phosphatase activity was measured toward 12 sugar phosphates. We observed notable phosphate production only when RuBP was used as a substrate (Fig. 4b), suggesting that the enzyme was a phosphatase with strict substrate specificity toward RuBP. The optimal KCl concentration was higher than 2.5 M (Supplementary Fig. 12a), while the optimal pH of the reaction was ~6 (Supplementary Fig. 12b). The cation specificity of the enzyme was broad, with similar levels of activity detected in the presence of 1 mM Mg^2+^, Mn^2+^, Co^2+^, and Ni^2+^ (Supplementary Fig. 12c). Kinetic analysis toward RuBP (Supplementary Fig. 13) revealed that *V*_max_ and *K*_m_ toward RuBP were 31 ± 2 µmol min^−1^ mg^−1^ and 2.2 ± 0.5 mM, respectively. The results implied that the enzyme, which had been annotated as HAD hydrolase (encoded by Halxa_2271), is an enzyme that catalyzes a previously unknown biological reaction, the dephosphorylation of RuBP. We designate the enzyme as RuBP phosphatase.

Dephosphorylation of RuBP can result in the generation of three reaction products, ribulose-1-phosphate (Ru1P), ribulose-5-phosphate (Ru5P), and ribulose. We thus carried out an ^1^H-NMR analysis of the reaction product (Supplementary Fig. 14). Although chemical shifts derived from RuBP were also detected, the chemical shifts specific to a cyclized Ru1P shown in a previous report^35^ were confirmed in the reaction product (Supplementary Fig. 14b, c). This suggested that RuBP phosphatase catalyzes the hydrolysis of the phosphate group at the C5-position of RuBP, generating Ru1P (Fig. 1b).

### Identification of an Ru1P aldolase

Classical FucA is an enzyme catalyzing the aldolase reaction of fuculose-1-phosphate (Fu1P). Fu1P is cleaved into dihydroxyacetone phosphate (DHAP) and l-lactaldehyde (Supplementary Fig. 11c). We noted that the chemical structures of Ru1P and Fu1P are similar and the difference lies only in the C6 methyl group (Supplementary Fig. 11b, c). In addition, we took note of the fact that while only at 10% of the levels of activity towards the physiological substrates DHAP and l-lactaldehyde, the fuculose-1-phosphate aldolase from *E. coli*, which displays 33.8% identity to FucA from *H. xanaduensis* (*Hx*-FucA), could recognize DHAP and glycolaldehyde^36^. Recombinant *Hx*-FucA was thus prepared using *E. coli* and purified to apparent homogeneity (Supplementary Fig. 2e). As Ru1P is not commercially available, we examined whether *Hx*-FucA can catalyze the aldolase reaction condensing DHAP and aldehydes by quantifying the residual DHAP. The aldehydes tested were acetaldehyde, propionaldehyde, isobutylaldehyde, dl-glyceraldehyde, dl-lactaldehyde, and glycolaldehyde (Supplementary Fig. 15). The protein displayed aldolase activity toward DHAP and all tested aldehydes (Fig. 4c). The result implied that although the substrate specificity of the enzyme was broad, it could catalyze the aldolase reaction cleaving Ru1P to DHAP and glycolaldehyde. With HPLC, we further confirmed that the reaction product of the *Hx*-FucA reaction with DHAP and glycolaldehyde displayed an elution time identical to that of the RuBP phosphatase reaction product (Supplementary Fig. 16a). An increase in activity was observed with the addition of ZnCl_2_ to the reaction mixture, while a decrease was observed with addition of EDTA, suggesting that the enzyme was dependent on zinc cations (Supplementary Fig. 17). In addition, we examined whether *Hx*-RuBP phosphatase and the *Hx*-FucA protein together could generate DHAP from RuBP. Only when both proteins were present in the reaction mixture, DHAP was produced from RuBP (Supplementary Fig. 16b). Furthermore, kinetic analyses of the enzyme revealed that *V*_max_ and *K*_m_ toward glycolaldehyde were 2.0 ± 0.0 µmol min^−1^ mg^−1^ and 3.4 ± 0.3 mM, respectively (Supplementary Fig. 18a), and those toward DHAP were 2.0 ± 0.0 µmol min^−1^ mg^−1^ and 3.5 ± 0.3 mM, respectively (Supplementary Fig. 18b). Based on these results, we conclude that the protein that had been annotated as FucA, encoded by Halxa_2272, is an Ru1P aldolase recognizing DHAP and various aldehydes including glycolaldehyde.

Based on the results obtained here, we propose that the standalone R15P isomerase is a component of a previously unidentified nucleoside metabolic pathway converting the ribose moiety of guanosine to glycolaldehyde and DHAP (Fig. 1b). As the pathway does not involve Rubisco, we here designate this pathway the non-carboxylating pentose bisphosphate pathway.

### Identification of a glycolaldehyde reductase

Although DHAP can be metabolized by central sugar metabolism, the fate of glycolaldehyde was still unclear. However, we could not identify a candidate enzyme based on genome information that would metabolize glycolaldehyde. Upon measuring enzyme activity in the *H. salinarum* cell-free extract that could potentially convert glycolaldehyde, we were able to detect reducing activity on glycolaldehyde using NADH as the electron donor.

From the cell-free extract of *H. salinarum* cultured with nucleosides, we purified the protein displaying glycolaldehyde reductase activity to apparent homogeneity (Supplementary Fig. 2f). By liquid chromatography–mass spectrometry (LC-MS) analysis, the purified protein was shown to be the product of the VNG_6270G gene, annotated as *sn*-glycerol-1-phosphate dehydrogenase. Enzymatic analysis revealed that the optimal KCl concentration was higher than 4 M, and the optimal reaction temperature and pH were 40 °C, and ~6, respectively (Supplementary Fig. 19). Kinetic analysis toward glycolaldehyde (Supplementary Fig. 20a) showed that the enzyme was subject to substrate inhibition. The *V*_max_, *K*_s1_, and *K*_s2_ values were 33 ± 4 μmol min^−1^ mg^−1^, 10 ± 2 mM, and 35 ± 8 mM, respectively. Taking into account the original annotation, we further examined whether the protein catalyzes the *sn*-glycerol-1-phosphate dehydrogenase reaction or not (Supplementary Fig. 20b). The purified enzyme did not display notable levels of activity for the oxidation of glycerol-1-phosphate nor the reduction of DHAP. In addition, the recombinant VNG_6270G protein was prepared using *E. coli*, and partially purified (Supplementary Fig. 3b). As expected, the recombinant protein (*Hs*-GaR) displayed high levels of glycolaldehyde reductase activity (40.7 ± 0.4 μmol min^−1^ mg^−1^).

As *sn*-glycerol-1-phosphate dehydrogenase is presumed to contribute to the biosynthesis of archaeal membrane lipid precursors by reducing DHAP to glycerol-1-phosphate, the enzyme would seem to be essential in all archaea. However, we found another gene annotated as *sn*-glycerol-1-phosphate dehydrogenase distributed in all halophilic archaea including *H. salinarum* (VNG_0406C) (Table 1, VNG_0406C column). The characterized archaeal glycerol-1-phosphate dehydrogenase from *Methanothermobacter thermautotrophicus*^37–39^ displays higher similarity with VNG_0406C protein (50.7% identical) than VNG_6270G protein (22.5% identical). This suggests that the VNG_6270G gene encodes glycolaldehyde reductase and not *sn*-glycerol-1-phosphate dehydrogenase (Fig. 1b), and that the gene encoding the *bona fide sn*-glycerol-1-phosphate dehydrogenase is most likely VNG_0406C.

Concerning the distribution of the VNG_6270G gene homologs (Table 1, VNG_6270G column), their distribution is limited to only ten halophilic archaea and the homolog in *H. salinarum* is encoded on a plasmid. Although half possess a complete set of genes forming the non-carboxylating pentose bisphosphate pathway, the other half does not. Although we could not exclude the possibility that there are plasmids whose sequences have been overlooked in the genome analyses, the contribution of VNG_6270G homologs to nucleoside degradation in halophilic archaea may be limited, and other routes for glycolaldehyde metabolism may exist.

### Enzyme activities in the cell-free extract from *H. salinarum*

The biochemical analyses described above involves enzymes from different species of halophilic archaea. In order to confirm that the proposed pathway is present in a single species, we examined the enzyme activities in the cell-free extract of *H. salinarum*. When each substrate was added to the reaction mixture including cell-free extracts, the products of guanosine phosphorylase (R1P), ATP-dependent R1P kinase (R15P), RuBP phosphatase (Ru1P), and glycolaldehyde reductase (ethylene glycol) reactions were detected (Supplementary Fig. 21a, b, d, f). On the other hand, when R15P was added to the reaction mixture, we could not detect RuBP, the product of R15P isomerase. However, instead of RuBP, we could observe the generation of Ru1P (Supplementary Fig. 21c). This result implied that the RuBP generated from R15P by R15P isomerase was subsequently converted to Ru1P by RuBP phosphatase. To confirm Ru1P aldolase activity, as Ru1P is not commercially available, a coupling reaction catalyzed by RuBP phosphatase and Ru1P aldolase was examined. As a result, DHAP, which is presumed to be produced by Ru1P aldolase from Ru1P, was clearly detected when RuBP and ZnCl_2_ were added (Supplementary Fig. 21e). The predicted activities of the enzymes encoded by the six genes were detectable in *H. salinarum*, suggesting the presence of the non-carboxylating pentose bisphosphate pathway in this organism.

## Discussion

Based on the results of this study, we propose a previously unrecognized nucleoside degradation pathway, the non-carboxylating pentose bisphosphate pathway, in halophilic archaea (Fig. 1b). Although the metabolism from guanosine to RuBP via R15P is similar to that in the pentose bisphosphate pathway in Thermococcales, the downstream route is unique. We can assume that the physiological role of the pathway is to convert the ribose moiety of nucleoside(s) to DHAP and ethylene glycol. DHAP can be utilized in various metabolisms, including oxidation to pyruvate via glyceraldehyde 3-phosphate, gluconeogenesis, and conversion to glycerol for utilization in membrane lipid biosynthesis and osmolyte production. On the other hand, the metabolic fate of ethylene glycol is still unclear and further examination will be necessary to understand if and how the cells utilize the two carbons deriving from pentoses.

**Fig. 5:**
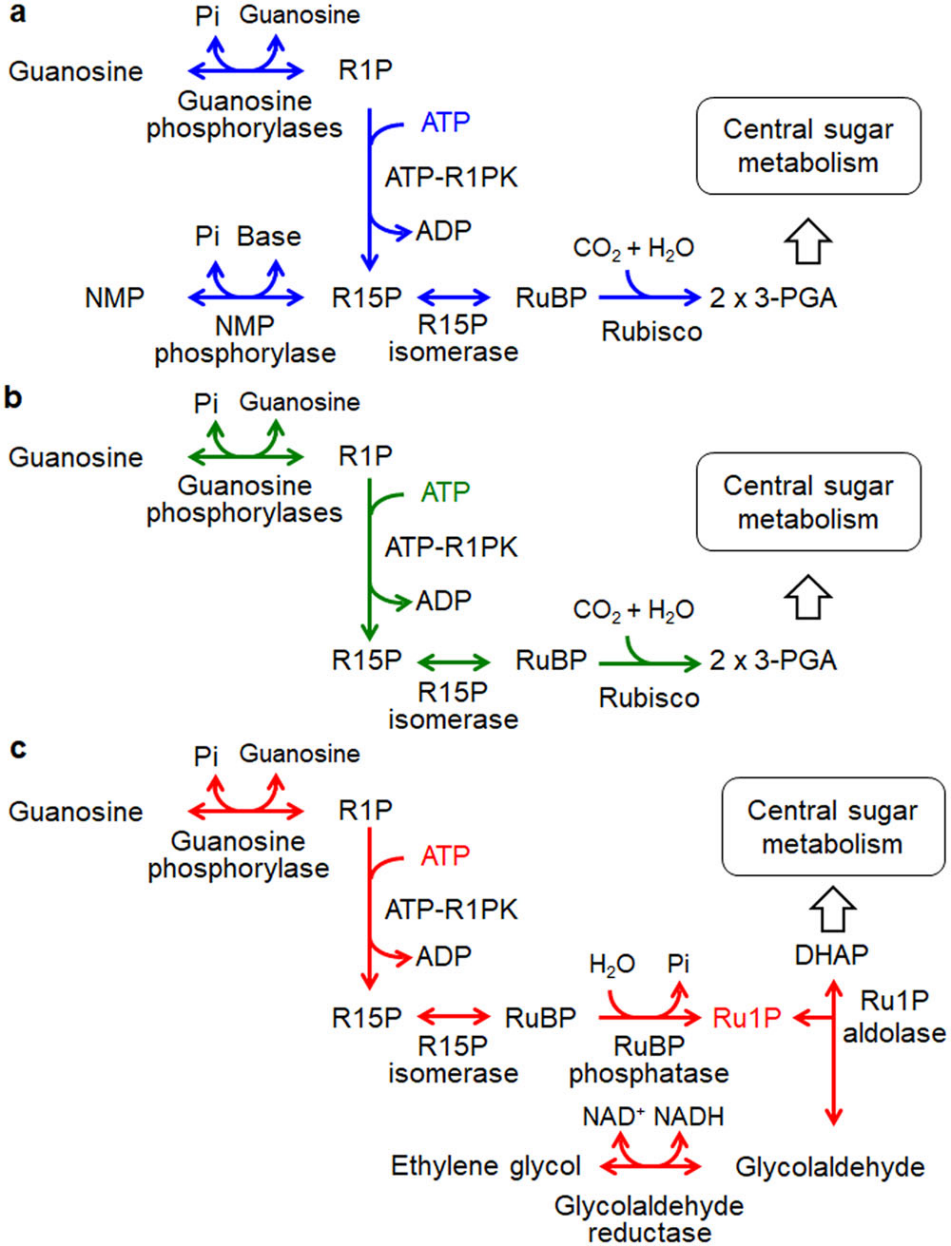
Nucleoside degradation networks in halophilic archaea. The results obtained in this study and the distribution of relevant genes imply the occurrence of three types of nucleoside degradation pathways in halophilic archaea. The three metabolic routes are with NMP phosphorylase and Rubisco (**a**), with Rubisco but without NMP phosphorylase (**b**), without Rubisco or NMP phosphorylase but with RuBP phosphatase and Ru1P aldolase (**c**).

**Fig. 6:**
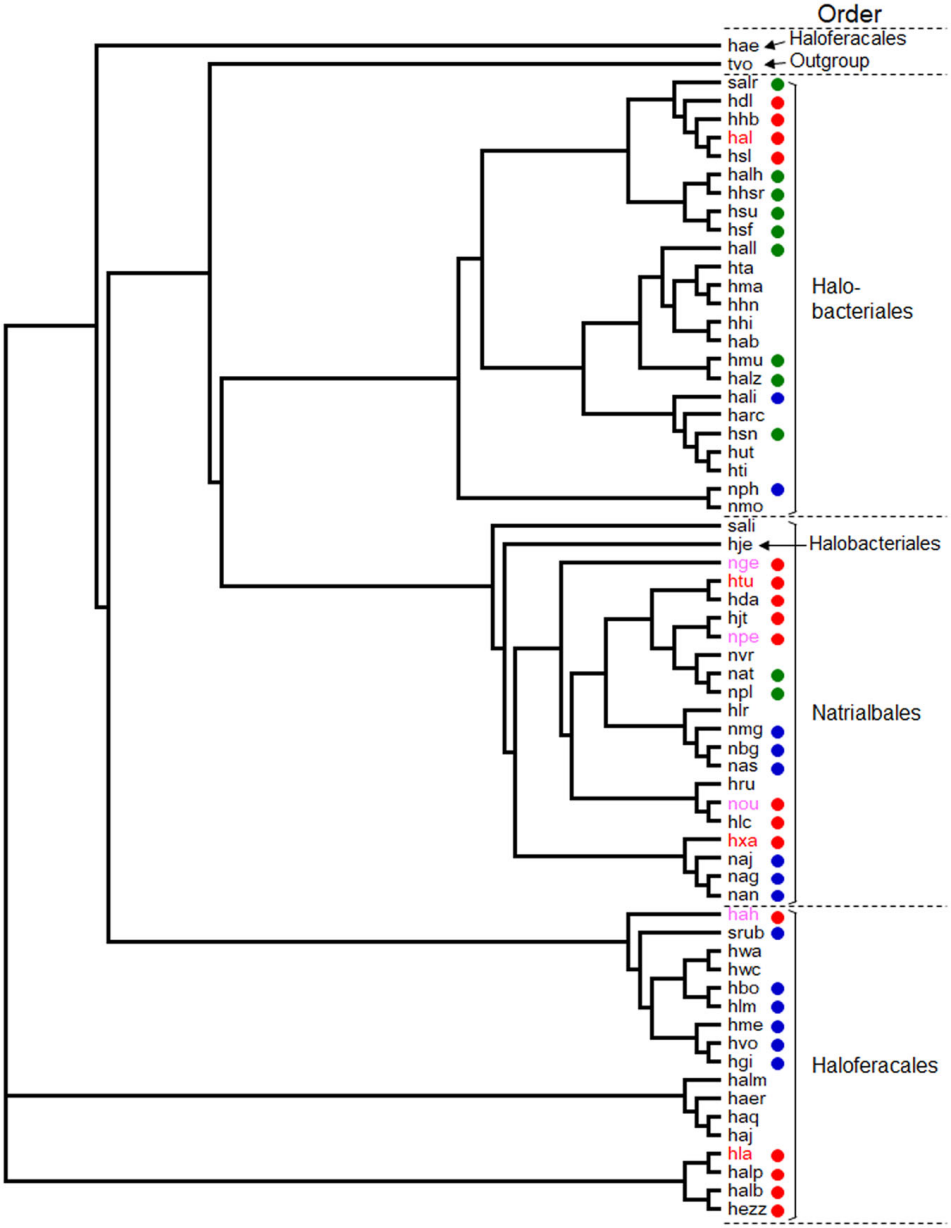
Relationship between the presence of the three nucleoside metabolic pathways and phylogenetic positions of halophilic archaea. The phylogenetic tree was constructed using 16S rRNA gene sequences. A sequence from *Thermoplasma volcanium* (tvo) was used as an outgroup. The colors of the circles correspond to those of the nucleoside metabolic pathways in Fig. 5. Organisms shown in red and pink codes indicate the halophilic archaea shown in Fig. 3. The organisms in red codes show those whose proteins were actually examined in this study.

Our results and the distribution of gene homologs suggest the presence of multiple variations of nucleoside degradation pathways in halophilic archaea, all involving the pentose bisphosphates R15P and RuBP. One common feature in the nucleoside degradation pathways found in halophilic archaea is that the ADP-R1PK found in Thermococcales is replaced by the ATP-R1PK identified in this study (Figs. 1 and 5). As shown in Table 1, among the 63 species of halophilic archaea whose genome sequences have been determined, 44 species harbor R15P isomerase homologs on their genomes, suggesting the presence of metabolism involving the pentose bisphosphates R15P and RuBP in these organisms. Among these, 25 species seem to utilize Rubisco for the metabolism of RuBP. The non-carboxylating pentose bisphosphate pathway identified in this study, utilizing RuBP phosphatase and Ru1P aldolase, is found in 17 halophile species and is also widely distributed. The remaining two species with an R15P isomerase do not harbor Rubisco nor RuBP phosphatase/Ru1P aldolase. As for NMP phosphorylase, homologs are only found in species with a Rubisco. Among the 25 species with Rubisco, 14 harbor an NMP phosphorylase homolog, while 11 do not. It thus seems that there are three major variations of the pentose bisphosphate pathway in halophiles that account for 42 of the 63 species shown in Table 1; (i) one with Rubisco and NMP phosphorylase, as seen in members of Thermococcales (Fig. 5a), (ii) one with Rubisco but without NMP phosphorylase (Fig. 5b), and (iii) the non-carboxylating pentose bisphosphate pathway identified in this study (Figs. 1b and 5c). The distribution of these variations among the halophilic archaea is not linked to the phylogenetic relationships of their source organisms (Fig. 6). Interestingly, there are still 10 species that harbor a set of homologs corresponding to nucleoside phosphorylase and ATP-R1PK (Table 1, yellow in VNG_1851G/VNG_1850G columns), that would convert the ribose moieties of nucleosides to R15P. This raises the possibility that there may be even more variations of nucleoside degradation pathways in halophilic archaea that involve pentose bisphosphates.

As indicated above, there are 21 halophile species whose genome sequences suggest an absence of the carboxylating/non-carboxylating pentose bisphosphate pathways. Among them, *Halorhabdus utahensis* and *Halorhabdus tiamatea* harbor homologs of the classical NOPP pathway found in bacteria and eukaryotes, and the NOPP pathway may be responsible for nucleoside degradation in these species. However, there are still 19 halophilic archaea whose genome sequences do not provide any clues as to how they carry out nucleoside degradation. Although we cannot rule out the possibility that these species do not utilize nucleosides, we have taken notice of a FucA* homolog distinct to that identified as the Ru1P aldolase in this study (Table 1, last column). The homolog tends to occur in these halophiles, including 9 species in *Haloarcula*, *Haloplanus*, and *Halohasta*, and might provide clues to identify additional pathways involved in nucleoside or pentose metabolism.

Analysis of recombinant *Hl*-Urdpase1 indicated that the enzyme preferred guanosine as the nucleoside substrate for the phosphorylase reaction. Interestingly, the enzyme (Hlac_2318) displays higher similarity with uridine phosphorylase (TK1479) (40.2% identical) rather than guanosine phosphorylase (TK1482) (18.7% identical) in *T. kodakarensis*. In addition to the guanosine phosphorylase, almost all halophilic archaea harbor a second uridine phosphorylase homolog, Urdpase2. Although these second homologs do not form an operon with genes of the pentose bisphosphate pathway, there is a high possibility that the gene products also generate R1P via nucleoside phosphorolysis. Phylogenetic analysis clearly revealed that Urdpase1 and Urdpase2 are distinct (Supplementary Fig. 6), suggesting that enzymatic characteristics of members from the two subgroups differ from each other. Identifying the nucleoside substrate of Urdpase2 will add to our understanding of nucleoside degradation in halophilic archaea. It is intriguing that only one of the two nucleoside phosphorylase genes, the guanosine phosphorylase gene, forms an operon with the genes of the non-carboxylating pentose bisphosphate pathway. This may be related to the high GC contents in genomes of halophilic archaea such as *H. salinarum* (67.9%)^30^. Nucleosides/nucleotides with guanine and cytosine might be maintained at a higher concentration within these cells.

Two R1P kinases have been identified in previous studies; an ADP-dependent R1P kinase from the hyperthermophilic archaeon *T. kodakarensis* (TK2029) and an ATP-dependent R1P kinase from the hyperthermophilic archaeon *Pyrobaculum calidifontis* (Pcal_0041). The ATP-dependent R1P kinase identified in this study (Halxa_1682) was 25% identical with the TK2029 protein and 36.8% identical to the Pcal_0041 protein. When comparing the properties of the three enzymes, the R1P kinase from *T. kodakarensis* is ADP-dependent, whereas the enzymes from *P. calidifontis* and *H. xanaduensis*/*H. salinarum* are ATP-dependent. On the other hand, the enzymes from *T. kodakarensis* and *H. xanaduensis* display strict substrate specificity toward R1P, while the R1P kinase from *P. calidifontis* can recognize cytidine and uridine in addition to R1P^29^. Neither R15P isomerase nor NMP phosphorylase gene homologs are present in *P. calidifontis*, resembling the case of the ten halophilic archaea with only nucleoside phosphorylase and ATP-R1PK homologs described above. On the other hand, the presence of R1P kinase in *E. coli* has been suggested^40^. An examination of enzymes/genes that can suppress a deletion in the 5-phospho-D-ribosyl-1-diphosphate (PRPP) synthase gene led to a proposed pathway with the reaction sequence R5P → R1P → R15P → PRPP. A protein encoded by *phnN* was identified that displays kinase activity towards R15P, leading to the production of PRPP. A protein responsible for the proposed second reaction, although unidentified, would be an R1P kinase. *E. coli* possesses several enzymes displaying similarity with the archaeal proteins that display R1P kinase activity. As these include proteins whose functions have not been determined, one of them may also be R1P kinase. R1P kinase and its product, R15P, may be distributed through Archaea and Bacteria more widely than previously expected.

## Methods

### Chemicals, strains, and media

Unless mentioned otherwise, chemical reagents were purchased from Nacalai Tesque (Kyoto, Japan), FUJIFILM Wako Pure Chemicals (Osaka, Japan), or Merck (Darmstadt, Germany). Strains and plasmids used in this study are listed in Supplementary Table 2. The halophilic archaeon *Halobacterium salinarum* and its genomic DNA were provided by the RIKEN BioResource Research Center through the National BioResource Project of MEXT/AMED, Japan. *H. salinarum* was cultivated at 40 °C in a medium containing 250 g l^−1^ sodium chloride, 20 g l^−1^ MgSO_4_ · 7H_2_O, 3 g l^−1^ trisodium citrate, 2 g l^−1^ potassium chloride, 5 g l^−1^ tryptone, and 3 g l^−1^ yeast extract (defined as high-salt medium in this study). When necessary, nucleosides were added into the medium. *Escherichia coli* DH5α strain used for plasmid construction and *E. coli* BL21 CodonPlus(DE3)-RIL and Rosetta (DE3) strains used for gene expression were cultured at 37 °C in lysogeny broth (LB) medium containing ampicillin (100 mg l^−1^).

### Construction of gene expression plasmids

Plasmids to express genes encoding *Hs*-R15P isomerase and *Hs*-Urdpase1 were constructed as follows. Coding regions of *Hs*-R15P isomerase and *Hs*-Urdpase1 genes were amplified by PCR using genomic DNA from *H. salinarum* as a template and expHs-R15Pi-F/-R and Hs-Urdpase1-F/-R as primer sets, respectively. The primers were designed so that NdeI and BamHI restriction sites were introduced at the 5’- and 3’-termini of the amplified DNA fragments, respectively. Sequences of these primers are listed in Supplementary Table 3. After digestion with NdeI and BamHI, the DNA fragments were individually ligated with pET-21a(+) digested with same restriction enzymes. The resulting expression plasmids are designated pET-Hs-R15P isomerase and pET-Hs-Urdpase1 (Supplementary Table 2).

Expression plasmids for genes encoding *Ht*-R15P isomerase, *Hs*-RbsK, *Ht*-RbsK, *Hx*-RbsK, *Ht*-Urdpase1, *Hx*-Urdpase1, *Hl*-Urdpase1, *Hx*-HAD hydrolase, *Hx*-FucA, and *Hs*-GaR (Supplementary Table 2) were prepared as follows. Genes were designed and synthesized (Integrated DNA Technologies, Coralville, IA) to decrease their GC contents, and optimize their codons to enhance gene expression in *E. coli*. The designed genes with NdeI and BamHI sites at 5’- and 3’-termini, respectively, were digested with NdeI and BamHI and ligated with pET-21a(+) digested with same restriction enzymes. For all 10 resulting expression plasmids (Supplementary Table 2) with the two plasmids described above, we carried out DNA sequencing analysis and confirmed the absence of unintended mutation. The designed sequences of each gene are shown in Supplementary Fig. 22.

### Heterologous gene expression in *E. coli*

The constructed expression plasmids were introduced into *E. coli* Rosetta (DE3) (for genes encoding R15P isomerase, RbsK, HAD hydrolase, and FucA) or *E. coli* BL21-CodonPlus(DE3)-RIL (for genes encoding Urdpase1 and GaR). Transformants were cultured at 37 °C until their OD_660_ reached 0.4–0.8, and gene expression was induced by the addition of isopropyl-β-d-thiogalactopyranoside (IPTG) at a final concentration of 0.1 mM. After further cultivation at 37 °C for 4 h, cells were harvested.

### Purification of recombinant proteins

Cells expressing *Hs*-GaR, *Ht*-R15P isomerase, *Hx*-RbsK, and *Hx*-FucA recombinant proteins were resuspended with 50 mM Tris-HCl (pH 7.5) and disrupted by sonication. After centrifugation (5000 x *g*, 15 min, 4 °C), the supernatant was applied to an anion-exchange column (Resource Q, GE Healthcare, Little Chalfont, Buckinghamshire, UK) equilibrated with 50 mM Tris-HCl (pH 7.5). Proteins were eluted with a linear gradient of NaCl (0 to 1.0 M) in 50 mM Tris-HCl (pH 7.5). For the three proteins other than GaR, the buffers of relevant fractions were changed to sodium phosphate buffer with 2 M NaCl and applied to a ceramic hydroxyapatite affinity column (Bio-Scale CHT 10-1, Bio-Rad, Hercules, CA) equilibrated with 7 mM sodium phosphate (pH 7.5) including 2 M NaCl. The proteins were eluted with a linear gradient of sodium phosphate (7 to 280 mM) in 2 M NaCl. Relevant fractions were collected and further purified with a gel filtration column Superdex200 (GE Healthcare) equilibrated with 50 mM Tris-HCl (pH 7.5) including 2 M NaCl. The same buffer was used as mobile phase. For *Hs*-GaR, relevant fractions after anion-exchange chromatography were applied to a gel filtration column equilibrated with 50 mM Tris-HCl (pH 7.5) including 2 M KCl.

*Hl*-Urdpase1 and *Hx*-HAD hydrolase recombinant proteins were purified with Bio-Scale CHT 10-1 and Superdex200. Cells producing *Hl*-Urdpase1 and *Hx*-HAD hydrolase proteins were resuspended in 5 mM and 7 mM sodium phosphate (pH 7.5) with 2 M NaCl, respectively, and disrupted by sonication. After centrifugation, the supernatants were applied to Bio-Scale CHT 10-1 equilibrated with the same buffers used for cell suspension. *Hl*-Urdpase1 and *Hx*-HAD hydrolase were eluted with linear gradients of sodium phosphate (5 to 500 mM and 7 to 280 mM, respectively) in 2 M NaCl. *Hl*-Urdpase1 and *Hx*-HAD hydrolase were further purified with a Superdex200 column equilibrated with 50 mM sodium phosphate (pH 7.5) with 2 M KCl and 50 mM Tris-HCl (pH 7.5) with 2 M KCl, respectively, with the buffers used as mobile phases.

The concentrations of the purified enzymes were determined with the Protein Assay System (Bio-Rad), using bovine serum albumin (BSA) (Thermo Fisher Scientific, Waltham, MA) as a standard. Protein homogeneity was confirmed by sodium dodecyl sulfate-polyacrylamide gel electrophoresis (SDS-PAGE).

### Purification of the GaR protein from the cell-free extract of *H. salinarum*

*H. salinarum* was cultured in 200 ml of high-salt medium supplemented with four nucleosides (adenosine, uridine, guanosine, and cytidine: 0.5 mM each). After cell density (OD_660_) reached 0.7, cells were harvested, resuspended with 5 ml of 5 mM sodium phosphate (pH 7.5) with 2 M KCl, and disrupted by sonication. After centrifugation (5000 x *g*, 15 min, 4 °C), the supernatant was applied to a Bio-Scale CHT 10-1 column equilibrated with 5 mM sodium phosphate (pH 7.5) with 2 M KCl. Proteins were eluted with a linear gradient of sodium phosphate (5 to 500 mM) in 2 M KCl. Fractions displaying glycolaldehyde reductase activity were collected. Further purification was performed with a Superdex200 column equilibrated with 50 mM Tris-HCl (pH 7.5) including 2 M NaCl. The same buffer was used as a mobile phase. Protein concentration was determined as described above.

### Identification of an enzyme displaying glycolaldehyde reductase activity by LC-MS

The amino acid sequence of the purified protein exhibiting glycolaldehyde reductase activity was identified by LC-MS analysis. After separation with SDS-PAGE, proteins were stained with silver staining. The portion of the gel containing the target protein was excised and destained with Silver Stain MS Kit. The protein in the gel was reduced, alkylated, digested with trypsin, and extracted using In-Gel Tryptic Digestion Kit (Thermo Fisher Scientific). The trypsin-digested peptides were analyzed by nano-flow reverse phase liquid chromatography followed by tandem MS, using an LTQ-Orbitrap XL hybrid mass spectrometer (Thermo Fisher Scientific). Samples were automatically injected using PAL system (CTC analytics, Zwingen, Switzerland) into a peptide L-trap column OSD (5 µm; AMR, Tokyo, Japan) attached to an injector valve for desalinating and concentrating peptides. After washing the trap with MS-grade water containing 0.1% TFA acid and 2% acetonitrile (solvent C), peptides were loaded into a nano HPLC capillary column (C18-packed with the gel particle size of 3 µm, 0.1 × 150 mm; Nikkyo Technos, Tokyo, Japan). The eluents were: A, 100% water containing 0.5% acetate, and B, 80% acetonitrile containing 0.5% acetate. In the column the concentration gradient of acetonitrile was increased: from 5% B to 45% B in 60 min, 45% B to 95% B in 1 min, sustaining 95% B for 19 min, from 95% B to 5% B in 1 min, and finally re-equilibrating with 5% B for 9 min. The flow rate was accelerated from 200 to 500 nl min^−1^ with a concentration gradient of acetonitrile. Xcalibur 2.1 system (Thermo Fisher Scientific) was used to analyze peptide spectra within the mass range of *m/z* 350–1500 and analyze the MS/MS spectra in information-dependent data acquisition within the mass range of *m/z* 150–2000. Fragmentation was performed by collision induced dissociation (CID). Multiple charged peptides with good fragmentation characteristics were chosen for MS/MS experiments. MS/MS spectra were interpreted and peak lists were prepared with Proteome Discoverer 1.4 and 2.0 (Thermo Fisher Scientific). Gene searches were carried out by using the SEQUEST-HT (Thermo Fisher Scientific).

### Measurement of R15P isomerase activity of the *Ht*-R15P isomerase recombinant protein

R15P isomerase activity was examined using ribose-1,5-bisphosphate (R15P) as the substrate and detecting the product RuBP with HPLC. The reaction mixture (100 μl) was composed of 50 mM Tris-HCl (pH 8.0), 5 mM R15P (Tokyo Chemical Industry, Tokyo, Japan), 10 mM MgCl_2_, 1.84 M KCl, and 2 or 3 μg of the purified recombinant *Ht*-R15P isomerase protein. The reaction mixture without R15P was pre-incubated at 47 °C for 3 min. The reaction was initiated by the addition of R15P, carried out at 47 °C for 15 min, and stopped by rapid cooling on ice and removing the enzyme by ultrafiltration with Amicon Ultra-0.5 Centrifugal Filter Unit (MWCO: 10 K) (EMD Millipore, Billerica, MA). The reaction product was analyzed by HPLC using an Asahipak NH2P-50 4E column (Shodex, Tokyo, Japan) with 300 mM sodium phosphate (pH 4.4) as the mobile phase. The eluted compounds were monitored with a differential refractive index detector. HPLC chromatogram data measured with LC-10A or Nexera X2 systems (Shimadzu, Kyoto, Japan) were collected using LCsolution 1.22 SP1 or LabSolutions 5.57.

### Measurement of kinase activity of the *Hx*-RbsK recombinant protein

Kinase activity of *Hx*-RbsK was measured by quantifying the amount of NDPs produced from NTPs with coupling enzymes, pyruvate kinase and lactate dehydrogenase (PK/LDH). PK converts phosphoenolpyruvate and NDPs into pyruvate and NTPs. LDH catalyzes the conversion of pyruvate and NADH into lactate and NAD^+^. The generated NDPs were calculated by measuring the decrease in absorbance at 340 nm (A_340_) derived from NADH.

To measure activity of *Hx*-RbsK, NDP production was examined as follows. The kinase reaction mixture (100 μl) contained a mixture of 50 mM Tris-HCl and 50 mM Bicine-NaOH (pH 8.3 or 8.4), 25 mM phosphate acceptors including ribose-1-phosphate (R1P) (Toronto Research Chemicals Inc., Toronto, Canada), NTP (4 mM ATP [ORIENTAL YEAST, Tokyo, Japan], 4 mM ATP with 2 mM each GTP/UTP/CTP, 2 mM each ATP/GTP/UTP/CTP/TTP, or 4 mM ATP with 1.5 mM each GTP/UTP/CTP/TTP), 20 mM MgCl_2_, 1.8 M or 4 M NaCl, and 2, 2.5, or 4 μg of partially purified *Hx*-RbsK protein. Eighty-five phosphate acceptors tested as substrates and reaction conditions are summarized in Supplementary Table 1. The reaction mixture without NTP was incubated at 42 or 47 °C for 3 min and the reaction was initiated by the addition of NTP and carried out for 15, 30, or 60 min. The reaction was stopped by rapid cooling on ice for 10 min and removing the enzyme by ultrafiltration as described above. The PK/LDH reaction mixture (100 μl) was composed of 15 or 50 mM MES-NaOH (pH 6.5), 25–50 μl of the kinase reaction mixture, 5 or 20 mM phosphoenolpyruvate (PEP), 0.2 mM NADH (ORIENTAL YEAST), 5 units of PK, and 7 units of LDH. A_340_ of the PK/LDH reaction mixture without coupling enzymes was measured. The coupling enzymes were added and *A*_340_ was monitored at room temperature until a decrease was no longer observed. Unless described otherwise, absorbance was measured with a spectrophotometer, Ultrospec 3000 (GE Healthcare) or Ultrospec 6300 *pro* (GE Healthcare). The applied molar absorbance coefficient of NADH was *ε*_340 nm_ = 6.22 × 10^3 ^M^−1^ cm^−1^. The reaction without phosphate acceptor substrate was carried out and the value was subtracted from the values obtained with the acceptor substrates. The amounts of produced NDPs were normalized by dividing with reaction time (min) and the amount of protein (mg).

When NTP specificity was examined, the reaction was performed at 42 °C for 5 min in the presence of 25 mM R1P, 4 mM NTPs, 0.8 M NaCl, 1.2 μg of purified protein, and other components described above. For the determination of specific activity toward R1P, the reaction was carried out at 47 °C for 3, 4, and 5 min with the reaction mixture including 20 mM R1P, 4 mM ATP, 2 M KCl, and other components described above. To determine KCl dependency, NaCl was replaced with KCl (0–3 M) and specific activities were measured at 47 °C in the presence of 15 mM R1P, 4 mM ATP, and other components described above. In the analysis of *Hx*-RbsK reaction product with HPLC, 10 mM R1P, 30 mM ATP, 2 M KCl, 1 μg of purified protein, and other components described above were included in the 100-μl reaction mixture. The reaction was carried out at 47 °C for 15 min. After rapid cooling on ice for 10 min and ultrafiltration to remove *Hx*-RbsK, the filtrate was analyzed with HPLC as described above. For the coupled reaction of *Hx*-RbsK and *Ht*-R15P isomerase, 50-μl aliquots of the filtrate were added into an *Ht*-R15P isomerase reaction mixture with 5 mM AMP and without R15P. After incubation at 47 °C for 3 min, the reaction was initiated and performed for 15 min. RuBP formation was detected by HPLC as described above.

### Measurement of nucleoside phosphorylase activity of the *Hl*-Urdpase1 recombinant protein

Nucleoside phosphorylase activity of *Hl*-Urdpase1 was measured by quantifying the released nucleobase with HPLC. Unless described otherwise, the reaction mixture (100 μl) included 50 mM sodium phosphate (pH 7.5), 2 mM nucleosides (adenosine, inosine, guanosine, uridine, cytidine, or thymidine), 2 M KCl, and 1 μg of purified enzyme. After incubation of the reaction mixture without nucleosides at 37 °C for 3 min, the reaction was started by adding nucleosides. After incubation at 37 °C for various periods of time, the reaction was stopped by rapid cooling on ice for 20 min and removing the enzyme by ultrafiltration as described above. For HPLC, an equal volume of a 20 mM sodium phosphate (pH 7.5), 20% methanol solution was added to the filtrate. The reaction products were analyzed by HPLC using a COSMOSIL 5C18-PAQ packed column (4.6 I. D. x 250 mm, Nacalai Tesque) equilibrated with 20 mM sodium phosphate (pH 7.5) with 10% methanol at a temperature of 40 °C. The released nucleobases were detected by UV absorbance at 260 nm. Adenosine, adenine, inosine, hypoxanthine, guanosine, guanine, uridine, uracil, cytidine, cytosine, thymidine, and thymine were utilized as standard compounds to prepare standard curves.

In determining general enzymatic properties, varying KCl concentrations (0–4 M), reaction temperatures (20–90 °C), and reaction pH (sodium phosphate buffer for pH 2.0–5.0, MES-NaOH buffer for pH 5.5–7.0, HEPES-NaOH buffer for pH 7.0–8.0, Bicine-NaOH buffer for pH 8.0–9.0) were tested. For kinetic analysis toward guanosine, various concentrations of guanosine (0–1000 μM) were tested at 30 °C in the presence of 50 mM sodium phosphate (pH 7.5) and 2 M KCl. Kinetic analysis toward phosphate was carried out with various concentrations of sodium phosphate (0–32 mM) in the presence of 1 mM guanosine at 30 °C with 2 M KCl. In all kinetic analyses of enzymes, curve fitting and calculation of *V*_max_ and *K*_m_ values were carried out with IGOR Pro version 6.03AJ.

### Measurement of phosphatase activity of the *Hx*-HAD hydrolase recombinant protein

Phosphatase activity of *Hx*-HAD hydrolase was determined by quantifying released phosphate with a malachite green assay. The reaction mixture (100 μl) included 50 mM Tris-HCl (pH 7.5), 5 mM sugar phosphates, 2 M KCl, 5 mM MgCl_2_, and 1 μg of the purified *Hx*-HAD hydrolase recombinant protein. Twelve substrates, glucose-1-phosphate (G1P), glucose-6-phosphate (G6P), glucose-1,6-bisphosphate (G16P), fructose-1-phosphate (F1P), fructose-6-phosphate (F6P), fructose-1,6-bisphosphate (F16P), ribose-5-phosphate (R5P), ribose-1-phosphate (R1P), ribose-1,5-bisphosphate (R15P), ribulose-5-phosphate (Ru5P), ribulose-1,5-bisphosphate (RuBP), and *p*-nitrophenylphosphate (pNPP), were examined. After incubation of the reaction mixture without substrates for 5 min at 37 °C, the reaction was initiated by the addition of the substrate. After 10 min incubation, the reaction was stopped by rapid cooling on ice and removing enzyme by ultrafiltration as described above. Released free phosphate was quantified using Malachite Green Phosphate Assay kits (BioAssay Systems, Hayward, CA). The reaction products were diluted appropriately to a final volume of 80 μl, and then mixed with 20 μl of the specified reagent. After 30 min incubation at room temperature, absorbance at 660 nm was measured with a spectrophotometer.

In determining general enzymatic properties, the reaction was carried out in a 100-μl reaction mixture containing 50 mM Tris-HCl (pH 7.5) or 50 mM MES (pH 6.1), 10 mM RuBP, 2.5 M KCl, 5 mM MgCl_2_, and 0.5 μg of the purified enzyme. Effects of salts were examined by varying the KCl concentration (1–3 M) or using NaCl (3 M). Effects of pH were examined with 50 mM of acetic acid (pH 4.4–5.9), MES (pH 6.1–7.4), or PIPES (pH 7.5–8.5). Effects of divalent metal cations were examined with 5 mM MgCl_2_, CaCl_2_, MnCl_2_, CoCl_2_, NiCl_2_, or EDTA. Kinetic analysis toward RuBP was performed in a 100-μl reaction mixture containing 50 mM Tris-HCl (pH 7.5), various concentrations of RuBP (0–20 mM), 2 M KCl, 5 mM MgCl_2_, and 0.5 μg of the purified enzyme. After 5 min pre-incubation, the reaction was started by adding RuBP. Reaction times were 1, 3, and 5 min for reactions with 0.1–0.5 mM RuBP and 1, 4, and 7 min for those with other concentrations.

### Measurement of aldolase activity of the *Hx*-FucA recombinant protein

Aldolase activities of *Hx*-FucA were measured with dihydroxyacetone phosphate (DHAP) and various aldehydes as substrates. Residual DHAP after the condensation reaction was quantified with glycerol-3-phosphate dehydrogenase (GPDH) by measuring NADH consumption. The aldolase reaction mixture (100 μl) contained 50 mM Tris-HCl (pH 8.0), 5 mM DHAP, 25 mM aldehydes (acetaldehyde, propionaldehyde, isobutylaldehyde, dl-glyceraldehyde, dl-lactaldehyde, or glycolaldehyde), 2 M KCl, 1 mM ZnCl_2_, and 4 μg of purified enzyme. After incubation of the reaction mixture without aldehydes at 47 °C for 3 min, the reaction was started by adding individual aldehydes. After further incubation at 47 °C (3–20 min), the reaction was stopped by rapid cooling on ice for 5 min and removing enzyme by ultrafiltration as described above. The coupling reaction mixture (100 μl) contained 50 mM Tris-HCl (pH 7.5), 5 μl of an appropriately diluted aliquot of the first reaction mixture, 0.2 mM NADH, and 1.7 units of GPDH. After the addition of GPDH, *A*_340_ was monitored with an Ultrospec 6300 *pro* until a decrease was no longer observed and residual DHAP was quantified based on the amount of consumed NADH.

The *Hx*-HAD hydrolase reaction coupled with the *Hx*-FucA reaction was examined by detecting DHAP production from RuBP. The reaction mixture (100 μl) contained 50 mM Tris-HCl (pH 7.5), 10 mM RuBP, 5 mM MgCl_2_, 2 M KCl, 1 mM ZnCl_2_, 1 μg each of *Hx*-HAD hydrolase and *Hx*-FucA. After incubation without RuBP at 47 °C for 3 min, the reaction was started by adding RuBP. After 3 h incubation, the reaction was stopped by rapid cooling on ice and removing enzyme by ultrafiltration. The reaction mixture (100 μl) to quantify DHAP contained 50 mM Tris-HCl (pH 7.5), 40 μl of the first reaction mixture, 0.3 mM NADH, and 1.7 units of GPDH. DHAP production was quantified as described above.

Kinetic parameters of *Hx*-FucA protein toward DHAP and glycolaldehyde were determined as follows. The reaction mixture (100 μl) included 50 mM Tris-HCl (pH 8.0), the substrates glycolaldehyde and DHAP, 2 M KCl, 1 mM ZnCl_2_, and 2 μg of purified *Hx*-FucA protein. Various concentrations of DHAP (1–20 mM) or glycolaldehyde (1–30 mM) were tested with a constant concentration of glycolaldehyde (50 mM) or DHAP (20 mM), respectively. After incubation at 47 °C for 3 min, the reaction was started by adding DHAP or glycolaldehyde. After 12, 16, and 20 min incubation, the reaction was stopped by rapid cooling on ice for 5 min and removing the proteins with ultrafiltration columns. The produced Ru1P was quantified by HPLC using an Asahipak NH2P-50 4E column and a UV detector (200 nm). The column was equilibrated with 300 mM sodium phosphate buffer (pH 4.4) at 40 °C and the same buffer was used as a mobile phase.

As Ru1P is not commercially available, the *Hx*-HAD hydrolase protein was utilized to produce Ru1P for constructing a standard curve of Ru1P. The reaction was carried out as follows. The reaction mixture (100 μl) included 50 mM MES-NaOH buffer (pH 6.0), 10 mM RuBP, 2 M KCl, 5 mM MgCl_2_, and 1 μg of *Hx*-HAD hydrolase protein. After incubation at 37 °C for 5 min without RuBP, the reaction was started by adding RuBP. After incubation at 37 °C for 5 h, the reaction was stopped by rapid cooling on ice for 5 min and removing the proteins with an ultrafiltration column. After confirming the complete consumption of RuBP by HPLC, the concentration of the generated Ru1P was presumed to be 10 mM and used to construct a standard curve for Ru1P.

### Measurement of reductase activity of the *Hs*-GaR protein

Reductase activities toward glycolaldehyde and DHAP were measured by monitoring NADH consumption (*A*_340_) in the reaction mixture with a UV spectrophotometer, UV-1800 (Shimadzu) and data was collected with UVProbe 2.52. The reaction mixture (1 ml) included 50 mM Tris-HCl (pH 7.5) or 50 mM/100 mM MES-NaOH (pH 6.0), 7.5 or 20 mM glycolaldehyde/7.5 mM DHAP, 0.2 mM NADH, 2 M KCl, and 0.2 or 2 μg of purified glycolaldehyde reductase. After the incubation of the reaction mixture without NADH or recombinant protein at 40 °C for 1 min, the reaction was started by adding NADH or purified recombinant protein, respectively. Data analysis was carried out with UVProbe 2.52 software.

Effects of KCl concentrations (0–4 M) in 50 mM MES-NaOH (pH 6.5) at 30 °C, reaction temperatures (20–90 °C) with 50 mM MES-NaOH (pH 6.5) and 4 M KCl, and pH values (MES-NaOH [pH 5.5–7.0], HEPES-NaOH [7.0–8.0], Bicine-NaOH [8.0–9.0] at 40 °C and in the presence of 4 M KCl, were tested. Kinetic parameters toward glycolaldehyde at 40 °C were determined in a reaction mixture (1 ml) containing 50 mM MES-NaOH (pH 6.0), various concentrations of glycolaldehyde (0–70 mM), 0.2 mM NADH, 3 M KCl, and 0.2 μg of the purified enzyme.

### Measurement of oxidase activity of the *Hs*-GaR protein

Oxidase activity toward *sn*-glycerol-1-phosphate was examined as follows. The reaction mixture (1 ml) included 50 mM MES-NaOH (pH 6.0), 7.5 mM *sn*-glycerol-1-phosphate, 0.2 mM NAD^+^, 2 M KCl, and 0.2 μg of purified glycolaldehyde reductase. After incubating the reaction mixture without NAD^+^ at 40 °C for 1 min, the reaction was started by adding NAD^+^ and A_340_ derived from NADH was measured with a UV-1800 to calculate DHAP production and data was collected with UVProve 2.52.

### Identification of the reaction product of *Hx*-HAD hydrolase

The reaction product of *Hx*-HAD hydrolase was analyzed by NMR. The reaction mixture (300 μl) contained 100 mM ammonium acetate (pH 6.65), 10 mM RuBP, 2 M KCl, 5 mM MgCl_2_, and 1 μg of the purified enzyme. After incubating the reaction mixture without RuBP for 5 min at 37 °C, the reaction was started by adding RuBP. After 10 min incubation, the reaction was stopped by removing enzyme with ultrafiltration column as described above. The reaction product was concentrated three times by vacuum drying, appropriately diluted with D_2_O (D, 99.96%) (Cambridge Isotope Laboratories Inc., Tewksbury, MA), and analyzed with ^1^H-NMR.

^1^H-NMR measurement was carried out using an ECA-600 spectrometer (JEOL, Tokyo, Japan) at 600 MHz and 5 °C and data was collected with Delta 4. The ^1^H-NMR spectra were acquired using a pulse sequence incorporating presaturation for water suppression. The presaturation power was 56 dB, which is the minimum required for complete suppression of the water peak. The chemical shifts of the ^1^H-NMR spectra were given in ppm relative to the signals of solvents using external standards of D_2_O at 4.62 ppm. The obtained NMR data was analyzed with Alice2 version 6.

### Measurement of enzyme activities in the cell-free extract from *H. salinarum*

*H. salinarum* was cultured in high-salt medium supplemented with 2 mM adenosine, 100 mM uridine, 2 mM guanosine, and 100 mM cytidine. When measuring guanosine phosphorylase activity, cells were cultured without nucleosides to decrease background signals deriving from the added nucleosides. Cell-free extracts were prepared as described for purifying GaR. To reduce background peaks, small molecules in the cell extract were reduced by diluting 8 times with 50 mM phosphate buffer (pH 7.5) by ultrafiltration. The reaction mixture (100 μl) contained 50 mM sodium phosphate (pH 7.5), 1500 μg of the cell-free extract, and substrate. Substrates, their concentrations, and reaction times/temperatures were 1 mM guanosine for guanosine phosphorylase (5 h/40 °C), 40 mM R1P with 20 mM ATP and 20 mM MgCl_2_ for R1P kinase (17 h/47 °C), 40 mM R15P for R15P isomerase (12 h/40 °C), 20 mM RuBP and 20 mM MgCl_2_ for RuBP phosphatase (15 min/40 °C), 100 mM RuBP with 20 mM MgCl_2_ and 1 mM ZnCl_2_ for Ru1P aldolase (12 h/40 °C), 40 mM glycolaldehyde and 30 mM NADH for glycolaldehyde reductase (1 h/40 °C). Incubation was carried out for 3 min prior to adding substrates; guanosine, ATP, R15P, RuBP, RuBP, and NADH, respectively. Reactions were stopped by rapid cooling on ice for 10 min and removing enzymes by ultrafiltration. Reaction products were analyzed by HPLC using a COSMOSIL 5C18-PAQ packed column and a mobile phase of H_2_O at 40 °C for glycolaldehyde reductase and an Asahipak NH2P-50 4E column and a mobile phase of 300 mM sodium phosphate (pH 4.4) at 40 °C for the other proteins. The products (R1P, R15P, RuBP, Ru1P, DHAP, and ethylene glycol) were monitored by a differential refractive index detector. Standard compounds were commercially available R1P (10 mM), R15P (10 mM), DHAP (5 mM), and ethylene glycol (15 mM), along with Ru1P enzymatically prepared from RuBP (5 mM) (described above).

### Homology search and phylogenetic analysis

Protein homology searches were carried out against the KEGG Genes database utilizing the BLAST Search program (https://www.genome.jp/tools/blast/). Sequences of 16S ribosomal RNA genes from halophiles were obtained from the KEGG Genes database. When there were multiple 16S ribosomal RNAs in one organism, one sequence was randomly selected. Phylogenetic analysis was performed using the ClustalW program (https://www.genome.jp/tools-bin/clustalw), while the phylogenetic tree was constructed using the TreeView version 1.6.6.

### Statistics and reproducibility

Reproducibility of the data was carried out by performing *n* = 3 independent experiments to collect data and determining and providing means and standard deviations.

### Reporting summary

Further information on research design is available in the Nature Research Reporting Summary linked to this article.

## Supplementary information

Supplementary_Information

Description of Additional Supplementary Data

Supplementary Data 1

Reporting Summary-New

## Data Availability

Gene locus_tag and organism code were adopted from those in a database, Kyoto Encyclopedia of Genes and Genomes (https://www.genome.jp/kegg/). Uncropped gel images of the gel images shown in Supplementary Figs. 2 and 3 are displayed in Supplementary Fig. 23. All data for bar graphs in Fig. 4a–c are shown in Supplementary Data 1. Artificial gene sequences encoding *Ht*-R15P isomerase, *Hs*-RbsK, *Ht*-RbsK, *Hx*-RbsK, *Ht*-Urdpase1, *Hx*-Urdpase1, *Hl*-Urdpase1, *Hx*-HAD hydrolase, *Hx*-FucA, and *Hs*-GaR are deposited in GenBank with the accession numbers LC735265, LC735266, LC735267, LC735268, LC735269, LC735270, LC735271, LC735272, LC735273, and LC735274, respectively.

## References

[1] Bräsen C, Esser D, Rauch B, Siebers B (2014). Carbohydrate metabolism in *Archaea*: current insights into unusual enzymes and pathways and their regulation. Microbiol. Mol. Biol. Rev..

[2] Sato T, Atomi H (2011). Novel metabolic pathways in *Archaea*. Curr. Opin. Microbiol..

[3] Verhees CH (2003). The unique features of glycolytic pathways in Archaea. Biochem. J..

[4] Wamelink MM, Struys EA, Jakobs C (2008). The biochemistry, metabolism and inherited defects of the pentose phosphate pathway: a review. J. Inherit. Metab. Dis..

[5] Kato N, Yurimoto H, Thauer RK (2006). The physiological role of the ribulose monophosphate pathway in bacteria and archaea. Biosci. Biotechnol. Biochem..

[6] Orita I (2006). The ribulose monophosphate pathway substitutes for the missing pentose phosphate pathway in the archaeon *Thermococcus kodakaraensis*. J. Bacteriol..

[7] Soderberg T (2005). Biosynthesis of ribose-5-phosphate and erythrose-4-phosphate in archaea: a phylogenetic analysis of archaeal genomes. Archaea.

[8] Aitken DM, Brown AD (1969). Citrate and glyoxylate cycles in the halophil, *Halobacterium salinarium*. Biochim. Biophys. Acta.

[9] Falb M (2008). Metabolism of halophilic archaea. Extremophiles.

[10] Pickl A, Schönheit P (2015). The oxidative pentose phosphate pathway in the haloarchaeon *Haloferax volcanii* involves a novel type of glucose-6-phosphate dehydrogenase-The archaeal Zwischenferment. FEBS Lett..

[11] Bult CJ (1996). Complete genome sequence of the methanogenic archaeon, *Methanococcus jannaschii*. Science.

[12] Grochowski LL, Xu H, White RH (2005). Ribose-5-phosphate biosynthesis in *Methanocaldococcus jannaschii* occurs in the absence of a pentose-phosphate pathway. J. Bacteriol..

[13] Aono R, Sato T, Imanaka T, Atomi H (2015). A pentose bisphosphate pathway for nucleoside degradation in Archaea. Nat. Chem. Biol..

[14] Aono R (2012). Enzymatic characterization of AMP phosphorylase and ribose-1,5-bisphosphate isomerase functioning in an archaeal AMP metabolic pathway. J. Bacteriol..

[15] Sato T, Atomi H, Imanaka T (2007). Archaeal type III RuBisCOs function in a pathway for AMP metabolism. Science.

[16] Ezaki S, Maeda N, Kishimoto T, Atomi H, Imanaka T (1999). Presence of a structurally novel type ribulose-bisphosphate carboxylase/oxygenase in the hyperthermophilic archaeon, *Pyrococcus kodakaraensis* KOD1. J. Biol. Chem..

[17] Kitano K (2001). Crystal structure of a novel-type archaeal rubisco with pentagonal symmetry. Structure.

[18] Maeda N (1999). Ribulose bisphosphate carboxylase/oxygenase from the hyperthermophilic archaeon *Pyrococcus kodakaraensis* KOD1 is composed solely of large subunits and forms a pentagonal structure. J. Mol. Biol..

[19] Watson GM, Yu JP, Tabita FR (1999). Unusual ribulose 1,5-bisphosphate carboxylase/oxygenase of anoxic *Archaea*. J. Bacteriol..

[20] Hove-Jensen B, Brodersen DE, Manav MC (2019). The prodigal compound: return of ribosyl 1,5-bisphosphate as an important player in metabolism. Microbiol. Mol. Biol. Rev..

[21] Wrighton KC (2016). RubisCO of a nucleoside pathway known from Archaea is found in diverse uncultivated phyla in bacteria. ISME J..

[22] Hansen T, Reichstein B, Schmid R, Schönheit P (2002). The first archaeal ATP-dependent glucokinase, from the hyperthermophilic crenarchaeon *Aeropyrum pernix*, represents a monomeric, extremely thermophilic ROK glucokinase with broad hexose specificity. J. Bacteriol..

[23] Kengen SW, Tuininga JE, de Bok FA, Stams AJ, de Vos WM (1995). Purification and characterization of a novel ADP-dependent glucokinase from the hyperthermophilic archaeon *Pyrococcus furiosus*. J. Biol. Chem..

[24] Sakuraba H, Mitani Y, Goda S, Kawarabayasi Y, Ohshima T (2003). Cloning, expression, and characterization of the first archaeal ATP-dependent glucokinase from aerobic hyperthermophilic archaeon *Aeropyrum pernix*. J. Biochem..

[25] Hansen T, Schönheit P (2000). Purification and properties of the first-identified, archaeal, ATP-dependent 6-phosphofructokinase, an extremely thermophilic non-allosteric enzyme, from the hyperthermophile *Desulfurococcus amylolyticus*. Arch. Microbiol..

[26] Tuininga JE (1999). Molecular and biochemical characterization of the ADP-dependent phosphofructokinase from the hyperthermophilic archaeon *Pyrococcus furiosus*. J. Biol. Chem..

[27] Makino Y (2016). An archaeal ADP-dependent serine kinase involved in cysteine biosynthesis and serine metabolism. Nat. Commun..

[28] Mori Y (2021). Identification and enzymatic analysis of an archaeal ATP-dependent serine kinase from the hyperthermophilic archaeon *Staphylothermus marinus*. J. Bacteriol..

[29] Aziz I (2018). A phosphofructokinase homolog from *Pyrobaculum calidifontis* displays kinase activity towards pyrimidine nucleosides and ribose 1-phosphate. J. Bacteriol..

[30] Ng WV (2000). Genome sequence of *Halobacterium* species NRC-1. Proc. Natl Acad. Sci. USA.

[31] Anderson I (2012). Complete genome sequence of *Halopiger xanaduensis* type strain (SH-6^T^). Stand. Genom. Sci..

[32] Anderson IJ (2016). Complete genome sequence of the Antarctic *Halorubrum lacusprofundi* type strain ACAM 34. Stand. Genom. Sci..

[33] Saunders E (2010). Complete genome sequence of *Haloterrigena turkmenica* type strain (4k^T^). Stand. Genom. Sci..

[34] Caparrós-Martín JA, McCarthy-Suárez I, Culiáñez-Macià FA (2013). HAD hydrolase function unveiled by substrate screening: enzymatic characterization of *Arabidopsis thaliana* subclass I phosphosugar phosphatase AtSgpp. Planta.

[35] Imker HJ, Fedorov AA, Fedorov EV, Almo SC, Gerlt JA (2007). Mechanistic diversity in the RuBisCO superfamily: the “enolase” in the methionine salvage pathway in *Geobacillus kaustophilus*. Biochemistry.

[36] Ghalambor MA, Heath EC (1962). The metabolism of l-fucose. II. The enzymatic cleavage of l-fuculose 1-phosphate. J. Biol. Chem..

[37] Koga Y, Kyuragi T, Nishihara M, Sone N (1998). Did archaeal and bacterial cells arise independently from noncellular precursors? A hypothesis stating that the advent of membrane phospholipid with enantiomeric glycerophosphate backbones caused the separation of the two lines of descent. J. Mol. Evol..

[38] Nishihara M, Koga Y (1995). *sn*-Glycerol-1-phosphate dehydrogenase in *Methanobacterium thermoautotrophicum*: key enzyme in biosynthesis of the enantiomeric glycerophosphate backbone of ether phospholipids of archaebacteria. J. Biochem..

[39] Nishihara M, Koga Y (1997). Purification and properties of *sn*-glycerol-1-phosphate dehydrogenase from *Methanobacterium thermoautotrophicum*: characterization of the biosynthetic enzyme for the enantiomeric glycerophosphate backbone of ether polar lipids of Archaea. J. Biochem..

[40] Hove-Jensen B, Rosenkrantz TJ, Haldimann A, Wanner BL (2003). *Escherichia coli phnN*, encoding ribose 1,5-bisphosphokinase activity (phosphoribosyl diphosphate forming): dual role in phosphonate degradation and NAD biosynthesis pathways. J. Bacteriol..

