# Supplementary material for: A non-carboxylating pentose bisphosphate pathway in halophilic archaea
Source: Commun Biol. 2022 Nov 24;5:1290. doi: 10.1038/s42003-022-04247-2 (PMC9700705; doi:10.1038/s42003-022-04247-2)
Supplement: Supplementary file 1 — Supplementary_Information [file 42003_2022_4247_MOESM1_ESM.pdf]

Supplementary Information

A non-carboxylating pentose bisphosphate pathway in halophilic archaea

Takaaki Sato[1,2], Sanae (Hodo) Utashima[1], Yuta Yoshii[1], Kosuke Hirata[1], Shuichiro Kanda[1], Yushi Onoda[1], Jian-qiang Jin[1], Suyi Xiao[1], Ryoko Minami[1], Hikaru Fukushima[1], Ayako Noguchi[3], Yoshiyuki Manabe[3,4], Koichi Fukase[3,4], Haruyuki Atomi[1,2]*

Affiliations:

[1] Department of Synthetic Chemistry and Biological Chemistry, Graduate School of Engineering, Kyoto University, Kyoto, Japan.

[2] Integrated Research Center for Carbon Negative Science, Kyoto University, Kyoto, Japan.

[3] Department of Chemistry, Graduate School of Science, Osaka University, Osaka, Japan.

[4] Forefront Research Center, Osaka University, Osaka, Japan

*Correspondence to: atomi@sbchem.kyoto-u.ac.jp

Supplementary Table 1. Tested phosphate acceptors and reaction conditions for kinase activity measurement of the *Hx*-RbsK protein.

		Buffer 50 mM each	NaCl conc.	NTPs conc.	Reaction temp.	Reaction time	Protein amount (/100 µl)
Uridine	Ns	Tris-HCl + Bicine-NaOH (pH 8.3)	1.8 M	4 mM ATP<br>1.5 mM GTP<br>1.5 mM UTP<br>1.5 mM CTP<br>1.5 mM TTP	42 ^{o}C	30 min	2.5 µg
Adenosine	Ns						
2'-Deoxyadenosine	Ns						
2'-Deoxyguanosine	Ns						
2'-Deoxycytidine	Ns						
2'-Deoxyuridine	Ns						
D-Erythrose	Ald						
L-Glucose	Ald						
L-Mannose	Ald						
L-Arabinose	Ald						
D-Arabinose	Ald						
D-Allose	Ald						
D-Altrose	Ald						
D-Talose	Ald						
D-Mannose	Ald						
D-Xylulose	K						
D-Ribulose	K						
D-Fructose	K						
L-Fructose	K						
D-Tagatose	K						
D-Sorbose	K						
AMP	Nt						
GMP	Nt						
UMP	Nt						
TMP	Nt						
D-Fructose 1-phosphate	Sp						
D-Glucose 1-phosphate	Sp						
D-Glucose 6-phosphate	Sp						
D-Ribose 5-phosphate	Sp						
D-Ribulose 5-phosphate	Sp						
Maltose	D						
Lactose	D						
Sucrose	D						
D-(+)-Trehalose	D						
myo-Inositol	Sa						
D-(+)-Cellobiose	Sa						
Glycerol	Sa						
D-Mannitol	Sa						
Adonitol	Sa						
DL-Threitol	Sa						
Xylitol	Sa						
meso-Erythritol	Sa						
Maltitol	Sa						
D-Glucuronic acid	Ds						
D-(+)-Galacturonic acid	Ds						
2-Deoxy-D-ribose	Ds						
2-Deoxy-D-glucose	Ds						
L-Fucose	Ds						

		Buffer 50 mM each	NaCl conc.	NTPs conc.	Reaction temp.	Reaction time	Protein amount (/100 µl)
Guanosine	Ns	Tris-HCl + Bicine-NaOH (pH 8.4)	4 M	2 mM ATP<br>2 mM GTP<br>2 mM UTP<br>2 mM CTP<br>2 mM TTP	42 ^{o}C	60 min	2.0 µg
Cytidine	Ns						
Thymidine	Ns						
Inosine	Ns						
Xanthosine	Ns						
D-Ribose	Ald						
D-Xylose	Ald						
D-Glucose	Ald						
D-Galactose	Ald	Tris-HCl + Bicine-NaOH (pH 8.3)	1.8 M	4 mM ATP<br>2 mM GTP<br>2 mM UTP<br>2 mM CTP	42 ^{o}C	30 min	2.5 µg
D-Lyxose	Ald						
D-(+)-Glucosamine	As						
D-(+)-Galactosamine	As						
N-Acetylneuraminic acid	As						
N-Acetyl-D-galactosamine	As						
N-Acetylmuramic acid	As						
N-Acetyl-D-mannosamine	As						
Ethanol	Alc						
Isopropanol	Alc						
D-Fructose 6-phosphate	Sp						
Lactulose	D						
Isomaltose	D						
D-Arabitol	Sa						
L-Arabitol	Sa						
Dulcitol	Sa						
L-Iditol	Sa						
D-Lactitol	Sa						
Volemitol	Sa						
L-Rhamnose	Ds						
D-Gluconic acid	Aac						
D-Psicose	K	Tris-HCl + Bicine-NaOH (pH 8.3)	1.8 M	4 mM ATP	42 ^{o}C	15 min	2.5 µg
D-Ribose 1-phosphate	Sp						
α-L-Fucose 1-phosphate	Sp						
β-L-Fucose 1-phosphate	Sp						
D-Fucose	Ds						
D-Arabinose 5-phosphate	Sp	Tris-HCl + Bicine-NaOH (pH 8.3)	1.8 M	4 mM ATP	47 ^{o}C	15 min	4.0 µg
D-Galactose 1-phosphate	Sp						
D-Mannose 6-phosphate	Sp						

Ns: Nculeoside
Ald: Aldose
K: Ketose
Nt: Nucleotide
Sp: Sugar phosphate
D: Disaccharide
Sa: Sugar alcohol
Ds: Deoxy sugar
As: Amino sugar
Alc: Alcohol
Aac: Aldonic acid

Supplementary Table 2. Strains and plasmids used in this study.

Strain or Plasmid	Relevant characteristic	Source or Reference
Strains		
Escherichia coli		
DH5α	F^- Φ80d *lacZ* ΔM15 Δ(*lacZYA-argF*)U169 *deoR* *recA* 1 *endA* 1 *hsdR* 17(r_K^-, m_K^+) *phoA* *supE* 44 λ^- *thi* -1 *gyrA* 96 *relA* 1	Takara Bio (Shiga, Japan)
BL21 CodonPlus(DE3)-RIL	F^- *ompT* *hsdS* (r_B^- m_B^-) *dcm* + Tet^r *gal* λ (DE3) *endA* Hte [*argU* *ileY* *leuW* Cam^r]	Agilent Technology (Santa Clara, CA)
Rosetta (DE3)	F^- *ompT* *hsdS* $_B$(r_B^- m_B^-) *gal* *dcm* (DE3) pRARE2 (Cam^r)	Merck
Halobacterium salinarum		
NRC-1	Wild-type	RIKEN BioResource Research Center
Plasmids		
pET-21a(+)	Amp^r general expression vector	Merck
pET-Hs-R15P isomerase	pET-21a(+) derivative; VNG_1853G	This study
pET-Ht-R15P isomerase	pET-21a(+) derivative; Htur_0571*	This study
pET-Hs-RbsK	pET-21a(+) derivative; VNG_1851G*	This study
pET-Ht-RbsK	pET-21a(+) derivative; Htur_0569*	This study
pET-Hx-RbsK	pET-21a(+) derivative; Halxa_1682*	This study
pET-Hs-Urdpase1	pET-21a(+) derivative; VNG_1850G	This study
pET-Ht-Urdpase1	pET-21a(+) derivative; Htur_0567*	This study
pET-Hx-Urdpase1	pET-21a(+) derivative; Halxa_1684*	This study
pET-Hl-Urdpase1	pET-21a(+) derivative; Hlac_2318*	This study
pET-Hx-HAD hyrolase	pET-21a(+) derivative; Halxa_2271*	This study
pET-Hx-FucA	pET-21a(+) derivative; Halxa_2272*	This study
pET-Hs-GaR	pET-21a(+) derivative; VNG_6270G*	This study

* Modified sequences are shown in Supplementary Fig. 22.

Supplementary Table 3. Primers used in this study.

Number	Primer name	Oligo nucleotide sequence (5'-sequence-3')*	Intended use
1	expHs-R15Pi-F	GGGCATATGGTCACCGAGGACGTGCGG	For amplification of a R15P isomerase coding region to construct its expression vector
2	expHs-R15Pi-R	GGGGGATCCTCAGTCCTCGGCCCACGGTTCGGTGGT	
3	Hs-Urdpase1-F	GGGCATATGGCCAAACAACCCCACCTGCTCGTCGA	For amplification of a Urdpase1 coding region to construct its expression vector
4	Hs-Urdpase1-R	GGGGGATCCCTACGACAGGGCGACGACCGCGTCGAGGG	

*Underlined letters indicate restriction sites

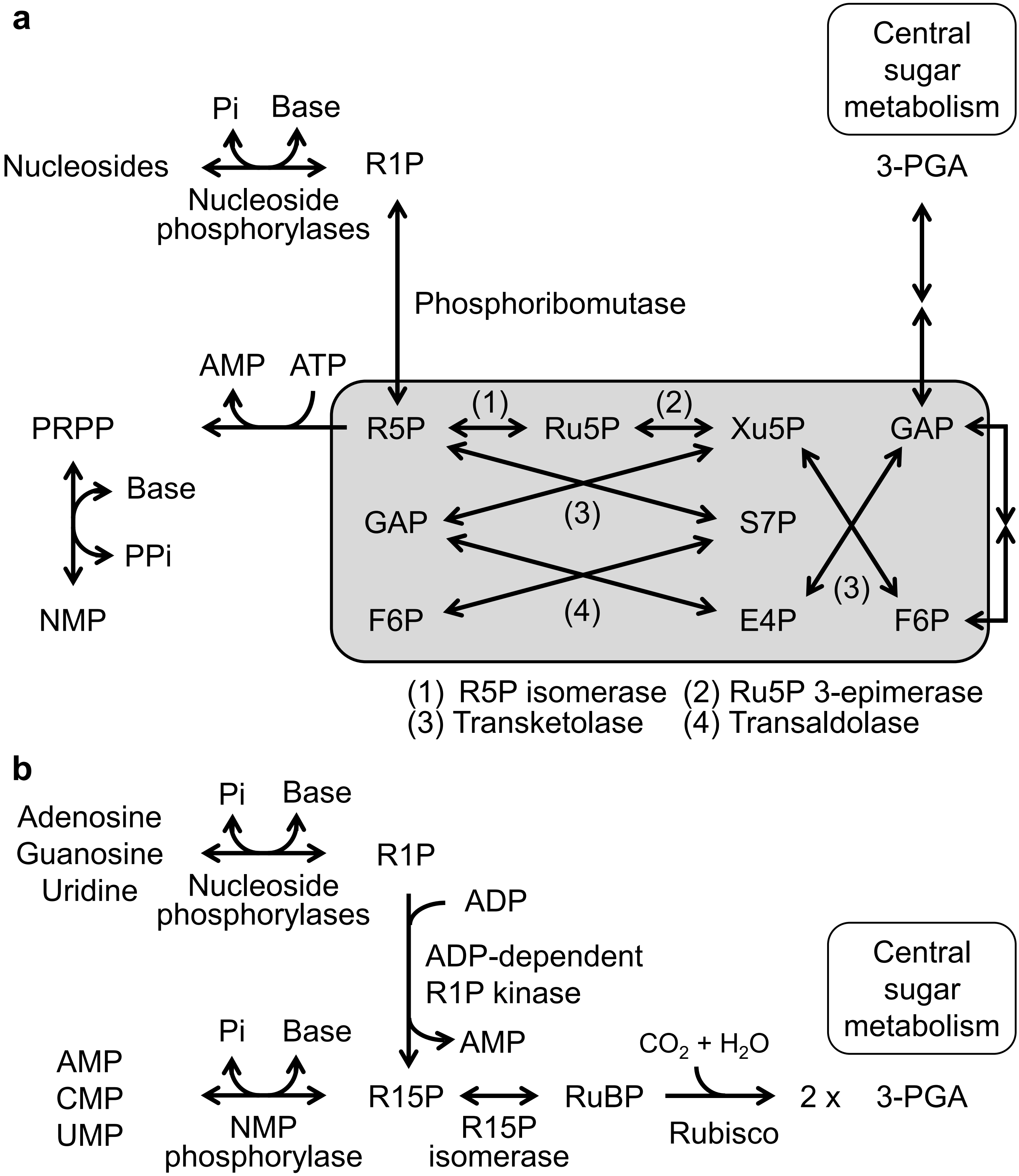

Supplementary Fig. 1. Nucleoside degradation pathways in Eucarya/Bacteria and *Thermococcus*.

a, Nucleoside degradation pathway in Eucarya and Bacteria. The metabolic route shaded in gray indicates the non-oxidative pentose phosphate pathway. **b**, Pentose bisphosphate pathway to degrade nucleosides and NMPs in *Thermococcus*. Abbreviations: R1P, ribose 1-phosphate; PRPP, phosphoribosyl pyrophosphate; NMP, nucleoside 5'-monophosphate; R5P, ribose 5-phosphate; Ru5P, ribulose 5-phosphate; Xu5P, xylulose 5-phosphate; GAP, glyceraldehyde 3-phosphate; S7P, sedoheptulose 7-phosphate; F6P, fructose 6-phosphate; E4P, erythrose 4-phosphate; R15P, ribose 1,5-bisphosphate, RuBP, ribulose 1,5-bisphosphate; 3-PGA, 3-phosphoglycerate.

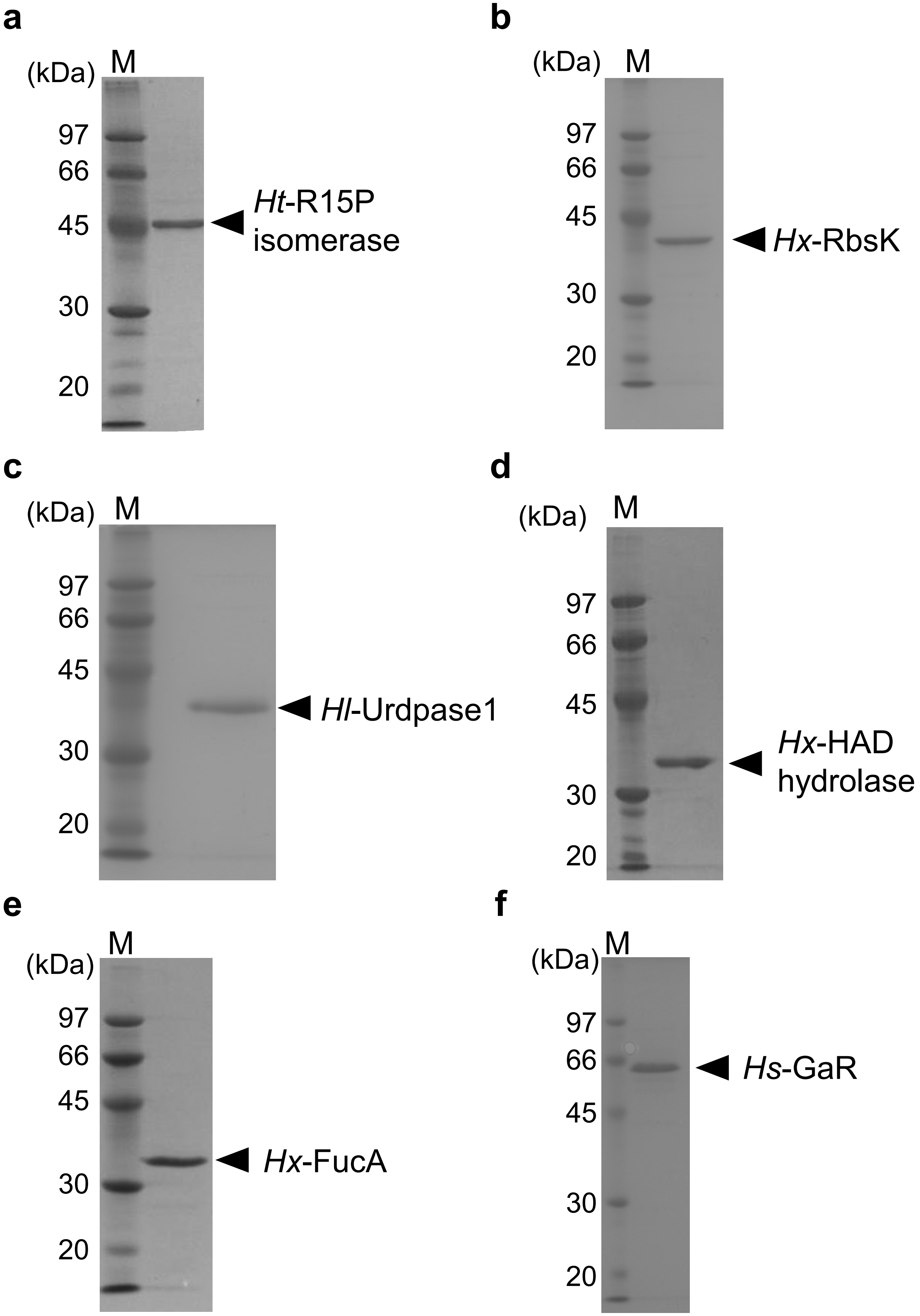

Supplementary Fig. 2. SDS-PAGE analysis of the purified recombinant proteins and native *Hs*-GaR protein.

a, *Ht*-R15P isomerase (1 μg), **b**, *Hx*-RbsK (2 μg), **c**, *Hl*-Urdpase1 (1 μg), **d**, *Hx*-HAD hydrolase (1 μg), **e**, *Hx*-FucA (2 μg), **f**, *Hs*-GaR (1 μg) after purification were separated by SDS-PAGE and stained with Coomassie brilliant blue. *Hs*-GaR is the native protein purified from *H. salinarum* cells, and all other proteins are recombinant proteins produced in *E. coli*. M indicates molecular mass marker.

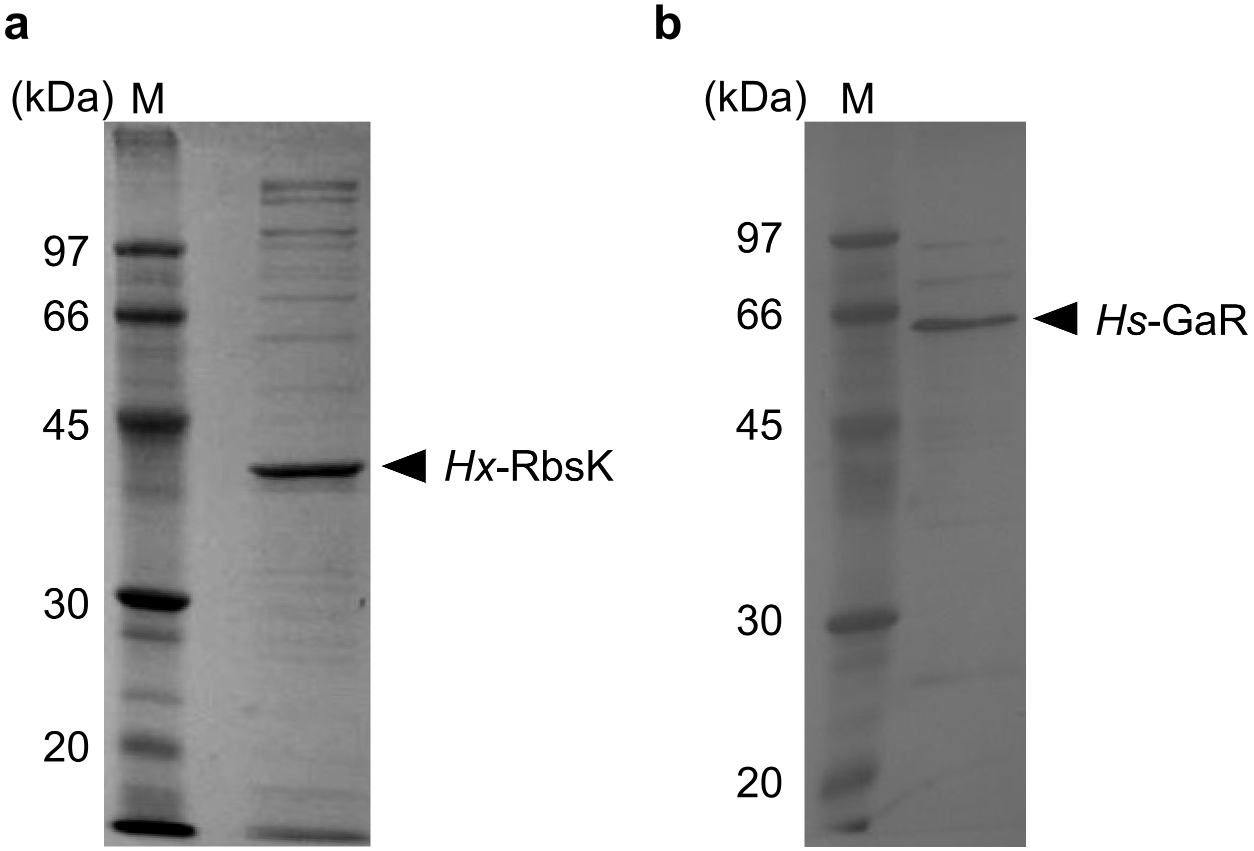

Supplementary Fig. 3. SDS-PAGE analysis of partially purified recombinant *Hx*-RbsK and *Hs*-GaR proteins.

a, *Hx*-RbsK (2 μg) and **b**, *Hs*-GaR (2 μg) after partial purification were separated by SDS-PAGE and stained with Coomassie brilliant blue. Both proteins are recombinant proteins produced in *E. coli*. M indicates molecular mass marker.

a

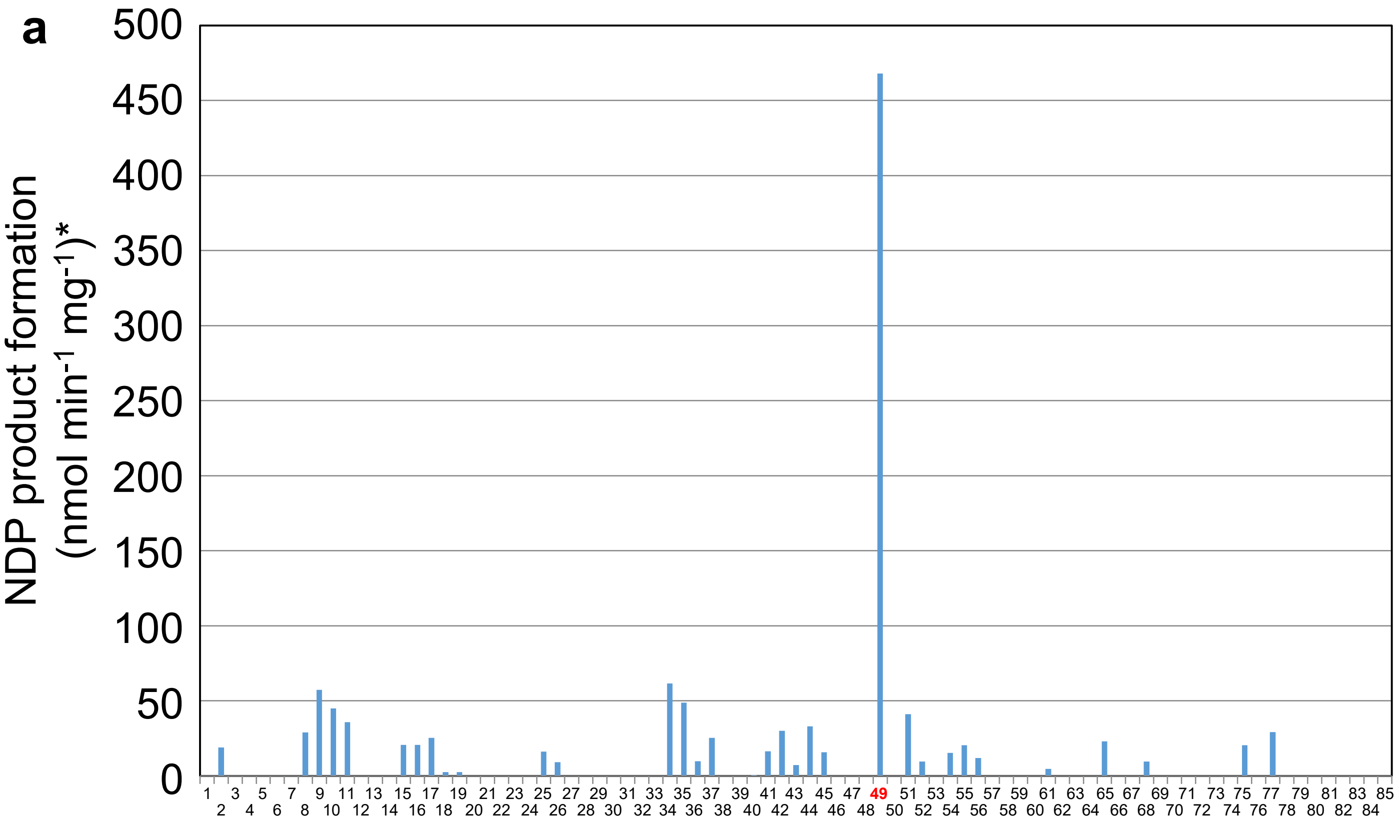

Nucleoside
- **1** Uridine
- **2** Adenosine
- **3** Guanosine ○
- **4** Cytidine ○
- **5** Thymidine ●
- **6** Inosine ●
- **7** Xanthosine □
- **8** 2'-Deoxyadenosine
- **9** 2'-Deoxyguanosine
- **10** 2'-Deoxycytidine
- **11** 2'-Deoxyuridine

Aldose
- **12** D-Ribose □
- **13** D-Xylose ■
- **14** D-Glucose ■
- **15** D-Erythrose ☆
- **16** L-Glucose ☆
- **17** L-Mannose ★
- **18** L-Arabinose △
- **19** D-Arabinose △
- **20** D-Allose
- **21** D-Altrose
- **22** D-Talose
- **23** D-Mannose
- **24** D-Galactose
- **25** D-Lyxose

Amino sugar
- **26** D-Glucosamine
- **27** D-Galactosamine
- **28** *N*-Acetylneuraminic acid
- **29** *N*-Acetyl-D-galactosamine
- **30** *N*-Acetylmuramic acid
- **31** *N*-Acetyl-D-mannnosamine

Alcohol
- **32** Ethanol
- **33** Isopropanol

Ketose
- **34** D-Xylulose
- **35** D-Ribulose
- **36** D-Fructose
- **37** L-Fructose ★
- **38** D-Tagatose
- **39** D-Sorbose
- **40** D-Psicose

Nucleotide
- **41** AMP
- **42** GMP
- **43** UMP
- **44** TMP

Sugar phosphate
- **45** D-Fructose 1-phosphate
- **46** D-Fructose 6-phosphate
- **47** D-Glucose 1-phosphate
- **48** D-Glucose 6-phosphate
- **49** D-Ribose 1-phosphate
- **50** D-Ribose 5-phosphate
- **51** D-Ribulose 5-phosphate
- **52** α-L-Fucose 1-phosphate
- **53** β-L-Fucose 1-phosphate
- **54** D-Arabinose 5-phosphate
- **55** D-Galactose 1-phosphate
- **56** D-Mannose 6-phosphate

Disaccharide
- **57** Maltose
- **58** Lactose
- **59** Sucrose
- **60** D-Trehalose
- **61** Lactulose
- **62** Isomaltose

Sugar alcohol
- **63** *myo*-Inositol ▲
- **64** D-Cellobiose ▲
- **65** Glycerol
- **66** D-Mannitol ◇
- **67** D-Arabitol
- **68** L-Arabitol
- **69** Adonitol ◇
- **70** DL-Threitol ◆
- **71** Xylitol ◆
- **72** *meso*-Erythritol ▽
- **73** Maltitol ▽
- **74** Dulcitol
- **75** L-Iditol
- **76** D-Lactitol
- **77** Volemitol

Deoxy sugar
- **78** D-Glucuronic acid
- **79** D-Galacturonic acid
- **80** 2-Deoxy-D-ribose
- **81** 2-Deoxy-D-glucose
- **82** L-Rhamnose
- **83** L-Fucose
- **84** D-Fucose

Aldonic acid
- **85** D-Gluconic acid

Supplementary Fig. 4. Kinase activity of the *Hx*-RbsK protein towards various phosphate acceptors. The 85 substrates (25 mM each) tested are listed below the graph. Reaction conditions are summarized in Supplementary Table 1. *NDP production was measured by quantifying the NDP produced from NTP with coupling enzymes and normalized by dividing by reaction time (min) and the amount of protein (mg). In several cases, two substrates were applied in a single reaction mixture and these substrates are indicated with common symbols to their right. The values given for each substrate are the NDP production values obtained with the mixture of substrates.

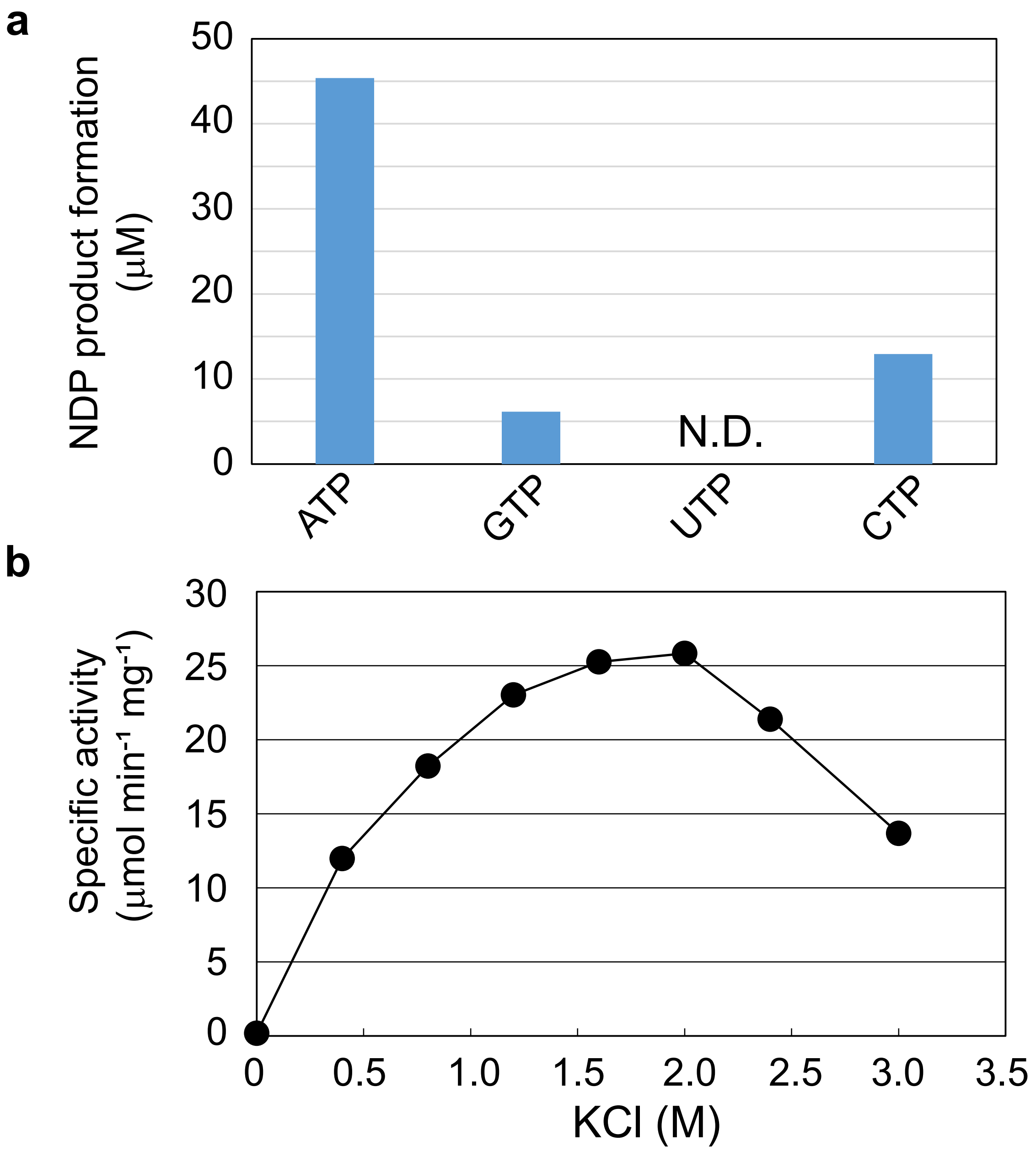

Supplementary Fig. 5. Characterization of the *Hx*-RbsK protein.

a, The ribose-1-phosphate (R1P) kinase activity of the *Hx*-RbsK protein was measured with different NTPs. Reactions were carried out at 42 °C for 5 min with 25 mM R1P, 20 mM $MgCl_2$, 0.8 M NaCl, 4 mM NTPs, and purified protein (1.2 μg per 100 μl). NDP production was measured by quantifying the NDP produced from NTP with coupling enzymes. **b,** R1P kinase activity of *Hx*-RbsK under different KCl concentrations. Reactions were carried out at 47 °C with 15 mM R1P, 20 mM $MgCl_2$, and 4 mM ATP. Product generation after reactions for 3, 4, and 5 min were quantified to calculate specific activities.

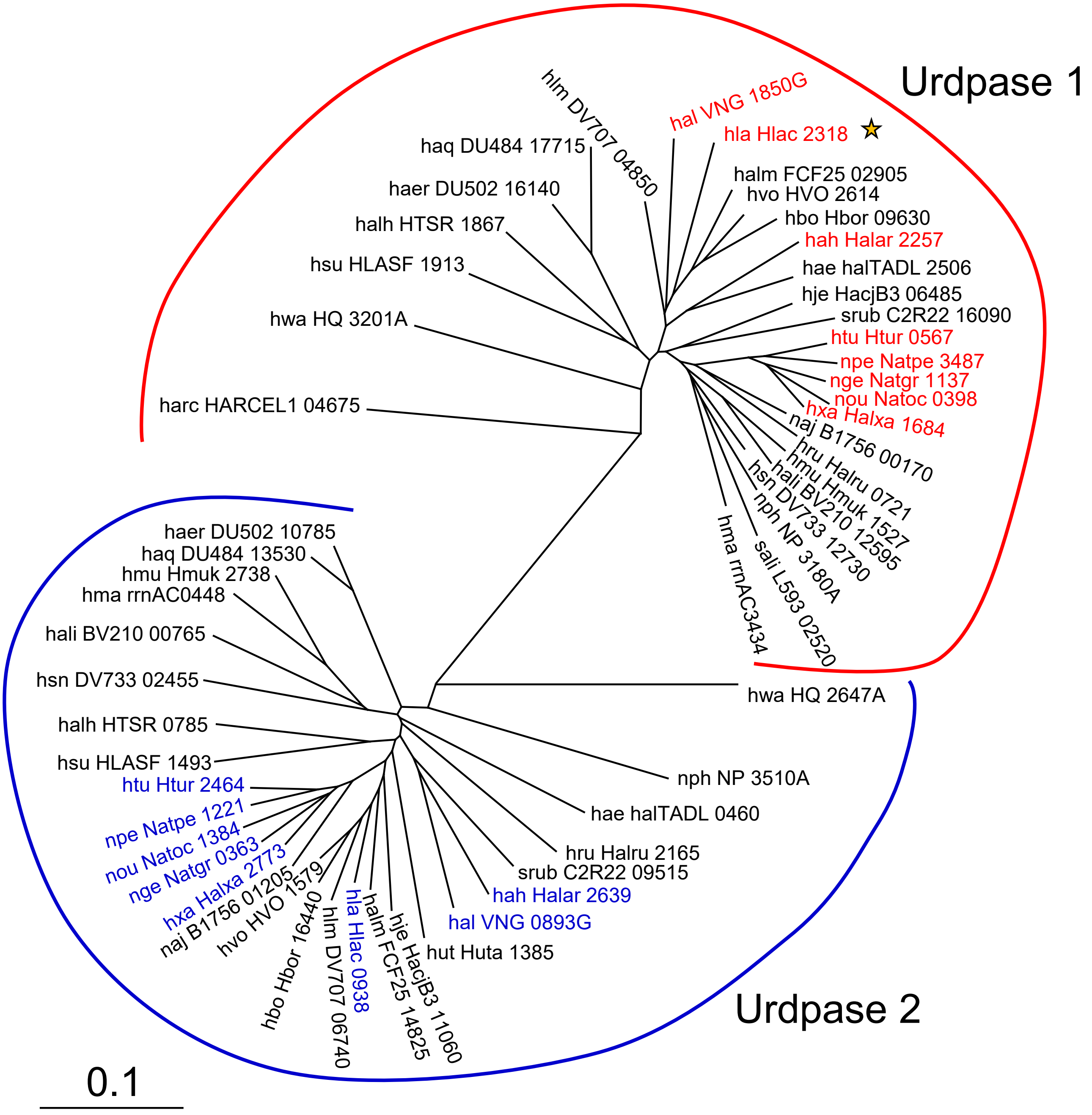

Supplementary Fig. 6. Phylogenetic analysis of Urdpase1 and Urdpase2 homologs in halophilic archaea.

The phylogenetic tree was constructed using the amino acid sequences of Urdpase 1 and Urdpase 2 from representative halophilic archaea including the 8 species shown in Fig. 3. Red and blue letters indicate Urdpase 1 and Urdpase 2 from the 8 species, respectively. The Urdpase 1 from *H. lacusprofundi*, characterized in this study, is indicated with a star. Each sequence is represented with a three- or four-letter organism code utilized in the KEGG BLAST Search and followed by a locus tag. The definitions of the organism codes are shown in Table 1.

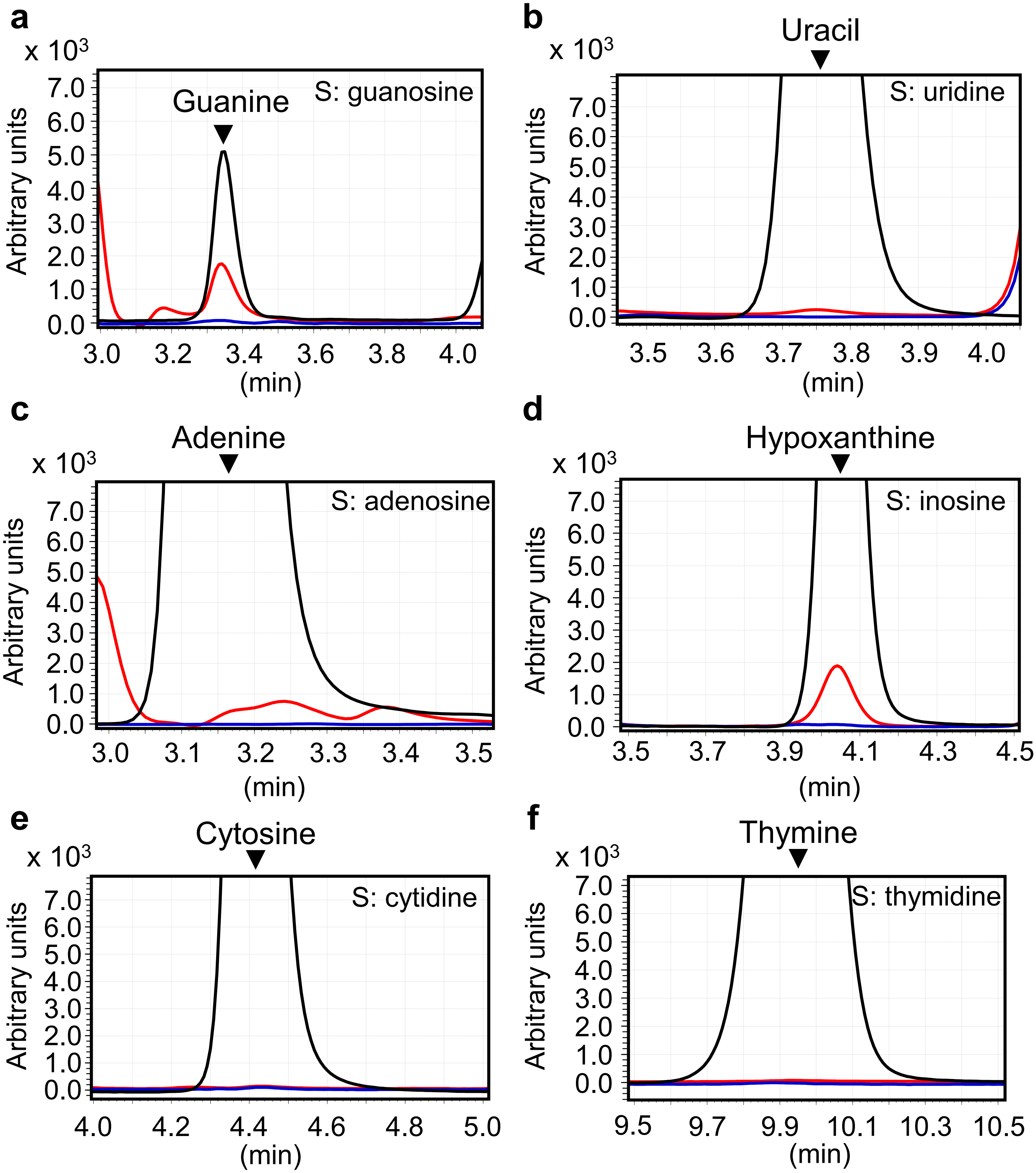

Supplementary Fig. 7. Nucleoside phosphorylase activity of the *Hl*-Urdpase1 protein toward various nucleosides. Reactions were performed at 37 ºC for 5 min in the presence of 1 mM nucleoside, 50 mM Pi, 2 M KCl, and purified protein (1 μg per 100 μl). The examined nucleosides are shown in the upper right corner of each chromatogram. Black, red, and blue lines indicate nucleobase standards, reaction products with and without enzyme, respectively.

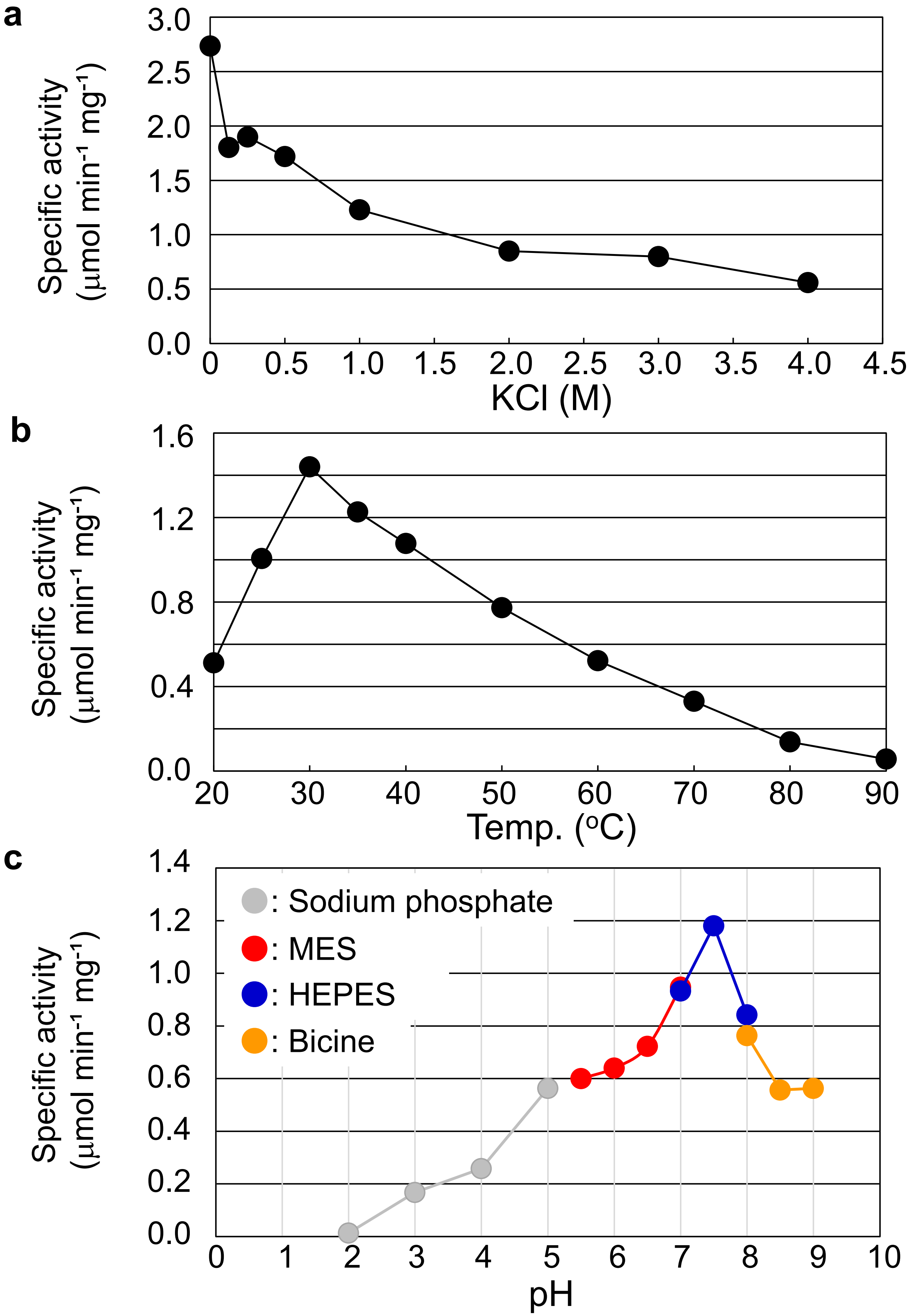

Supplementary Fig. 8. Enzymatic characterization of the *Hl*-Urdpase1 protein.
Effects of KCl concentration **(a)**, reaction temperature **(b)**, and pH **(c)** on the nucleoside phosphorylase activity of the *Hl*-Urdpase1 protein. All measurements were carried out in the presence of 2 mM guanosine and 50 mM sodium phosphate (pH 7.5). **a,** Reactions were carried out at 37 ^{o}C. **b**, Reactions were carried out with 2 M KCl. **c**, Reactions were carried out at 30 ^{o}C in the presence of 2 M KCl.

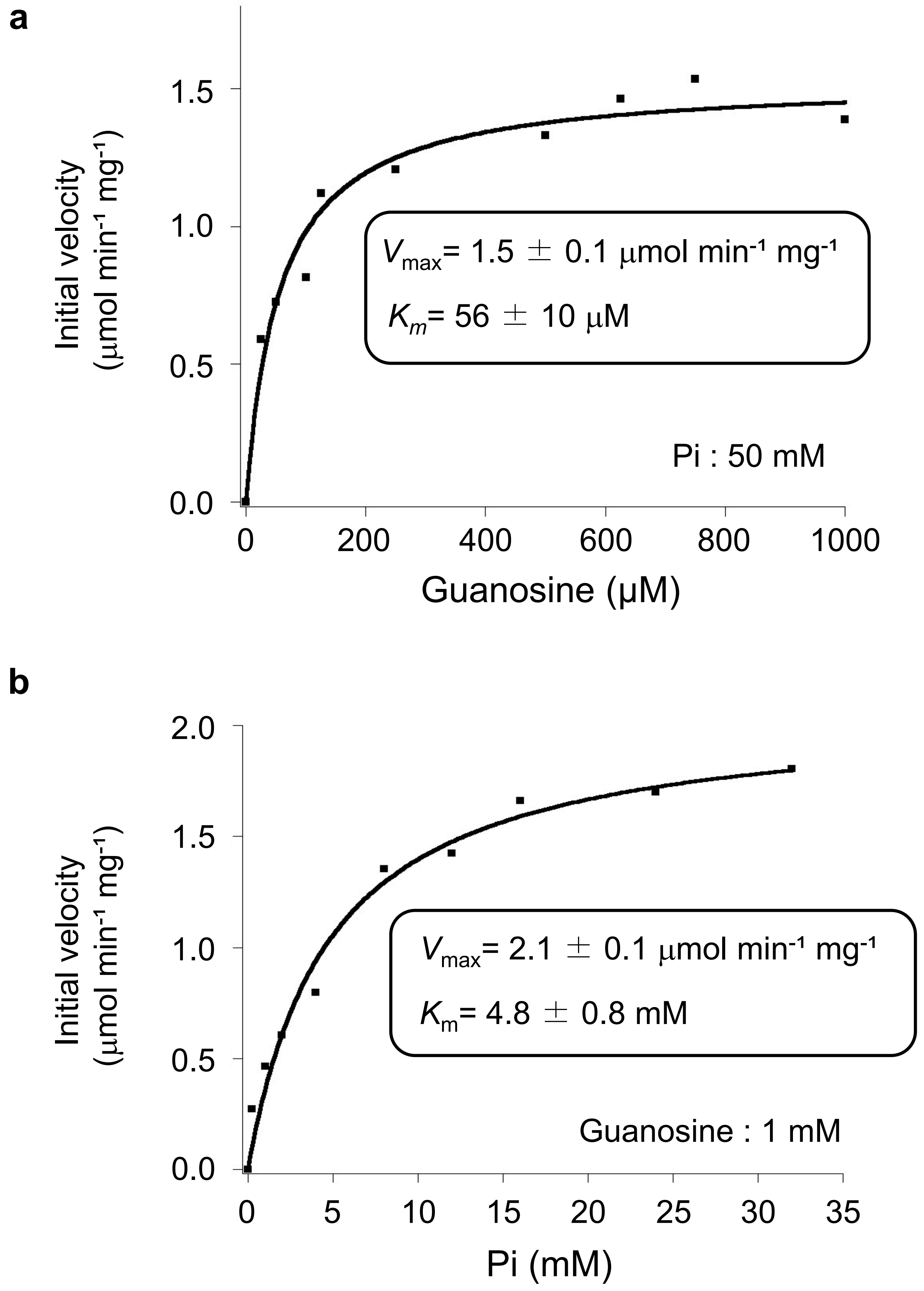

Supplementary Fig. 9. Kinetic analyses of the guanosine phosphorylase reaction catalyzed by the *Hl*-Urdpase1 protein.

a, Measurements were carried out with various concentrations of guanosine with 50 mM sodium phosphate (pH 7.5). **b,** Measurements were carried out with various concentrations of phosphate with 1 mM guanosine. In both cases, reactions were carried out at 30 ^{o}C in the presence of 2 M KCl. These [S]-v plots were fitted with the Michaelis-Menten equation, $v=V_{max}$ [S]/(K_m + [S]) [equation 1], where v is the initial velocity, V_{max} is the maximum velocity, and [S] is the substrate concentration.

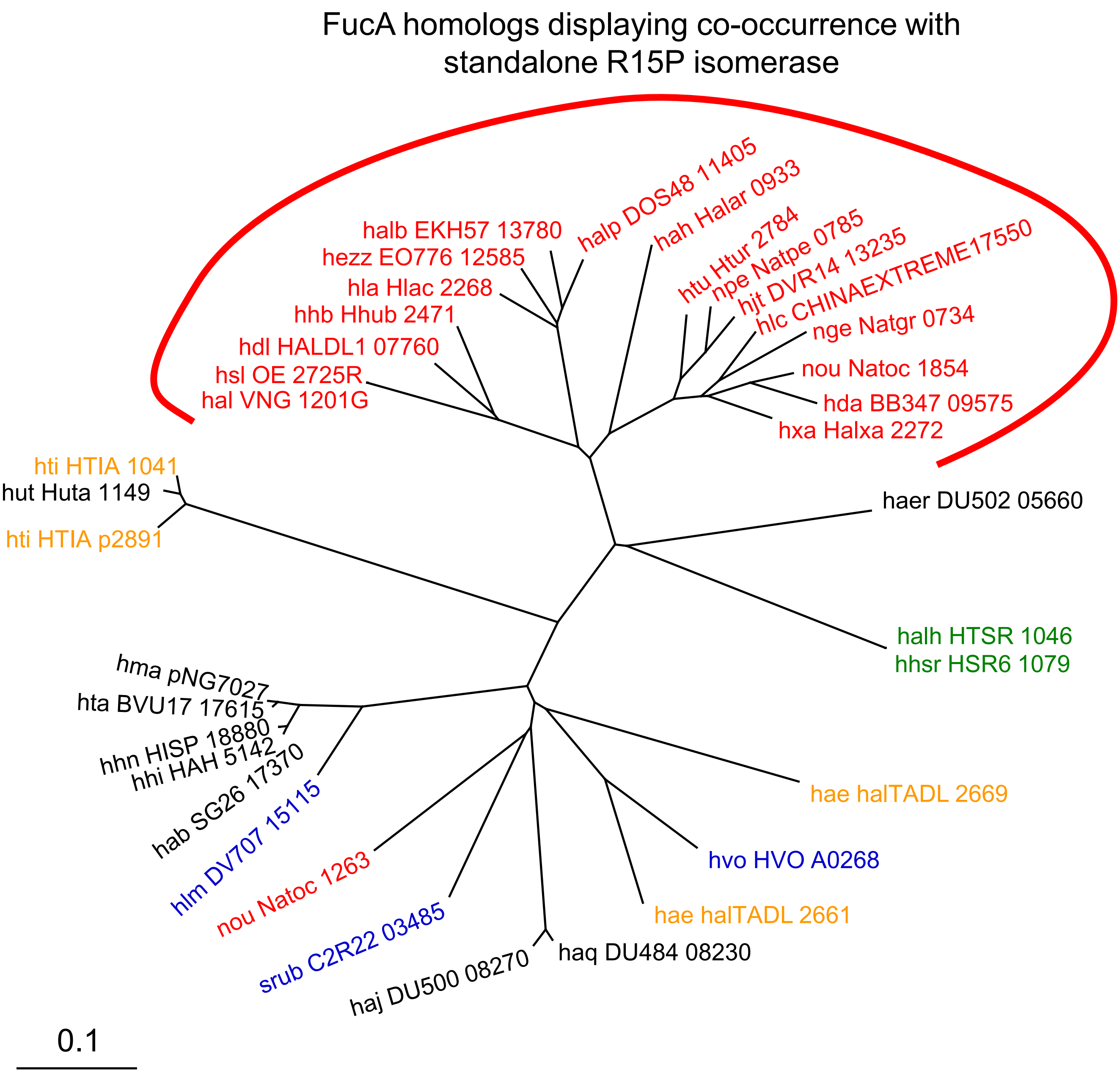

Supplementary Fig. 10. Phylogenetic analysis of FucA homologs from halophilic archaea. Proteins from halophilic archaea displaying similarity with *Hx*-FucA were examined. Protein sequences from halophilic archaea that harbor standalone R15P isomerase and HAD hydrolase are indicated in red, those from halophiles that harbor a complete NMP shunt are indicated in blue, those from halophiles that harbor R15P isomerase and Rubisco but not NMP phosphorylase are indicated in green, and those from halophiles that harbor a standalone R15P isomerase but do not harbor an HAD hydrolase are indicated in orange. Protein sequences in black are from halophilic archaea that do not possess R15P isomerase.

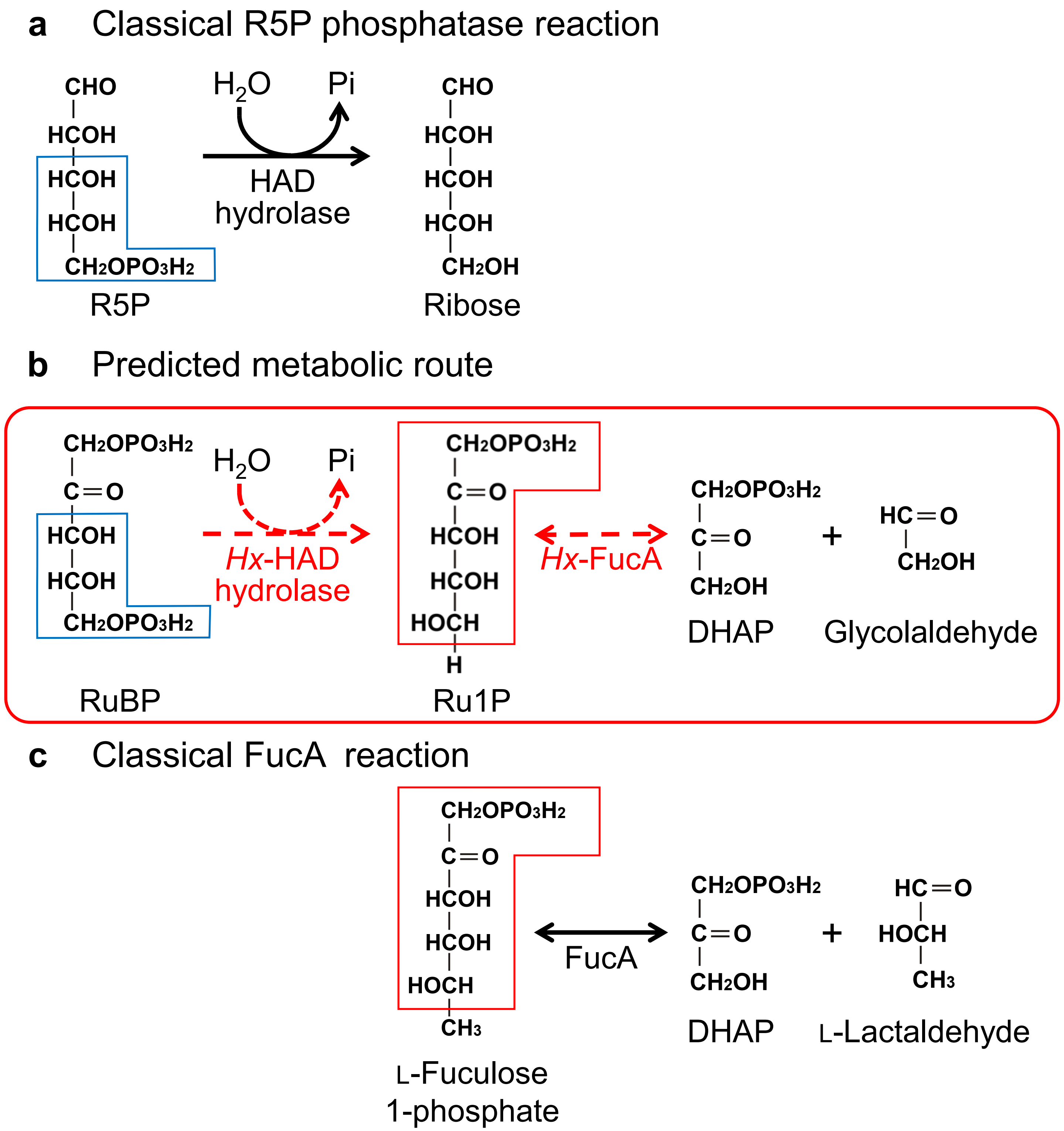

Supplementary Fig. 11. The predicted reactions catalyzed by proteins annotated as HAD hydrolase and FucA in halophilic archaea.
a, The classical R5P phosphatase reaction catalyzed by the HAD hydrolase from *Arabidopsis thaliana.* **b,** Predicted reactions catalyzed by proteins annotated as HAD hydrolase and FucA in halophilic archaea. **c,** The classical fuculose-1-phosphate aldolase reaction catalyzed by FucA. Identical chemical structures are boxed in red and blue. Abbreviations; R5P, ribose 5-phosphate; Ru1P, ribulose 1-phosphate; DHAP, dihydroxyacetone phosphate.

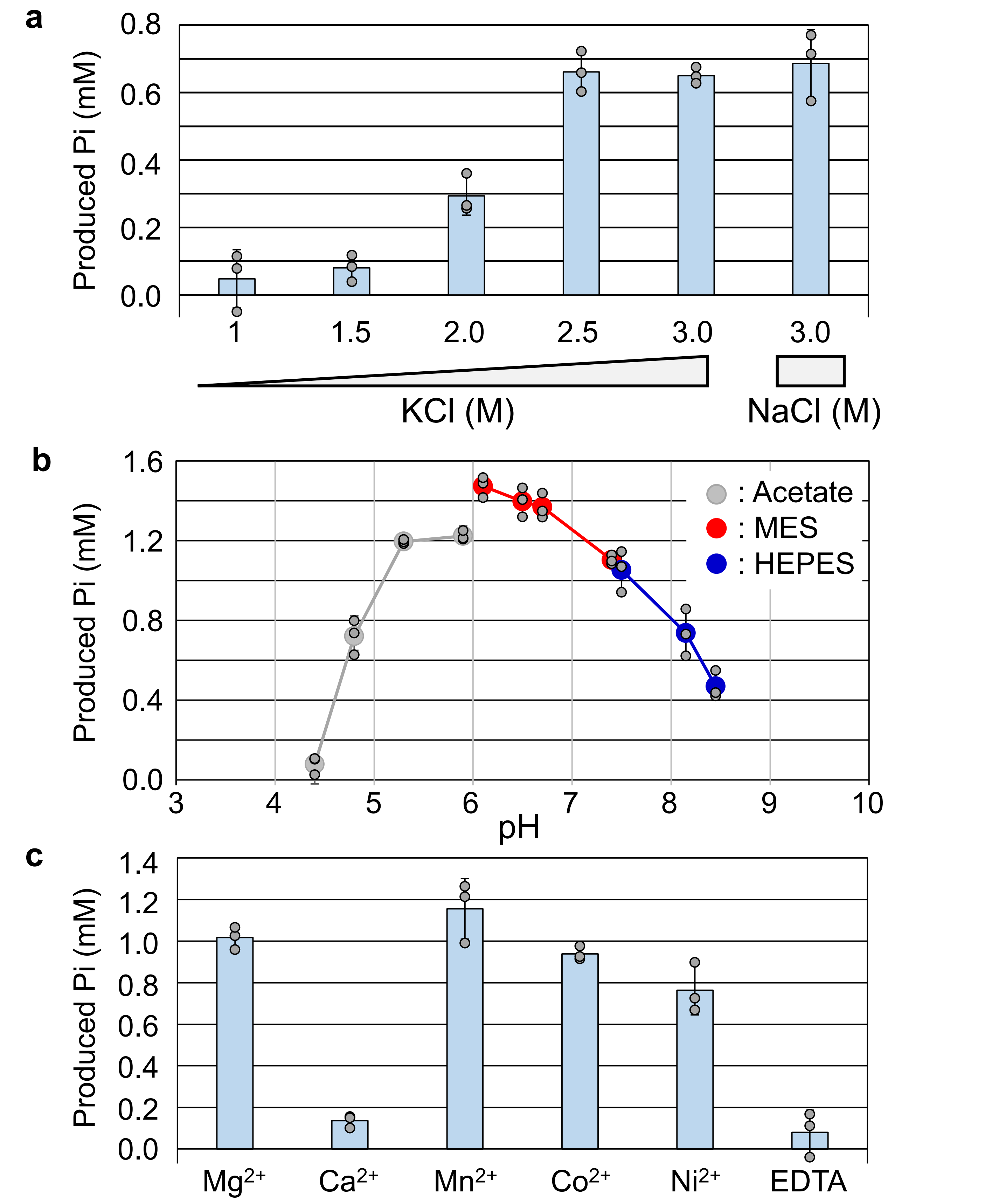

Supplementary Fig. 12. Enzymatic characterization of the *Hx*-HAD hydrolase protein. Effects of KCl concentration **(a)**, pH **(b)**, and metal ions **(c)** on the RuBP phosphatase activity of the *Hx*-HAD hydrolase protein. All measurements were performed at 37 °C for 10 min with purified protein (0.5 μg per 100 μl). The released phosphate was quantified with malachite green. **a**, Reaction mixture included 10 mM RuBP, 5 mM $MgCl_2$ in 50 mM Tris-HCl (pH 7.5) with various concentrations of KCl. **b**, Reaction mixture included 10 mM RuBP, 5 mM $MgCl_2$, 2.5 M KCl in 50 mM acetate (pH 4.4-5.9), 50 mM MES (pH 6.1-7.4), or 50 mM PIPES (pH 7.5-8.5). **c**, Reaction mixture included 10 mM RuBP, 2.5 M KCl, and 5 mM metal chlorides or EDTA in 50 mM MES (pH 6.1). The activities were calculated from three independent experiments. Error bars indicate standard deviations.

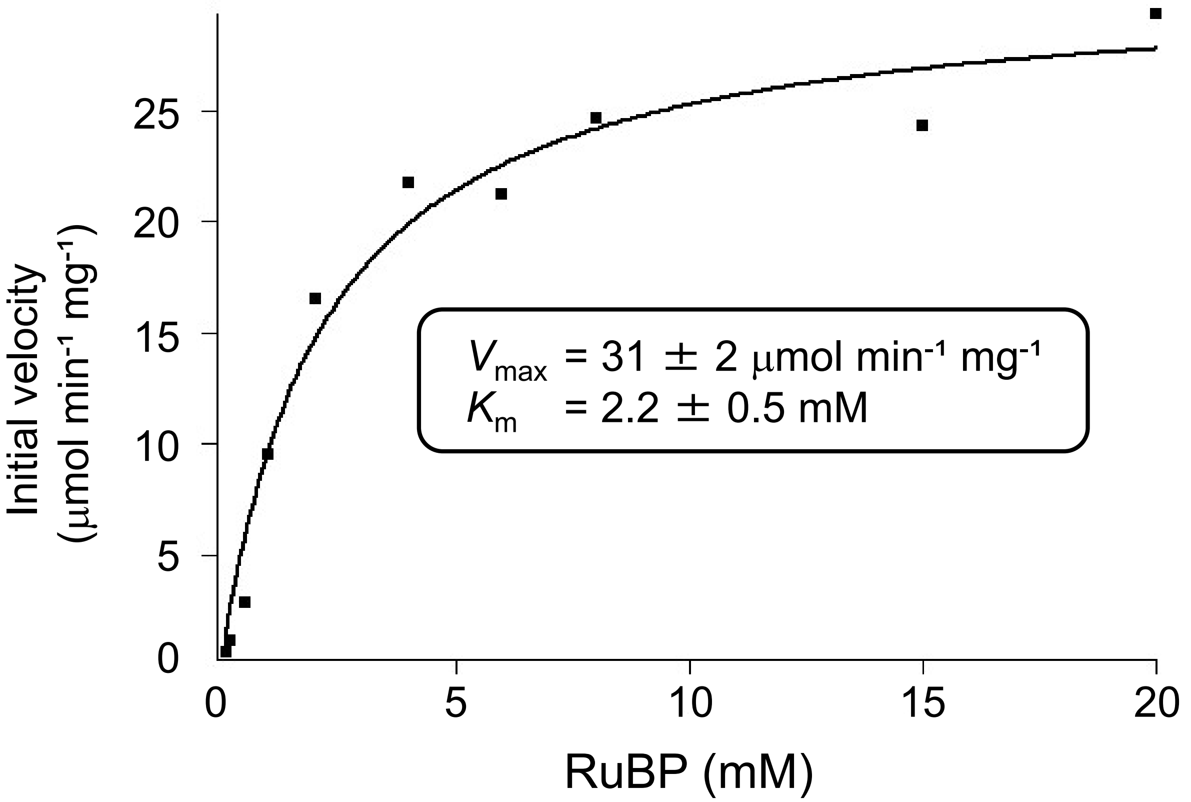

Supplementary Fig. 13. Kinetic analysis of the RuBP phosphatase reaction catalyzed by the *Hx*-HAD hydrolase protein.

Measurements were performed in the presence of 2 M KCl, 5 mM $MgCl_2$, and various concentrations of RuBP. The released phosphate was quantified with malachite green. The [S]-*v* plot was fitted with the Michaelis-Menten equation (equation 1).

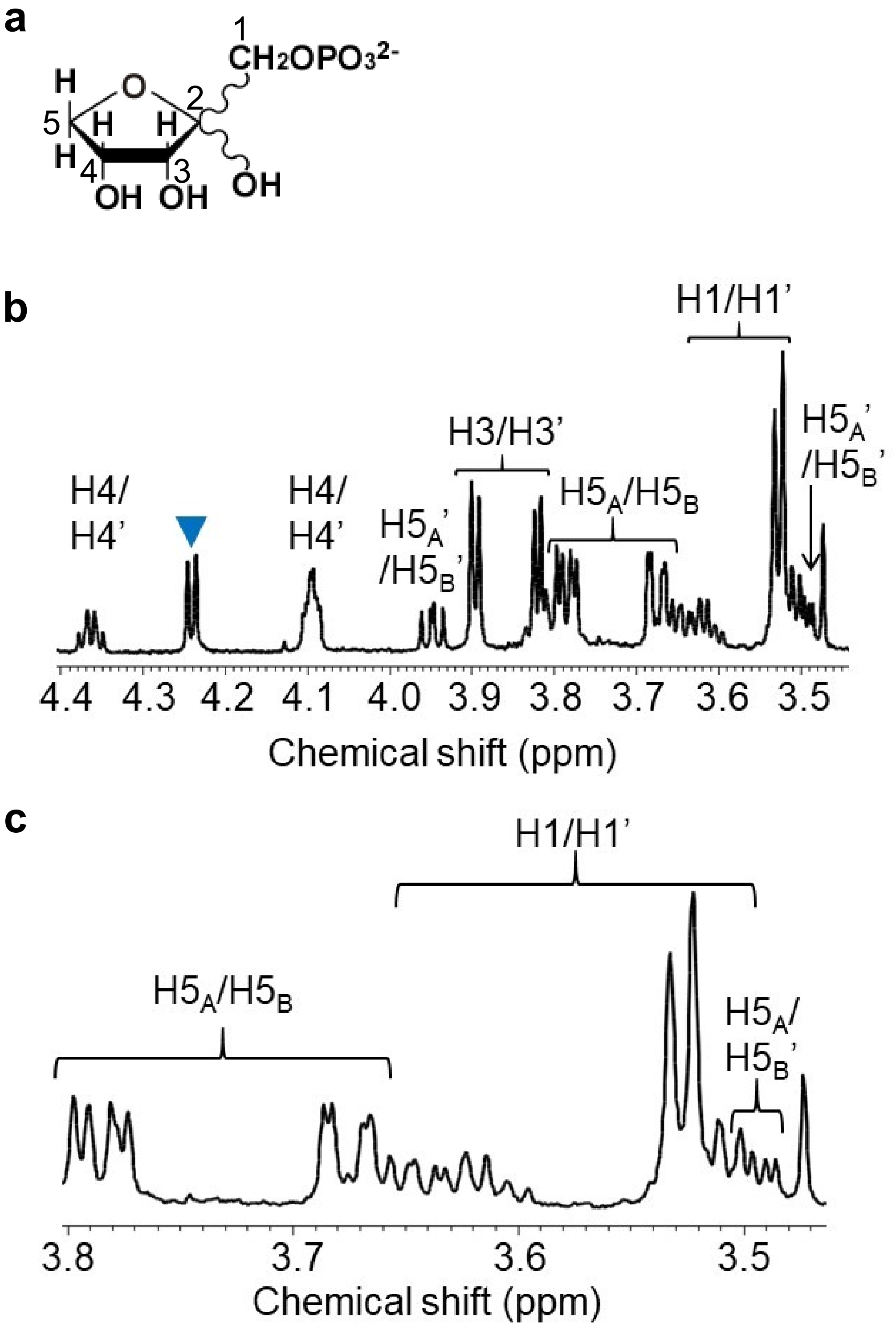

Supplementary Fig. 14. NMR analysis of the product of the RuBP phosphatase reaction. **a,** Chemical structure and carbon number of Ru1P. **b,** ^{1}H-NMR spectrum of reaction product of *Hx*-RuBP phosphatase in a solution containing D_2O was measured with 600 MHz at 5 ^{o}C. Blue arrowhead indicates chemical shift deriving from RuBP. **c,** The NMR spectrum around 3.65 ppm in figure **(b)** was enlarged. ^{1}H NMR spectrum (D_2O, 500 MHz) of Ru1P has been reported as follows[35]; δ 4.46 (q, J = 6.0, 6.3 Hz, H-4'), 4.19 (td, J = 2.8, 5.1 Hz, H-4), 4.06 - 3.61 (ABX, J = 6.1, 7.0, 9.0 Hz, H-5'), 4.03 (d, J = 5.2 Hz, H-3), 3.98 (d, J = 5.9 Hz, H-3'), 3.89 - 3.78 (ABX, J = 2.5, 5.0, 10.1 Hz, H-5), ~3.78 - 3.70 (ABX, H-1'), 3.76 - 3.66 (ABX, J = 5.4, 5.6, 11.0 Hz, H-1).

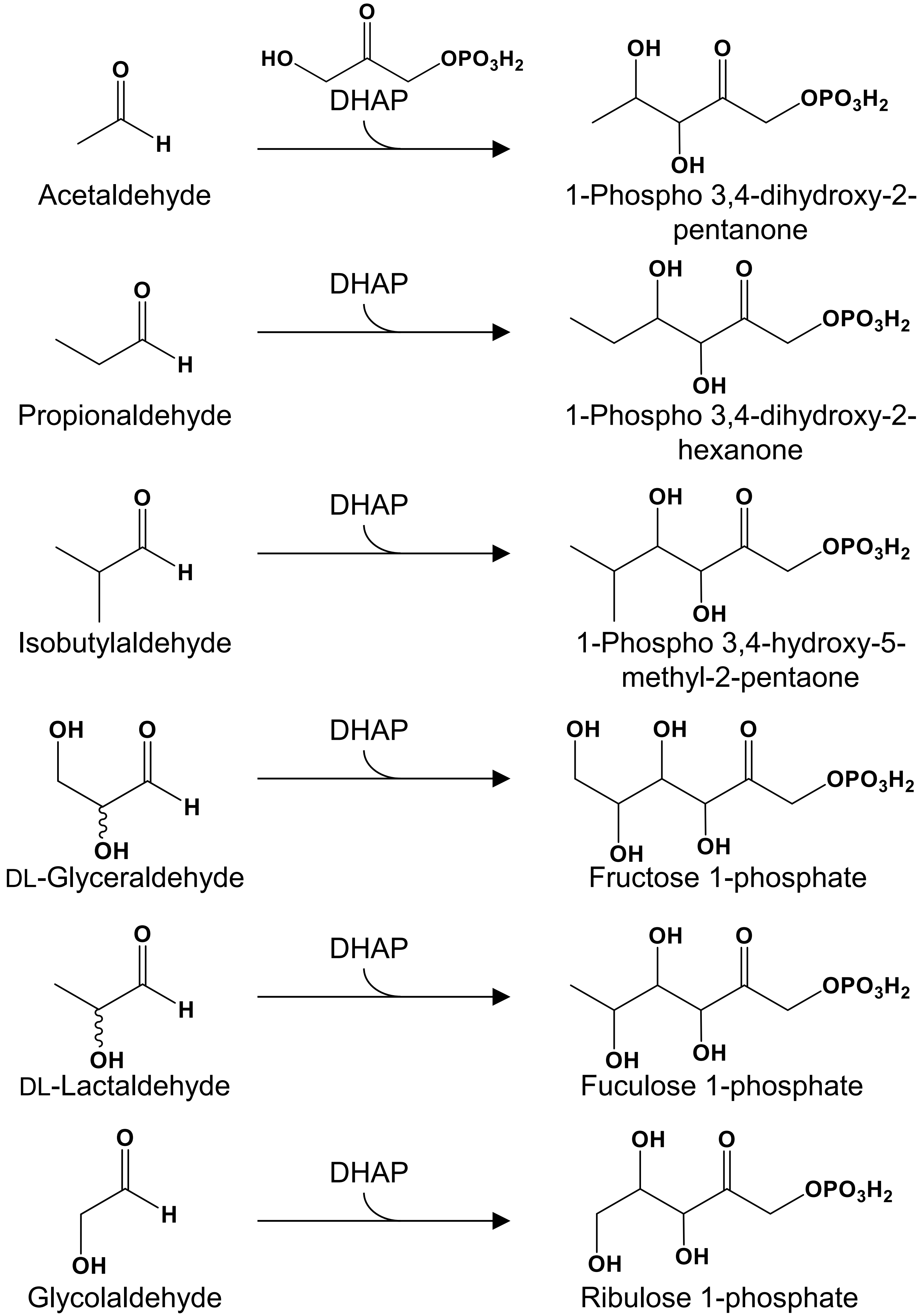

Supplementary Fig. 15. Chemical structures of the tested aldehydes and predicted reaction products of the aldolase reaction catalyzed by the *Hx*-FucA protein.

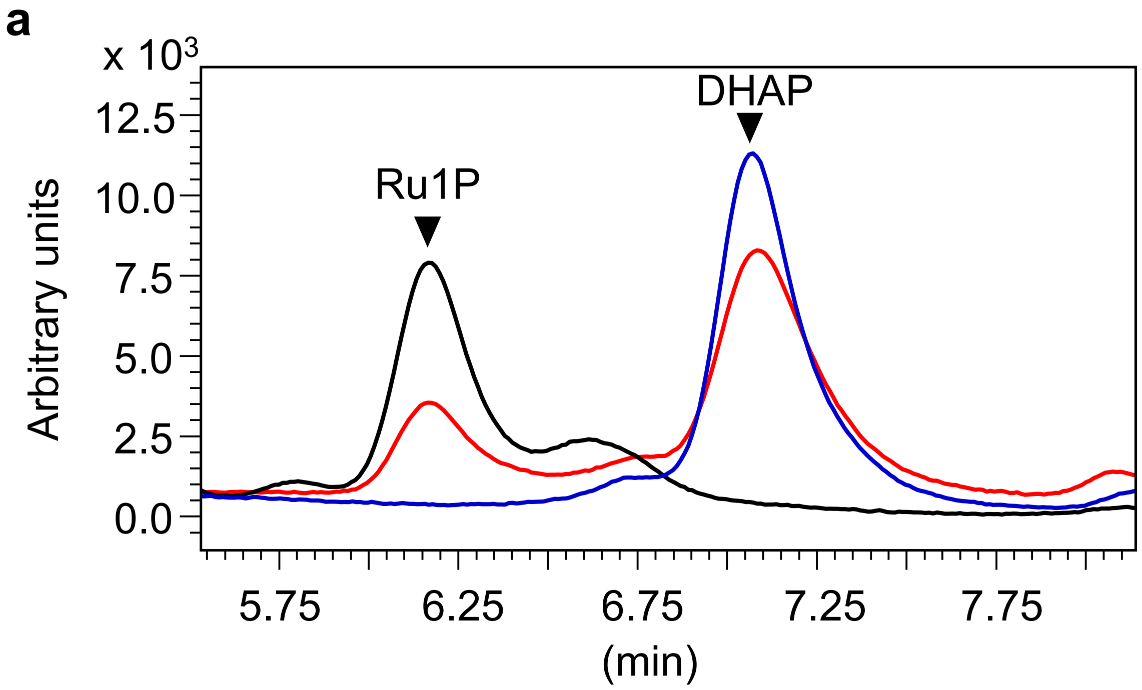

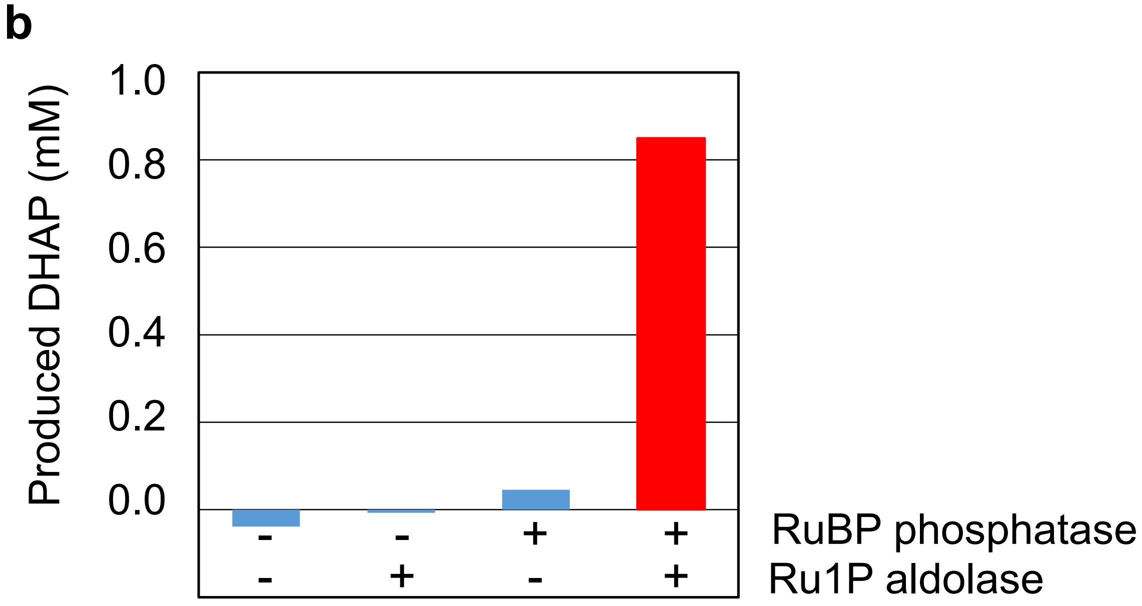

Supplementary Fig. 16. Ru1P aldolase activity of the *Hx*-FucA protein.
a, The *Hx*-FucA protein (4 μg per 100 μl) was incubated with 5 mM DHAP, 25 mM glycolaldehyde, 2 M KCl, and 1 mM $ZnCl_2$ at 47 °C for 20 min. The reaction product was analyzed by HPLC. The elution profiles of standard DHAP (blue), Ru1P produced from RuBP with *Hx*-RuBP phosphatase (black), and the reaction product of *Hx*-FucA (red) are shown. **b,** A coupling reaction with *Hx*-RuBP phosphatase and the *Hx*-FucA protein. DHAP production from RuBP was examined in the presence or absence of these proteins. The reaction was performed at 47 °C for 3 h in the presence of 10 mM RuBP, 5 mM $MgCl_2$, 2 M KCl, 1 mM $ZnCl_2$, and purified proteins (1 μg each per 100 μl). The produced DHAP was quantified with a coupling enzyme, glycerol-3-phosphate dehydrogenase.

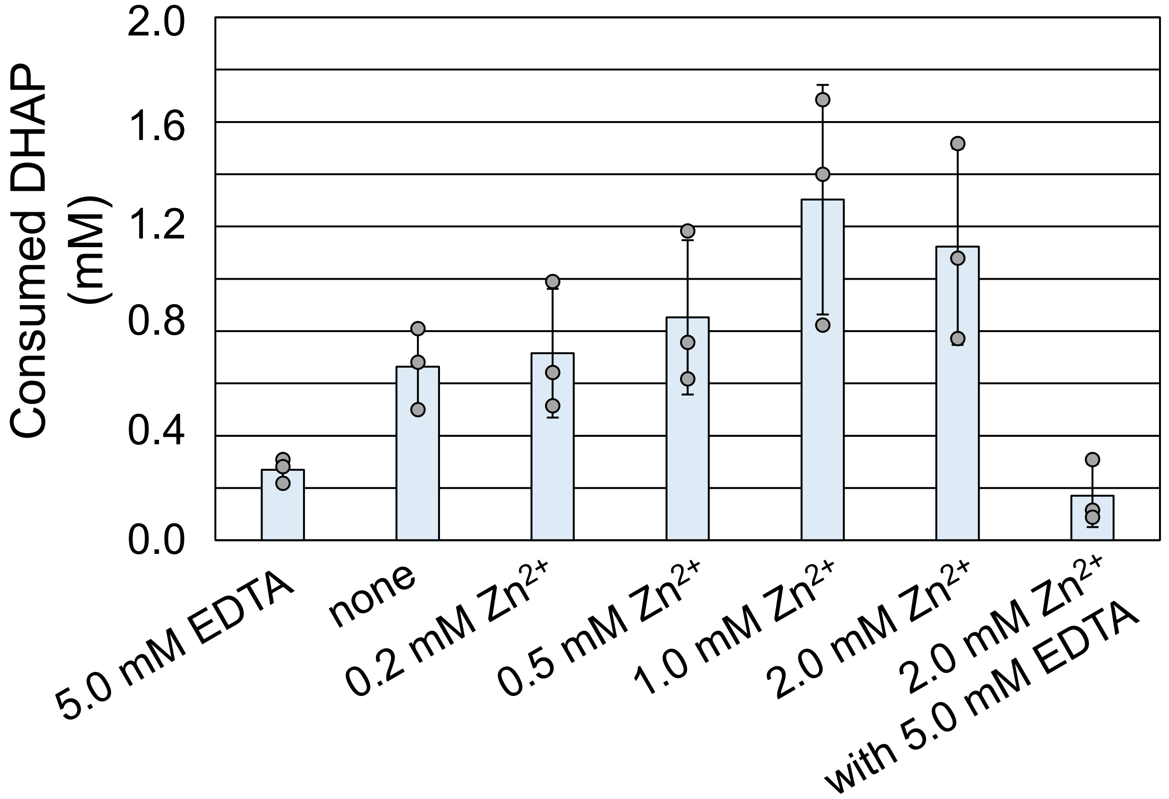

Supplementary Fig. 17. Effects of Zn^{2+} on the aldolase activity of the *Hx*-Ru1P aldolase protein. Effects of Zn^{2+} on aldolase activity of *Hx*-Ru1P aldolase were examined. The aldolase reaction mixture (50 μl) contained 50 mM Tris-HCl (pH 8.0), 5 mM DHAP, 25 mM glycolaldehyde, 2 M KCl, 4 μg of purified enzyme, and 0, 0.2, 0.5, 1.0, or 2.0 mM $ZnCl_2$. When necessary, EDTA was added to the reaction mixture at the final concentration of 5.0 mM. The reaction was carried out at 47 °C for 20 min. Consumed DHAP was quantified by examining residual DHAP with a coupling enzyme. The activities were calculated from three independent experiments. Error bars indicate standard deviations.

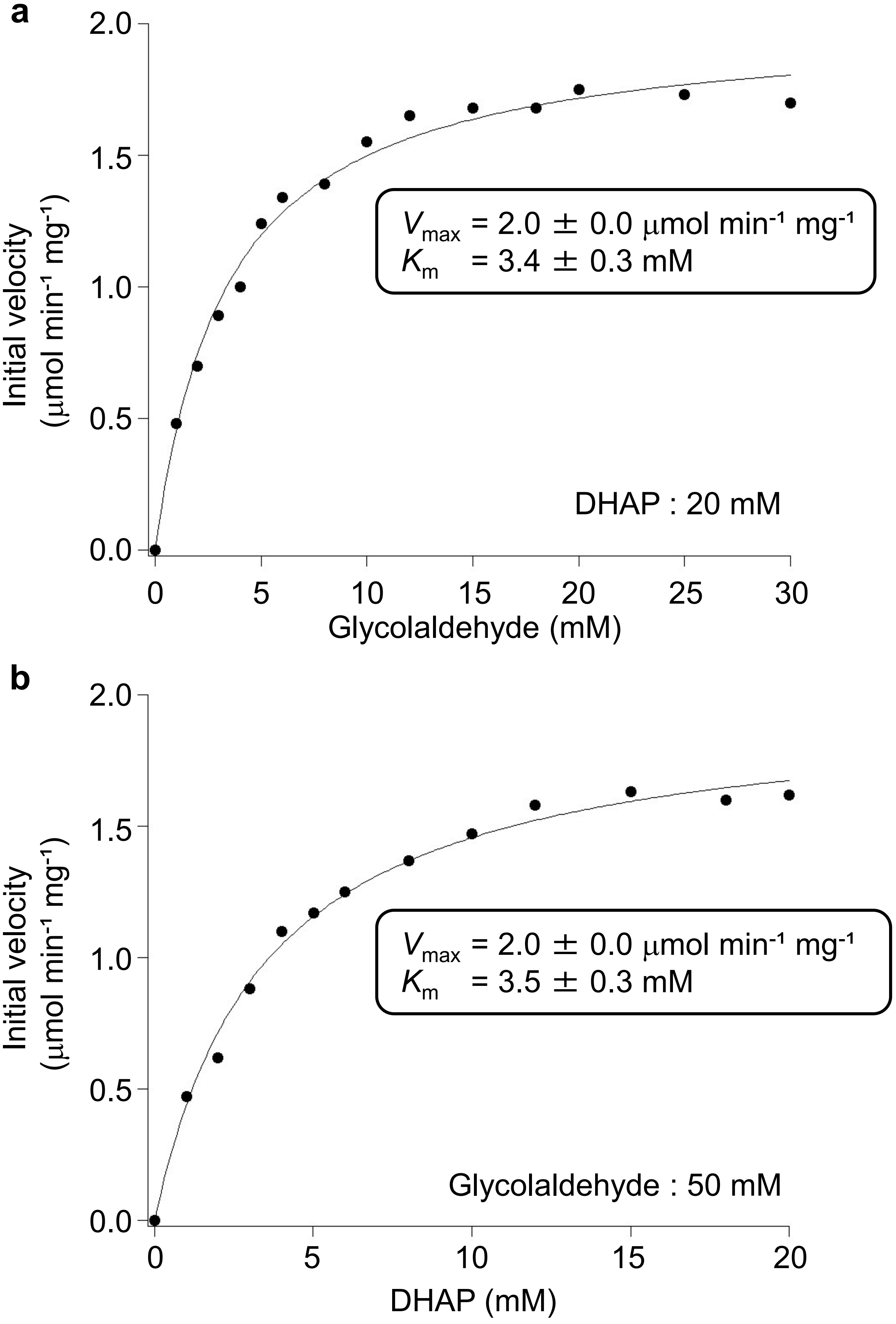

Supplementary Fig. 18. Kinetic analyses of the Ru1P aldolase reaction catalyzed by the *Hx*-FucA protein.

a, Measurements were carried out with various concentrations of glycolaldehyde with 20 mM DHAP. **b,** Measurements were carried out with various concentrations of DHAP with 50 mM glycolaldehyde. Reactions were carried out at 47 ^{o}C in the presence of 2 M KCl and 1 mM $ZnCl_2$. The [S]-*v* plot was fitted with the Michaelis-Menten equation (equation 1).

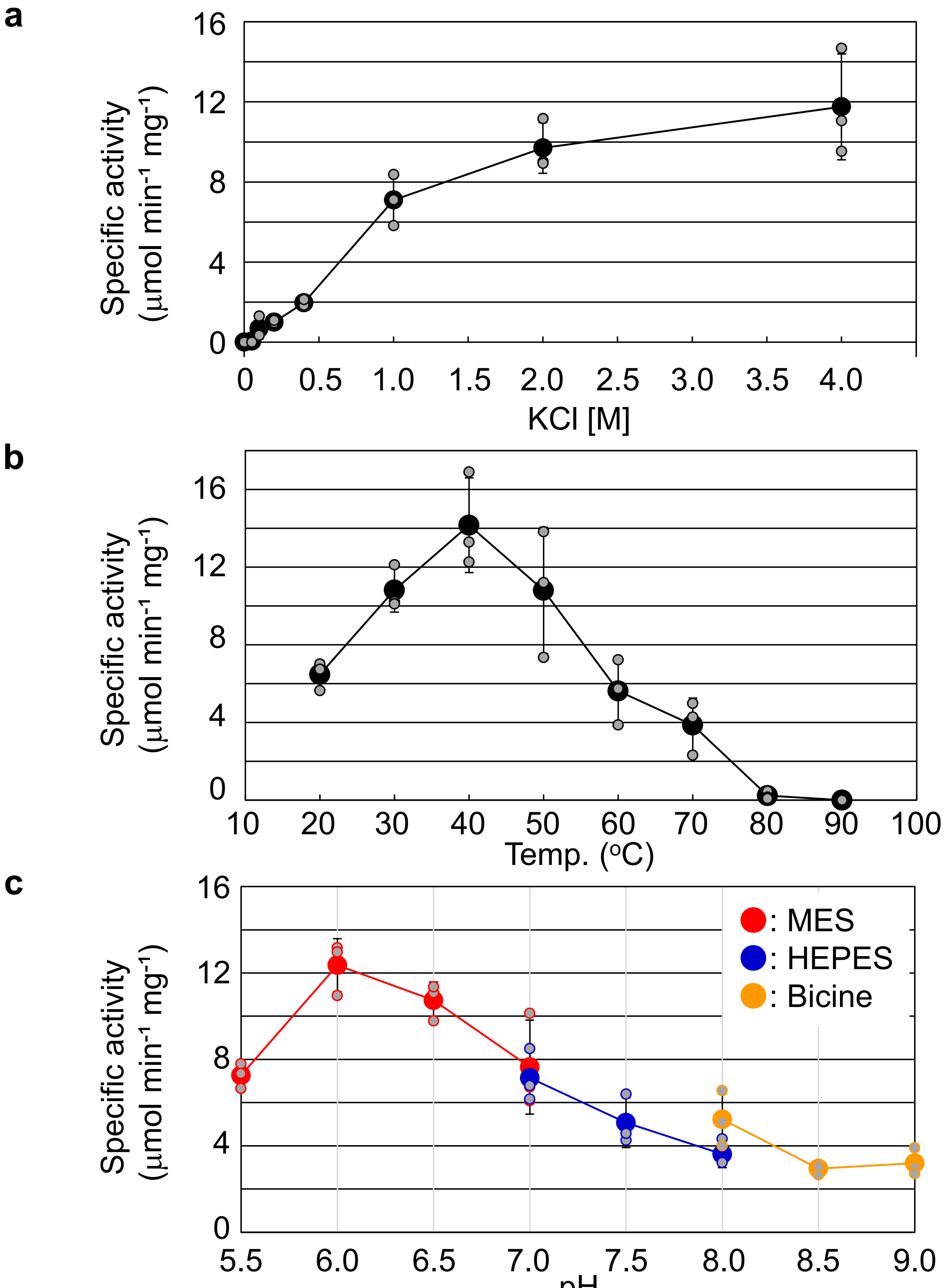

Supplementary Fig. 19. Enzymatic characterization of native *Hs*-GaR. Effects of KCl concentration **(a)**, temperature **(b)**, and pH **(c)** on the glycolaldehyde reductase activity of native *Hs*-GaR. **a**, Reaction mixture included 7.5 mM glycolaldehyde and 0.2 mM NADH in 50 mM MES-NaOH (pH 6.5) and the reaction was carried out at 30 °C. **b**, Reaction mixture included 7.5 mM glycolaldehyde, 0.2 mM NADH, and 4 M KCl in 50 mM MES-NaOH (pH 6.5). **c**, Reaction mixture included 7.5 mM glycolaldehyde, 0.2 mM NADH, and 4 M KCl in MES (pH 5.5-7.0), HEPES (pH 7.0-8.0), or Bicine (pH 8.0-9.0) and the reaction was carried out at 40 °C. The activities were calculated from three independent experiments. Error bars indicate standard deviations.

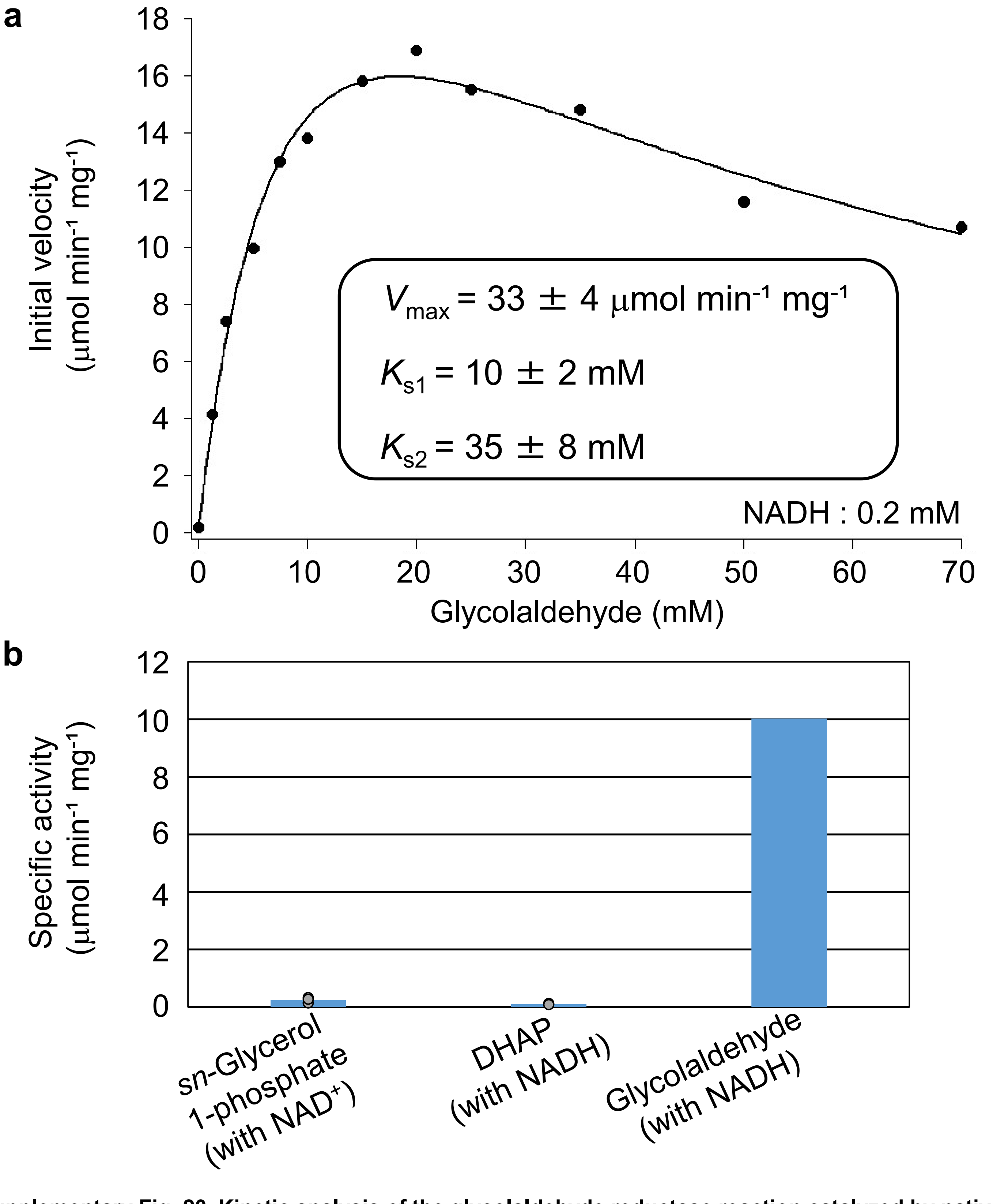

Supplementary Fig. 20. Kinetic analysis of the glycolaldehyde reductase reaction catalyzed by native *Hs*-GaR and its activity towards *sn*-glycerol 1-phosphate and DHAP.

a, Glycolaldehyde reductase activity catalyzed by native *Hs*-GaR at 40 °C in the presence of 0.2 mM NADH, 3 M KCl, and various concentrations of glycolaldehyde. The [S]-*v* plot was fitted with the equation, $v=V_{max}[S]/(K_{s1} + [S] + [S]^2/K_{s2})$ [equation 2], where *v* is the initial velocity, V_{max} is the maximum velocity, [S] is the substrate concentration, K_{S1} is the dissociation constant of substrate and the active site of the enzyme, and K_{S2} is that between a second substrate molecule and the complex of the protein and first substrate. **b,** The *sn*-glycerol-1-phosphate (Gly1P) dehydrogenase activity of *Hs*-GaR. Gly1P oxidation was measured at 40 °C in the presence of 7.5 mM Gly1P, 0.2 mM NAD^+, and 4 M KCl. DHAP/glycolaldehyde reduction was measured at 40 °C in the presence of 7.5 mM DHAP/glycolaldehyde, 0.2 mM NADH, and 4 M KCl. Activities for Gly1P oxidation and DHAP reduction were calculated from three independent experiments. Error bars indicate standard deviations. For glycolaldehyde reductase activity, single experiment was carried out.

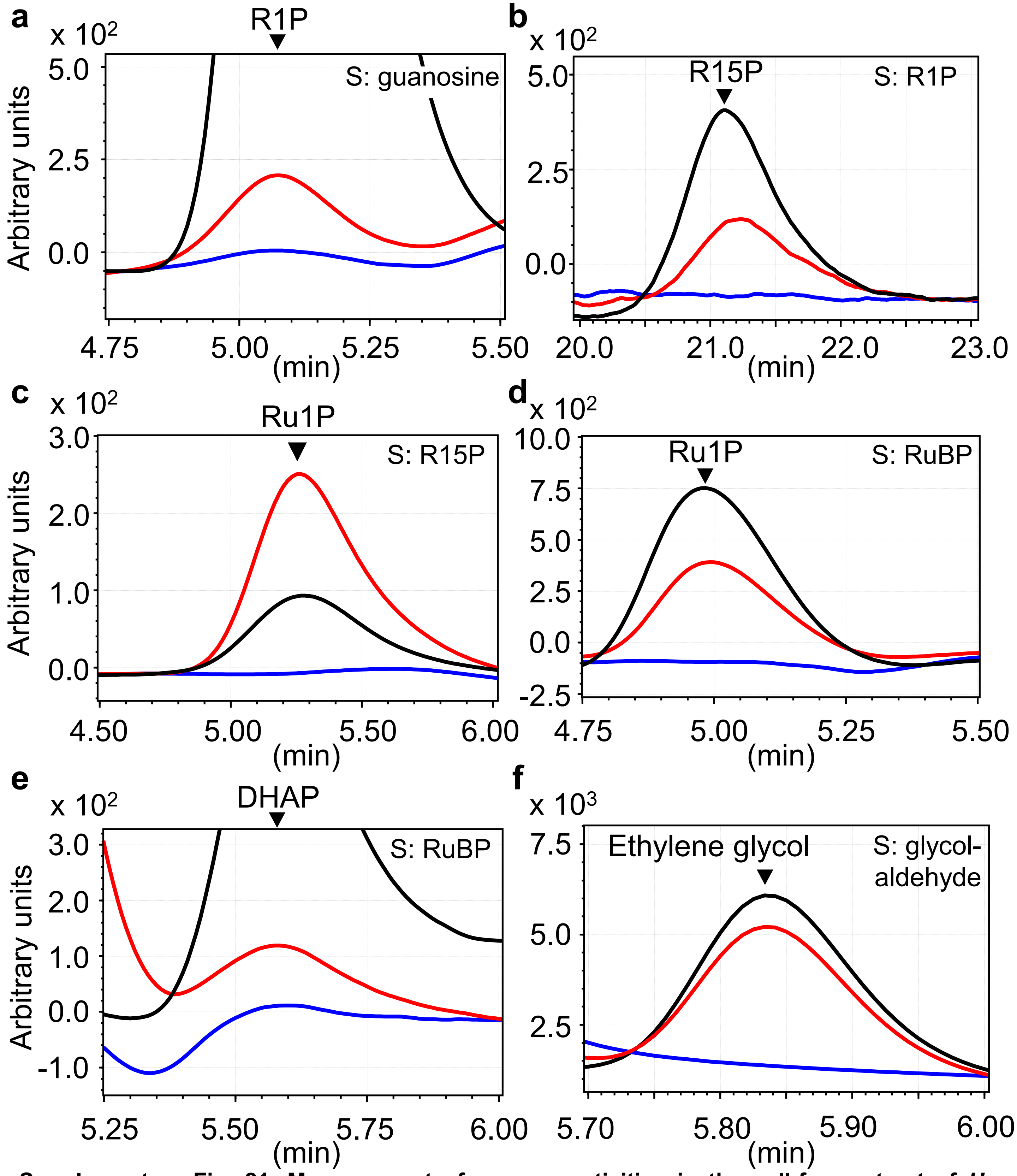

Supplementary Fig. 21. Measurement of enzyme activities in the cell-free extract of *H. salinarum*. a, Guanosine phosphorylase, **b,** ATP-dependent R1P kinase, **c,** R15P isomerase, **d,** RuBP phosphatase, **e,** Ru1P aldolase, **f,** glycolaldehyde reductase activities were examined with HPLC. Black, red, and blue lines indicate product standards, enzyme reaction products with a substrate, and those without a substrate, respectively. Substrates used in the experiments are shown in the upper right corner of each panel. **c,** Instead of RuBP, we examined the generation of Ru1P which can be synthesized from R15P by a coupling reaction of R15P isomerase and RuBP phosphatase. **e,** As Ru1P is not commercially available, the Ru1P aldolase activity was examined by coupling with RuBP phosphatase using RuBP as a substrate. The Ru1P standard (5 mM) in **(c)** and **(d)** was prepared from RuBP by an enzymatic reaction with the *Hx*-RuBP phosphatase recombinant protein. 10 mM R1P **(a)**, 10 mM R15P **(b)**, 5 mM DHAP **(e)**, and 15 mM ethylene glycol **(e)** were used as standard compounds.

>*Ht*-R15P isomerase
ATGGACGATCGTGGTCCGGACGTGGCACCAGCTGTGGTGACCACCGCGGATGAGATCGCAGCGATGGAAATCCGTGGCGC
TGCTACTATTGCCGATGCTGCGGCGGAAGCCCTTGCTATTCAGGCCGAACGCAGTGATGCAGAACGCCCGGACGCTTTCGA
ACGCCAGCTTCGTGCGGCTGCGAAGACCTTATATGAGACCCGTCCGACTGCAGTGAGCTTACCGAATGCGTTGCGTTACGT
CCTGGCCGGCATGGACGGTGAAACCGTGGCCGAGCTGCGTGCGAGCACCATTGCGCGTGCCGAAGAATTTCAGCGTGATC
TGGCGCAGGCGCAAAGCAAACTGGGTAGCGTGGGCGCGAATCGTCTGCGTGATGGCGATGTGGTAATGACCCATTGTCATT
CTACCGACGCCTTGGCTTGCTTGGAGGCAGCGGTAGAGGATGGTACCGAAATTGAAGCGATCGTGAAAGAAACGCGCCCAC
GTCTTCAGGGCCATATTACCGCCCGTCAACTTCGTGAATGGGACGTACCAGTTACCGTGATTGTGGATGGTGCGGCTCGTCG
CTATCTGGATCAGGCGGATCATGTGCTGGTGGGTGCAGACAGCATTGCTGCAGATGGCAGCGTCATCAACAAGATTGGTAC
GTCTGGCTTAGCTGTCATTGCGCGCGAGCGCGGTGTCCCAGTAACCGTTGCGGCCCAAACCATTAAACTGCATCCGGACAC
CATGACCGGCCACACGGTAGAAATTGAACGTCGTGACGAACGTGAGGTGCTGGATGACGATGAACGTGCTGCAATTACCGA
TACTGCTGACGGCGCTGACGACGGTCTGACGGTTGAGAATCCGGCGTTTGATGTCACTCCACCACGTTATGTCGATGCGATT
GTGACCGAACATGGCCAGTTTCCGCCAGAAACTGTGGTTACCCTGATGCGTGAATTGTTTGGCGAGACTGTGGACGAACCAT
GGGAACTGTAG

>*Hs*-RbsK
ATGAGCCGTGTTGTGGCAGCAGGTCATGTGAATTGGGACGTGACCCTGCGTGTCGACGCATTACCAGAACCGGATGGCGAG
GCGGAAATTGTCAGCCAACGTCGTTCTGGCGGTGGCTCTGCTGCGAACGTGGCGTGCGCGCTTGCGGGTTTTGATATTGAT
GCAGGCTTGGTGGGCTCCGTCGGTGATGATGAACATGGTCTGCTTGCGCAGCGTGAACTGCAGGCGGCAGGCGTCGATTTA
GCGGGCCTTCGTGTGGTGGATGGCCTGGAAACCAGCGTCAAATATCTGCTGGTTGATTCCCGTGGCGAAGTGATGGTACTT
GGTAACGATGGCGCGAATGAGGCCGTGGCCCCAGGTGATCTTGATCCGGCGGTGGTCGGTGCAGATTGCGAGCATGTGCA
TCTGACCTCGCAACGTCCGGCGACCGCGGCTCGTTTAACCGCTCTTGCCCGTGAGCACGATGCGACCGTGTCGTTTGATCC
AGGTCGTCGCTTACGCGATCGTGATTACGGCGACGCCCTTGCGCGTACGGACATCTTATTTGTGAATGACAACGAGGCTGG
CGCGGTATTAGATGACGACGGTGGTCCAACCGCACCAGATAGCGACCGTGTTGTAGTGGTGAAACATGGCAGCGAAGGCGC
GACGGTCCACACTCCAAGCGGTAGTGTCACTCACCCAGGCTTCGCGGTTGACCCAGTGGACACCACCGGTGCGGGGGACG
CTTTTGCTGCGGGCTTCGTCGCAGTCGTGCTGGATGGTGGGGATCATGAACGTGCGCTGGAATTTGGCAACGCTTGTGGCG
CGTTAGCCGCCCAACATGAAGGTGCTCGTACTGCGCCAACTCGTGCGGATGCCGCATCCTTTCTGGATTCGCAGTTTTGA

>*Ht*-RbsK
ATGCCGCCAACCGTGCTGACTGCGGGCCATGTGAATTGGGACGTGACCCTGCGTGTCGACCGTCTTCCGGCTGCAGACGGT
GAAGCCACCATTCGTAGCCAACGTCAAAGTGGCGGTGGTTCTGCAGCTAATGTTGCAGCAGCTCTTGCGGGTTTAGAAGTAG
ATGCAGGCTTGATTGGCAGCGTTGGCGACGACGATAACGGTCTTCTGGCCCGTCGTGGCCTTGAAGAGGCGGGTGTAAGCC
TGAGCGGCATTCGTGTCGTCGAAGGCGCAGAAACGGCGGTGAAATACTTGCTGGTGGACGATGATGGTGCAGTGAGCATTC
TGGGCAATGACGGCGTGAATGAAGCCGTGCGTCCGGCGGATATTGACGCAGATCGTGTGCGTGCAGCGGACCACGTGCAT
CTGACGAGCCAGCGTCCAGAAACCGCAGCGCGTATTGCCTCTATCGCGGCAGCCGCGGACGTTACCGTGAGCTTTGATCCA
GGCCGTCGTTTTGGTGATCGTACTTATGATGAGACCGTAGCAGCGGCCGATATCCTGTTTGTGACGGAACATGAGGCCGCA
GCATTGGTGGATGCTGCTGGTGTCGATAGTGAGCCGACCGATCGTATCGTGGCGATTACCTCTGGTGGCGATGGTGCGGAG
ATTCGTACCCCAGATGATAGCTACGCACATCCGGGTTTCGACGTAGATAGCGTGGATACCGCTGGCGCTGGTGATTCCTTTG
CAGCTGGCTTTCTGACCAGCCGTCTGGAGGGCGCGTCCATTGAAGATGCGCTGGAATATGCGAATGCATGTGGCGCGTTGA
CGGCATCCCGTGAGGGTGCACGTTCTGCACCAACCGCAAGCGAAGTCGAGCGTTTCTTAGAGGAGCGTGCGGGCTGA

>*Hx*-RbsK
ATGGTCCGTGTGCTGTCCGCTGGTCACGTTAATTGGGATGTGACCCTGCGTGTGGATCGTCTGCCAGAAGCCGACGGCGAA
GCCAGCATTCGTTCTCAGCGTCAGAGCGGCGGTGGTTCTGCGGCAAATGTTGCGGCTGCGCTTGCGGGTCTGGAGGTTGAT
GCGGGTCTGATTGGTAGCGTGGGTGATGACGACAATGGCGTACTGGCGCGTCGCGATCTTGAGTCTGCCGGTGTAGATCTG
GAAGGCGTGCGTATTGTGGAGGCAGGCCAAACCGCCGTCAAGTATCTTCTGGTGGATGATGATGGCGAGGTGGCGGTGCTT
GGCAATGATGGTGTCAATGAGGCGGTTGGTCCAGAAGAAATTGATGAACGTCGTATTCGTAATGCGGATCACGTGCATCTGA
CCAGCCAGCGTCCGGATACTGCAGCAGCGATTGCGCGTACCGCGAACGAAGCGGGTGTGACCGTGTCTTTTGACCCGGGT
CGTCGTCTGGGTGATCGTGATTATGGTGAAGCGCTGGCCGCTGCGGATGTTTTGTTTGCCAACGACCGTGAGCTTGCTGCTC
TGCTGGAAGATGAGTATGAATACGTGGGTAGCGATTTTGATGATCGTATTGTCGCGGTGAAACATGGCAGTGACGGTGCGGA
AGTGCATACCCCGACCGGCTCTTACGTACATCCGGGTTTTGACGTCGATGCGGTGGACACTGCAGGCGCTGGTGATGCGTT
TGCAGCTGGTTTTATTGCGACCTGGCTGACTGATGGTGACGTAGAACGTGCACTTGAATATGCGAATGCCTGCGGTGCCCTG
ACCGCCGGTCGTGAAGGTGCTCGTAGCGCACCAACTGCGGATGCAGTGGCGGCTTTCCTGAGCGAGCGTTTTGATTAA

>*Ht*-Urdpase1
ATGGCCACCCAGCCACACCTGCTGGTAGATGACGGTGATTTAACCGATCGTGTGCTTGTCCCAGGTGACCCGGGTCGTGTA
GATCGTATCGCGGACCATTGTGATGAATCTGAGACGGTGGCGCAGAATCGTGAGTACAAAGTGGTGAATGCCACGTACGAG
GGCCAGGAACTGACCATTTGCAGCACCGGTATTGGCTGCCCGAGCGCGGCAATTGCAGTGGAGGAGCTTGCGAATGTTGGC
GTTGAAACTATCATTCGTGTGGGCACTACTGGCGCACTGCAGAGCGGTATCGAAATTGGTGATATGGTTGTGGCGACCGGTG
CTGCCAAGGAAGAAGGCACTTCCAAACGTTATGAGGCCGCAGAGTACCCAGCTGTGCCAGACTACGATGTCTTAAGCGCATT
GGTAGACTCCGCTGAGGCCAACGGTGAAGATGTGCATGTAGGCCCGATTGTCTCCGATGATGCGTATTATGCGGAGACCGA
TGAACATGTCGTCGATTGGGAGGCTGCGGGCCTGTTAGCCGTGGAAATGGAGGCAGCGGCTATTTTCTCTCTGGCGCGTCG
TAAAGGCTTGCGTGCAGGTGCCATCTGCACTGTTGATGGTAACCTGGTCGAAGGTACCCAGAAGGGTACTGACACCGAGGA
CGACGAGCTGCCGGAGAAGGCGAAGAACAATGTGGGTCGTGCGATTGACATTAGCCTTGAAGCCGCAACCCGTCTGTAG

Supplementary Fig. 22. The nucleic acid sequences of each gene designed for expression in *E. coli*. To decrease their GC contents and optimize their codons to enhance gene expression in *E. coli,* genes were designed as shown here and chemically synthesized.

Continued Supplementary Fig. 22

>*Hx*-Urdpase1
ATGGCACCCAACCGCACCTGCTGGTTGAGGAAGGCGACCTGAACGATATTGCGCTGATCCCGGGTGACCCAGGCCGTGTA
GACCGTATCGCGGATCACTGCGACGAAGCGGAAACCGTTGCGCAGAACCGTGAATACAAGCTGGTGAACGCAACCTATGAA
GGTCGTGATCTGACCATCTGCTCTACGGGTATCGGTTGTCCGTCTGCGGCAATCGCTATCGAGGAAATGGCGAACGTAGGT
GTTGAAACCTTCGTTCGCGTTGGCACCACTGGCGCGCTGCAGTCTGAAATCGAAATCGGCGACATGATTGTTGCGACCGGT
GCGGCTAAAAATGAGGGTACCTCTAAACGTTACGAGGACGCGGAATACCCGGCAGTTCCGGACTACGACGTTCTGTCCGCT
CTGGTAGACTCCGCCGAGACCAACGATGAGGACGTTCACGTCGGTCCGATCGCAAGCGACGACGCCTTCTACGCCGAAACT
GATGAATACGTTGCGGACTGGGAAGATGCGGGTCTGCTGTGCGTTGAGATGGAAGCAGCGGCGGTTTTCACCCTGGCACGC
CGTAAAGGTCTGCGTGCGGGTGCGATCTGCACCGTTGACGGCAACCTGGTCGAAGGTACTCAGAAAGGTACCGACACCGAA
GATGACGAGCTGCCGGACAAAGCGAAGAACAATGTTGGTCGTGCGATTGACATCGCACTGGAGGCCGCGACGGACCTGTAA

>*Hl*-Urdpase1
ATGGCGAAACAGCCGCATCTGCTGGTAGAGGAAGGTGACGTTCACGAGATTGCGATCATCCCGGGTGATCCGGGTCGTGTA
GACCGTATCGCCGACCTGTGCGACGACTCCGAACTGGTTGCGCAGAACCGTGAATACAAAATCGTCAACGCGTCTTATGATG
GTACTGACCTGACCATCTGTTCTACCGGCATCGGTTGCCCGTCTGCGGCTATCGCGGTTGAAGAACTGTCTCGTGTTGGTGT
TGAAACGTTCCTGCGTTGCGGTACCTGTGGTGCGCTGCAAGCGGACATGGAAGTTGGTGACATGGTTGTTGCGACCGGTGC
GGCGAAAGAAGAGGGCACCTCTAAACGTTACGAAAGCGTTGAATACCCAGCGGTTCCGGACTACGACGCGCTGACGGAGCT
GGTTGGCGCAGCGGAGGACAACGATGAAGAAATCCACGTTGGTCCGATCGTCTCCGACGACGCCTTCTACAACGAATCTGA
TGAATACGTAGATGACTGGAACGATGCGAACCTGCTGGCGATTGAAATGGAAGCGGCGACCGTTTTTGCGCTGGCTCGCCG
TAAAGGTCTGCGTGCGGGTGCTATCTGTACCGTCGATGGTAATCTGGTAGCGGGTAACCAGAAAGGTGCGGACTCTGACGA
CGAGCTGCCGGAAAAAGCCAAAGATAATGTTGAGCGTGCAATCCGTATCACCCTGAACGCGGTTACCGCGCTGTAA

>*Hx*-HAD hydrolase
ATGGCGACCGCAGTGCTGTTTGATCTGGACAACACCCTGTATCCGTATCCGCCGTGCAATCAAGCGGGCAAAGCGGCAGCT
CTGGAACGTGCGCAGGAACTGGGCTACGATTTTGATCATGAAAGCTTCGCGGAATTCTATCAGGCAGGCCGTCGTGAAGTGA
AACGTGATACCGGTGGCACCGCTGCCTCTCATGAACGTTATCTGTACTTTAAACGTGCTTTGGAACTGCACACCGGCTCCCC
ACGTCCAGGCGATGCGCTGGCCCTGGGTGATGCGTACTGGAGCGCGTATCTGGAAGAAATGAGCCTGGTGCCGGATGCGA
AAGAGACCCTGGAGGAGTTACAAGAACAGGGCGTGGATATTGCTATTACCACCAATCTGACCACCACCATTCAGCTGGCGAA
ACTTGAGCGTCTTGGTCTGACCGATTATGTGGATCTGGTACTGACCAGCGAAGAAACCGGTCAGGAAAAACCGGCGAGCGT
GATGTTCACCCTGCCACTTGCGCGCCTGGATTCTCGTGCGAGTGAAGCGGTGATGGTCGGCGACGACTTAGAGGCGGACAT
TGCAGGCGCTAATGCGGTGGGCCTGGAAACCGTGTTATTTGATCCGAGCGAGGAAAGCGATGCCACCGAAAGCGCGGATC
GTGCGGCGACTGAACGTCAGGCGGATCATAGCATTGATACCCTGGGTGAACTGACTGATTTGGTGAGCTAG

>*Hx*-FucA
ATGATCCTGGAAACGGAACGTCGCGCGGTGGTGGAACATGCGAGCGAGCTGGCCGATCTGACCCCGGGTCGTACCGGCAA
TCTGAGCGTGCGTGGTAGCGGTGATGATACCGATGCTGGCCCAGGCGACGCATTTGCGATTACCCCGACCGGCGTGCCGTA
TGATGGCTTTGACGTGGAGGACGTGCCGGTGGTCGGCACCGATGGCGAACGTCTTGATGGTGAGATGGCGCCGAGCAGCG
AAGTGCCGATGCATAGCGCCATTTATCGTCGTGAGGATGTGGGCGCGATTGTGCATACCCATAGCCCATGGGCGACTGCGC
TTGCGGTTGCGAATCAGCCGCTGCCGCCGATTCATTATATGATTGTGGCGGTGGGCAAGCGTGTGCCAGTGGCGGAATATG
CGCCATACGGTACTGACGAACTGGCGGAGAATATTGTTACCGCGATGACCGAAGCGGGTAGCACTGCGTCTCTGATTGAAAA
TCATGGCCTGGTGGTGACCGCGCCAGACTTGGAAACCGCGCTGGAAAATACTCATCATGTGGAAAGTCTGGCGCGTCTGTA
TCTGGAAAGCCGTAGCGCAGGCTTGGAGCCGCAGACCCTGACCGATGATCAGCTGGAGACCGTGTTAGAAAAGTTTGAAAG
CTATGGCCAGTAG

>*Hs*-GaR
ATGACCAGCGTTTTTAAAAGCCCGAGCACCTATGTTCAGGGTCGTAATGTTACCACCGATATTGGTACCCATGCGGAAAGCCT
GGGTGATACCGCGCTGCTGGTTGCGGATGAAATTGTTATGGATATGATTGAAAGCGATGTTCATGAAAGCCTGGCGGAAGCG
GGTCTGGATGGTAGCAGCGTTGTTTTTAATGGTGAAAGCAGCGAAGATGAAATTGAACGTATTGCGAGCGTTGCGGTTGATG
AAGGTGCGGATATTGTTATTGGTGCGGGTGGTGGTAAAGCGCTGGATACCGCGAAAGCGGTTCGTGAAGAAGTTGGTGGTG
CGATGGTTAGCATGCCGACCATTGCGAGCATGGATGCGCCGACCAGCAGCCTGAGCGTTATTTATAGCGAACATGGTGAATT
TGAAGATTATTGGTATTATGAACAGCATCCGGATCTGGTTATTGTTGATACCGAAGTTGTTGCGGCGGCGCCGGCGCGTTTTC
TGCGTAGCGGTATTGCGGATGGTCTGGCGACCTGGTTTGAAGCGGATGCGGTTGCGCAGAGCGGTGGTGATAATGAAGTTG
GTGGTAAACCGACCCGTGCGGGTCATAAACTGGCGGAACTGTGTTATGAAACCCTGCGTGAACATGGTGCGGGTGCGCTGG
ATGCGGTTGAACATGATGCGGTTACCGAAAGCGTTGATGCGGTTATTGAAGCGAATACCCTGCTGAGCGGTCTGGGTTTTGA
AAGCGGTGGTCTGGCGGCGGCGCATAGCGTTCATAATGGTCTGACCCAGCTGGCGGAAACCCATGATGCGACCCATGGTGA
AAAAGTTAATATTGGTACCATTACCCAACTGGTTCTGGAAGGTCATAGCGATGCGCGTATTGAAGAATTTATTGAATTTAGCGT
TGAACTGGGTCTGCCGGTTACCCTGGGTGAAATTGGTATTACCAATCCGGAACAGGTTGATCTGGATGTTGTTGCGGAAGCG
GCGTGTGATGAAGCGGAAACCATTCATGATGAACCGTTTGATGTTACCCCGGCGATGGTTCGTGATGCGCTGCTGACCGCG
GATGAAATGGGTCGTCGTGTTCGTGATCGTTAA

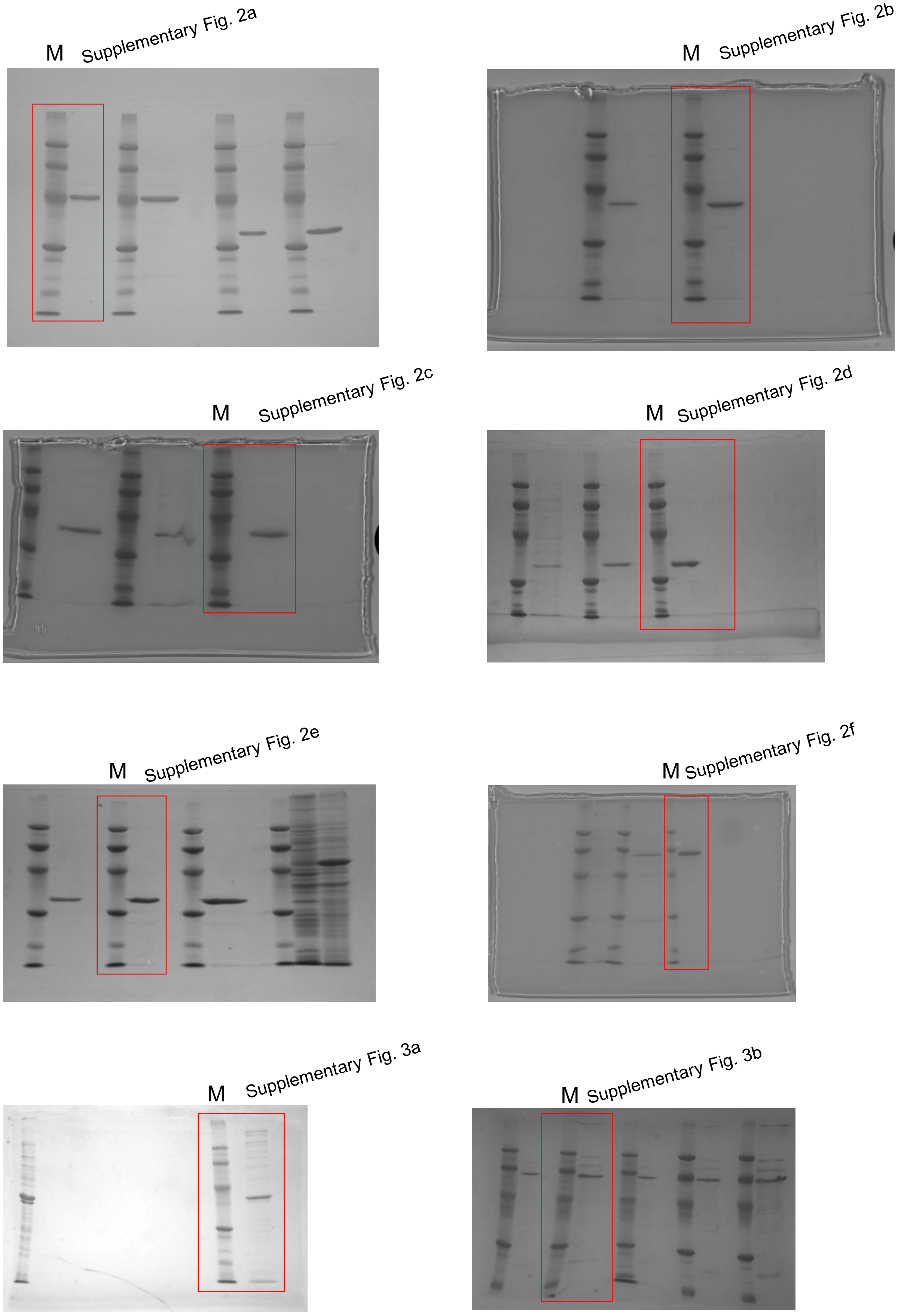

Supplementary Fig. 23.
Uncropped gel pictures including all pieces of gel pictures shown in Supplementary Figs. 2 and 3. Red rectangles approximately indicate the position of gel pictures in Supplementary Figs. 2 and 3.
