# Supplementary material for: A non-carboxylating pentose bisphosphate pathway in halophilic archaea
Source: Commun Biol. 2022 Nov 24;5:1290. doi: 10.1038/s42003-022-04247-2 (PMC9700705; doi:10.1038/s42003-022-04247-2)
Supplement: Supplementary file 2 — Description of Additional Supplementary Data [file 42003_2022_4247_MOESM2_ESM.docx]

**Description of Additional Supplementary Files**

**File name:** Supplementary Data 1

**Description:** The source data behind the graphs in the paper.
